# A New Look at Care in Pregnancy: Simple, Effective Interventions for Neglected Populations

**DOI:** 10.1371/journal.pone.0160562

**Published:** 2016-08-18

**Authors:** Stephen Hodgins, James Tielsch, Kristen Rankin, Amber Robinson, Annie Kearns, Jacquelyn Caglia

**Affiliations:** 1 Saving Newborn Lives, Save the Children/ US, Washington, D.C., United States of America; 2 Milken Institute School of Public Health, George Washington University, Washington, D.C., United States of America; 3 Department of Life Sciences, Brunel University London, London, United Kingdom; 4 Human Care Systems, Boston, Massachusetts, United States of America; 5 T.H. Chan School of Public Health, Harvard University, Boston, Massachusetts, United States of America; Stellenbosch University, SOUTH AFRICA

## Abstract

**Background:**

Although this is beginning to change, the content of antenatal care has been relatively neglected in safe-motherhood program efforts. This appears in part to be due to an unwarranted belief that interventions over this period have far less impact than those provided around the time of birth. In this par, we review available evidence for 21 interventions potentially deliverable during pregnancy at high coverage to neglected populations in low income countries, with regard to effectiveness in reducing risk of: maternal mortality, newborn mortality, stillbirth, prematurity and intrauterine growth restriction. Selection was restricted to interventions that can be provided by non-professional health auxiliaries and not requiring laboratory support.

**Methods:**

In this narrative review, we included relevant Cochrane and other systematic reviews and did comprehensive bibliographic searches. Inclusion criteria varied by intervention; where available randomized controlled trial evidence was insufficient, observational study evidence was considered. For each intervention we focused on overall contribution to our outcomes of interest, across varying epidemiologies.

**Results:**

In the aggregate, achieving high effective coverage for this set of interventions would very substantially reduce risk for our outcomes of interest and reduce outcome inequities. Certain specific interventions, if pushed to high coverage have significant potential impact across many settings. For example, reliable detection of pre-eclampsia followed by timely delivery could prevent up to ¼ of newborn and stillbirth deaths and over 90% of maternal eclampsia/pre-eclampsia deaths. Other interventions have potent effects in specific settings: in areas of high *P falciparum* burden, systematic use of insecticide-treated nets and/or intermittent presumptive therapy in pregnancy could reduce maternal mortality by up to 10%, newborn mortality by up to 20%, and stillbirths by up to 25–30%. Behavioral interventions targeting practices at birth and in the hours that follow can have substantial impact in settings where many births happen at home: in such circumstances early initiation of breastfeeding can reduce risk of newborn death by up to 20%; good thermal care practices can reduce mortality risk by a similar order of magnitude.

**Conclusions:**

Simple interventions delivered during pregnancy have considerable potential impact on important mortality outcomes. More programmatic effort is warranted to ensure high effective coverage.

## Introduction

Since the work by Villar[1], Carroli[2] and colleagues at WHO almost 15 years ago, there has been relatively little attention in the maternal-newborn health community to the content and potential impact of antenatal care (ANC), although this is beginning to change. Over this period, maternal health program effort has largely focused on labor and delivery and, although ANC1 and ANC4+ indicators are routinely used as overall proxy measures for maternal health program performance (along with “skilled birth attendance”), the specific interventions delivered during pregnancy (packaged as “focused ANC”) have been—in terms of programmatic attention from the maternal health community—very much poor cousins to emergency obstetrical care and efforts to increase skilled birth attendance. Similarly, the newborn community has given relatively little attention to opportunities during pregnancy to reduce newborn and stillbirth deaths, as reflected in the minimal emphasis given to antenatal care in the global Every Newborn Action Plan[3]. Some would argue that this relative neglect of antenatal care is justified because real mortality reduction impact can only be achieved through effective services targeting the period around labor and delivery and indeed relative neglect of antenatal care has had the consequence that the actual content of antenatal care that is provided has tended to be grossly inadequate in low resource settings[4, 5]. But is it really true that there is little impact to be expected from ANC? This paper seeks to build a case that, in fact, there is significant potential for reducing mortality when key interventions provided during pregnancy are delivered at high coverage, particularly among disadvantaged populations.

There have been a number of systematic reviews which have looked at the evidence across a range of interventions including those considered in this paper, though generally not specifically with a primary focus on the antenatal period. They have considered a variety of endpoints, including newborn and perinatal mortality, for example Bhutta et al.[6] which looked at *strength of evidence* for interventions delivered at “community” level; with the same group extending their analysis [7, 8] to look at *effect sizes* and *costing*. Based on their review, these investigators estimated a reduction in all-cause newborn mortality of 10–50% for a “family care package” of interventions, and of 10–20% for a basic “antenatal care package” (10–30% in malarious areas, with intermittent presumptive treatment (IPTp) included in the package). They further developed this analysis in a Cochrane review published in 2010[9] and in subsequent similar reviews which included analysis both for newborn and maternal outcomes[10, 11].

Multiple-intervention reviews have been done specifically with *stillbirth* as endpoint[12–14]. Another systematic review included—in addition to stillbirths–*prematurity*, *low birthweight* and *perinatal death*[15]. The authors found moderate to high quality evidence for efficacy and appropriateness in low and middle-income country (LMIC) settings for: smoking cessation, progesterone, balanced protein energy supplementation, screening and treatment of syphilis, intermittent presumptive treatment for malaria during pregnancy, insecticide-treated mosquito nets, birth preparedness, as well as several aspects of care of the newborn that can be the focus of antenatal counseling, including appropriate thermal care and early initiation of breast-feeding.

This paper reports on a review of a set of simple interventions delivered during pregnancy, of relevance to low-income countries, that can in principle be offered by peripheral-level health workers like auxiliary nurse- midwives and through outreach services. With the relative neglect of content of ANC, it is particularly disadvantaged populations that end up deprived of effective ANC. This review considers potential content for ANC that can be delivered through less robust systems to otherwise poorly reached segments of the population at high coverage. Our review seeks evidence on the expected benefits of these interventions on: 1) mortality endpoints, notably maternal and newborn death, and still-birth; and on 2) intermediate endpoints known to be associated with mortality risk, including—intrauterine growth restriction, prematurity, maternal anemia, and HIV transmission. We also extend our discussion beyond evidence for efficacy to important contextual factors that should drive program prioritization, including locality-specific disease burden and health systems capacity.

The interventions retained for review are simple enough to be used at even the most peripheral level of care, without laboratory support, and therefore lend themselves to delivery even by non-professional health workers and on an outreach basis. They are grouped as: 1) nutrition-related, 2) infection-related and 3) obstetrical or other. In several cases we have retained interventions that are delivered programmatically during pregnancy but are actually applied only after birth (notably in the case of home deliveries without the presence of a health worker). Such interventions would not normally be seen as part of antenatal care; however, in settings where a large proportion of births still take place at home, contact with the health system during pregnancy—either through ANC or community health workers—offers an available platform for delivery. As well as clinical interventions, the review includes key behaviors or household practices. For the behaviors retained, our focus is primarily on their association with our endpoints of interest, not with the effectiveness of behavior change strategies, which may be highly context-dependent. The interventions considered by this review are outlined in Table 1 (numbered to correspond to the sections of the paper where they are addressed).

**Table 1:** Interventions addressed in this review.

Nutrition	Infection	Obstetrical and other
• Immediate & exclusive breastfeeding	• Deworming	• Advance distribution of misoprostol for use immediately after childbirth for preventing post-partum hemorrhage
• Antenatal iron supplementation & anemia screening & treatment	• Intermittent presumptive treatment of malaria & use of insecticide-treated nets	• Pre-eclampsia screening & treatment
• Multi-micronutrient supplementation	• Tetanus toxoid	• Clean delivery practices
• Calcium supplementation	• Advance distribution of chlorhexidine for application to the umbilical cord stump	• Thermal care
• Antenatal vitamin A supplementation	• Syphilis screening & treatment	• Birth preparedness & complication readiness
• Advance distribution of vitamin A for administration to the newborn	• HIV screening/ PMTCT	• Tobacco, alcohol use
• Iodized salt use		• Family Planning
• Balanced protein-energy supplementation		

### Effects Modified by Context

For each of these interventions, the review addresses: 1) evidence on effects with regard to mortality and other important endpoints and 2) variation in expected effects by context. In general, for the interventions considered, there are no universal effect sizes that apply to all populations; instead, in every setting the actual impact of implementing a particular intervention will be the product not only of the intervention but of the full set of necessary conditions (or support factors[16]). This is an important point both for a more nuanced and realistic understanding of causation and—practically—as a basis for prioritizing what is provided in any particular setting. It is a point often missed in typical systematic reviews or meta-analyses that attempt to estimate an “average” treatment effect for a given intervention when such an “average” effect actually applies to no real population. Across settings, there is variability in the proportionate contribution of the major categories of immediate causes of death (including “indirect” causes) and in intermediate risk conditions. The impact of any specific intervention in any particular setting can be expected to vary as a function of:

- overall rates of mortality (stillborn, newborn, maternal, etc.),
- proportion of such deaths attributable to specific immediate causes,
- etiologic fraction attributable to specific underlying cause or risk factor in that setting,
- efficacy of the intervention with regard to mitigating relevant causes or risk factors, and
- coverage in that setting of other interventions that can exercise effects within this causal web (for example, if there is high effective coverage for an associated *treatment* intervention, the effect of pushing up coverage of a corresponding *preventive* intervention will be more modest than in a setting where treatment coverage is lower).

To illustrate variation across settings, a high proportionate contribution to cause-specific mortality is seen in specific regions, as follows[17]:

- Malaria: eastern, central and western Sub-Saharan Africa
- Tetanus: in localized settings with low tetanus toxoid (TT2+) coverage, e.g. northern Nigeria (and, more generally South Asia, eastern and western Sub-Saharan Africa)
- Syphilis: western, central and especially *eastern* sub-Saharan Africa (also Caribbean)
- Infant sepsis and lower respiratory infection: throughout sub-Saharan Africa (lower in southern Sub- Saharan Africa); also South Asia, Oceania, North Africa and Middle East, and Caribbean
- Preterm birth: especially South Asia, central and western Sub-Saharan African, also elsewhere in sub- Saharan Africa, Oceania, Caribbean, north Africa and Middle East
- Nutritional: especially South Asia and central and western Sub-Saharan Africa, but also elsewhere in sub-Saharan Africa; also North Africa and Middle East, Central Asia, Oceania, Caribbean.

### Intermediate Endpoints

In this review, we consider a number of interventions which potentially can reduce risk of the mortality endpoints of interest for this review and which, if delivered at high coverage, contribute to reducing inequities in outcomes. In some cases although we *may not* have direct evidence of such mortality effects, we *may* have evidence for influence on intermediate conditions known to be associated with mortality endpoints of interest, so in addition to mortality endpoints this review will also consider preterm birth, intrauterine growth restriction and maternal anemia. There will also be limited attention given to important cognitive/ developmental outcomes. It is important to note that for maternal death as an endpoint, evidence is thin owing to the relative rarity of the outcome and the associated limitations in statistical power of most available data.

The 2 main intermediate risk conditions retained as outcomes for this review are preterm birth (PTB) and intrauterine growth restriction/ small for gestational age (SGA). In a review by Katz[18], pooled analysis from 20 cohorts across Sub-Saharan Africa, South Asia, and Latin America/ Caribbean (LAC), with over 2 million births, found that PTB was associated with an elevated risk for neonatal mortality of RR = 6.82 (95% CI:3.56–13.07) and for post-neonatal mortality of RR = 2.5 (95% CI: 1.48–4.22). Similarly, for SGA (lowest 10 percent), risk was elevated for neonatal mortality (RR = 1.83, 95% CI: 1.34–2.5) and for post-neonatal mortality (RR = 1.9, 95% CI: 1.32–2.73). Christian and colleagues[19] have done pooled analysis from birth cohorts across low and middle-income countries, assessing the contribution of SGA and PTB to longer-term nutritional status outcomes. In their review—which included 19 birth cohorts across Asia, Africa and LAC (N = 44,374)–anthropometric measures at last contact, aged 12–60 months, were determined. Risk of child stunting was elevated for SGA (RR = 2.43, 95% CI: 2.22–2.66) as well as for PTB (RR = 1.93, 95% CI: 1.71–2.18). The population-attributable risk of SGA for child stunting was determined to be 20%, and for wasting, 30%. In a subsequent analysis[20] restricted to datasets for which anthropometric measures were available specifically at 24 months of age (14 cohorts, N = 18,061) the investigators found an elevated risk of stunting associated with SGA (OR 1.94, 95% CI: 1.59–2.36). In a global review of the contribution of various nutrition-related problems to under-5 mortality, Black and colleagues[21] found that 3.3% of under-5 deaths were attributable to IUGR/low birth weight.

### Evidence-Grounded Content; Contextually-Appropriate Delivery Strategy

This review consists primarily of a review of evidence for effectiveness of simple interventions that may contribute to better health outcomes. Scope is limited to interventions that can be delivered at the most peripheral level, by health auxiliaries and without access to lab support, approaches that lend themselves to closing the equity gap. No specific service delivery strategy is proposed. Depending on the setting, such interventions can be delivered through conventional facility-based ANC, outreach services or community health workers. They can be delivered on an integrated basis or through more specialized delivery channels.

## Methods

The scope for selection of interventions considered in this review included: 1) simple clinical preventive services (both universal preventive and screening) and 2) behavioral interventions. It was restricted to include only those program elements that can be delivered at the lowest level in the health system in low- resource settings. So for the interventions to be retained they needed to be simple enough that they would not require lab support of a kind that is frequently unavailable at—say—the “health post” level, although several interventions were retained which entail use of simple point-of-use diagnostics or subsequent referral to higher levels.

Since it was an important objective of this review to determine the *current state of evidence* on the effectiveness of these interventions, we did not restrict to experimental or quasi-experimental studies but, instead, drew on a broader range of potentially informative evidence particularly for those interventions for which RCT evidence is not available. Study methodology and quality were addressed in determining what inferences can be drawn from the available evidence but were not used as a basis for inclusion/ exclusion decisions. As a matter of principle, we understand “effect size” as measured in the real world to be a function of both the intervention and of characteristics of the study population; we were not interested in determining a pooled effect size for our retained interventions.

Detailed description of methods used are found in the annex, including S1 Note and S1 File.

## Results

The majority of the interventions identified as meeting our criteria are related to *nutrition* or *infection*. Most of the remaining interventions can be categorized as *obstetrical*. Many of the interventions can be considered *clinical*, either consisting of screening tests (e.g. HIV) or clinical preventive services (e.g. iron-folic acid supplementation). Another important subset of interventions can be characterized as *behavioral*. A small subset of interventions included are put into action only *after birth* (e.g. counseling on the importance of early initiation of breast-feeding). The rationale for including them is that in settings where a large proportion of births still happen at home, ensuring these interventions are used requires effectively reaching women while they are still pregnant.

### Nutrition

Burden of disease attributable to micronutrient deficiencies in pregnancy has recently been documented by Darnton-Hill and Mkparu[22]. A whole supplement of the Journal of Nutrition in 2003[23] was devoted to a review of nutrition and related factors in pregnancy. A more recent journal supplement [24] on nutrition in pregnancy included separate papers with systematic reviews of a range of interventions. Several multi- intervention systematic reviews have been done on nutritional interventions, including those delivered during pregnancy[25–29].

#### Breastfeeding

The primary interest for this review is ***early initiation*** and exclusive breastfeeding over the first hours and days of life—since these practices can, in principle, be influenced by effective counseling/ health education delivered to *pregnant women*. For breastfeeding practices later on—important though they are—other potential service delivery or health education opportunities *post-delivery* will tend to be more suitable as a focus for program strategy. Our particular interest is the expected benefits of counseling or health education during pregnancy on breastfeeding practices from shortly after birth through the first hours and days of life, particularly for births taking place at home. Influencing such practices for institutional deliveries relies more on hospital policies and practices and, as such, does not fall within the remit of this paper.

Breastfeeding significantly affects child growth and decreases the risk of life-threatening morbidities amongst infants and children[30–32]. WHO recommends exclusive breastfeeding for the first 6 months of life[32] but, as of 2012, only 39% of infants 0–5 months in developing countries were exclusively breastfed[33]. Rates of reported *early initiation* of breastfeeding (within the first hour after birth) are low in most low- and middle-income countries: 17% in Eastern Europe and Central Asia, 33% in Asia-Pacific, with slightly higher rates—near 50%—in Latin America and East and North Africa[34]. In India, the overall rate of initiation of breastfeeding within 1 hour of birth is 16% [33]. Delayed initiation of breastfeeding (beyond one hour of birth) is particularly prevalent in South Asia and West and Central Africa. It is also in those regions where there is the greatest discrepancy between richest and poorest wealth quintiles for this practice, to the disadvantage of the poorest[35].

In this instance, we are interested in 2 bodies of evidence:

1. impact of these practices on newborn survival; and
2. effects of counseling/ health education on timing of initiation and exclusivity of breastfeeding, particularly through program contact during pregnancy.

**Mortality Impact of Early Initiation and Exclusive Breastfeeding over the First Month:** A recent systematic review of initiation of breastfeeding *within first 24 hours after birth*[36] found 3 relevant, high-quality, observational studies: Mullany[37] (Nepal, N = 22,838), Edmond[38] (Ghana, N = 10,947), and Garcia[39] (India, N = 10,464). The review found, among mother-infant pairs initiating breastfeeding early, significantly lower all-cause neonatal mortality (RR = 0.56, 95% CI: 0.40–0.79) and infection-specific neonatal mortality (RR = 0.55, 95% CI: 0.36–0.84). These effect sizes were calculated adjusting for possible confounders, however—as observational studies—unmeasured and uncontrolled confounding cannot be ruled out. Two of the 3 studies[37, 38] compared survival between those initiating breastfeeding *within the first hour* vs. at later points in time. They found similar results with up to a doubling in mortality risk among those delaying initiation to more than 1 hour but less than 24 hours after birth. For initiation between 24 and 48 hours, risk was increased almost 3-fold compared to initiation within an hour of birth.

A more recent systematic review[40] which retained the same 3 studies referred to above on the mortality effects of early initiation[37–39] also found that neonates initiating breastfeeding beyond the first hour of life were at double the risk of newborn death of those first breastfed within the first hour (pooled OR 2.02, 95% CI: 1.40–2.93). This review also considered studies on the impact of *exclusive* breastfeeding over the first month of life on all-cause[38] and infection-specific mortality[41, 42], finding effects of similar magnitude to those for early initiation. They calculated that neonates not exclusively breastfed have almost 4 times higher risk of infection-related mortality than exclusively breastfed neonates (pooled OR 3.81, 95%CI: 2.19–6.64).

In an ecological study based on data from 67 Demographic and Health Surveys[43], a moderate negative correlation was found between neonatal mortality and initiation of breastfeeding reported within an hour of birth in countries with high neonatal mortality rate (NMR) (r = -0.327 in countries with NMR>29/1000). When adjusted for confounding, the authors found that countries in the lowest breastfeeding tercile had 24% higher NMR than those in the highest tercile (RR = 1.24, 95% CI: 1.07–1.44).

**Behavioral Effects of Breastfeeding Counseling/ Health Education during Pregnancy:** While our focus is on counseling and education during the *antenatal* period, we have retained reviews and studies using interventions that also included postpartum contacts. In general, we focus on evidence from low-income countries. There are 5 Cochrane reviews of some relevance[9, 44–47]. In the Cochrane review by Dyson et al.[44] only one non-US-based study was retained[48], a study in Nicaragua assessing the impact of early contact and rooming-in policies on breastfeeding initiation. The Lumbiganon review[45] restricted its assessment to studies of *antenatal* breastfeeding education. Timing of initiation was not assessed. The 16 RCTs retained for the Lumbiganon review were all were from high income countries.

Lewin[46] considers evidence on the effectiveness of lay health workers for various maternal and child health functions, including breastfeeding-related counseling and health education. In the Lewin review there were 3 retained cluster randomized controlled trials of lay health worker breastfeeding promotion, all conducted in Bangladesh:

- Haider[49, 50]: N = 726. Two home visits by peer counselors in the last trimester (and 12 more after birth). Initiation within the 1st hour was much higher in the intervention arm (64%) than among controls (15%) (p < .0001). Similarly, prelacteal feeds were much lower in the intervention arm (31%) than among controls (89%) (p < .0001). Exclusive breastfeeding over first 4 days was also much higher among those in the intervention (56%) than in the control arm (3%) (p < .0001).
- Baqui[51]: N = 46,444. Breastfeeding (including early initiation and exclusivity) was included in the content of both “home care” (which included 2 antenatal home visits) and “community care” arms, both of which demonstrated a significant improvement from baseline in the proportion initiating within one hour of birth (homecare: 45% → 81%, community care: 47% → 71%) vs. a smaller increase in the non-intervention comparison arm of 49% → 57%.
- Sloan[52]: N = 4,165. Community workers provided counseling to pregnant and postnatal women. Under the rubric of “community kangaroo mother care,” women were counseled on exclusive breastfeeding and early initiation, holding their newborns skin-to-skin 24 hours/ day and avoiding bathing the baby over the first few days of life. Breastfeeding within the 1^st^ hour was modestly higher in the intervention arm (52%) than among controls (41%) (p < .001). The proportion giving something other than breastmilk over the first few days of life was 90% among intervention, 96% among controls.

In a Cochrane review of community-based packages addressing maternal-newborn outcomes, Lassi et al. [9] found an increased likelihood of breastfeeding within 1 hour of birth (average RR = 1.94, 95% CI: 1.56–2.42, across 6 retained studies of community-based services in south Asia).

Renfrew et al.[47] restricts its focus to educational interventions and excludes those focusing only on the antenatal period. It included 52 studies, mostly in middle and high income countries; 4 from low or middle- low income countries, 3 of which were not included in the reviews already discussed (one conducted in Burkina Faso and Uganda[53]; another from India[54]; and another in Ghana[55]), but only one of which had findings relevant to our review, the Bhandari study[54]. It was conducted in Haryana, N = 895. Training was given to multiple cadres of existing workers, and interventions delivered took advantage of existing contacts, including community meetings. The focus was primarily on exclusive breastfeeding, but it also included early initiation and desisting from prelacteal feeds. The intervention used various print materials, including an antenatal handout. Improvements were seen, notably: initiation within 3 hours– 50% in intervention, 24% in controls, p < .0001; prelacteal feeds– 31% in intervention, 75% in controls, p < .0001.

**Other reviews:** In an early review, Green [56] found 51 studies on interventions aiming to improve breastfeeding. However, almost all focused on women giving birth in hospitals. Imdad’s review of individual or group breastfeeding counseling (both during and after pregnancy)[30] is used for a Lives Saved Tool (LiST) effect estimate. Outcomes included breast-feeding status at 4–6 weeks and beyond (exclusive, partial). Restricting to studies in LMICs, there was 89% higher likelihood of exclusive breastfeeding in intervention groups. Interventions including prenatal counseling had greater impact than those restricted to postnatal contact. Note that LiST models the effects of *exclusive* breastfeeding on diarrhea and pneumonia-specific mortality in the neonatal period (and beyond). It also models effects on stunting, mediated through diarrheal incidence. LiST does not specifically address the effects of *early initiation*.

The Imdad review is updated in Haroon [32]–“interventions included were those that offered education and/or support given to mothers through counselors (lay counselors and health professionals), and in either individual or group sessions, or a combination of both.” The review was not restricted to the antenatal period. Note that the review does not look in any detail at the characteristics of the promotional/ health education interventions. Haroon found, as a result of breastfeeding promotion interventions, significantly higher rates of exclusive breastfeeding within the first 24 hours (RR = 1.43, 95% CI: 1.09–1.87) and in the first month (RR = 1.30, 95% CI: 1.19–1.42). Educational interventions decreased rates of no breastfeeding by 32% at day 1 (RR = 0.68, 95% CI: 0.54–0.87) and by 30% in the first month (RR = 0.70, 95% CI: 0.62–0.80).

**Other individual studies:** Quinn[57] documents significant shifts in early initiation and exclusive breastfeeding in program efforts operating at scale (in catchment populations in the millions), building on other program platforms, using individual, group and mass-media approaches, implemented under multi-agency partnerships (in Bolivia, Ghana and Madagascar). The approach used focused on: formative research, targeted and consistent messages and materials, saturation of primary audiences, reaching pregnant and lactating women and those linked with them (husbands, grandmothers), training focused on counseling/ negotiation for health workers and community health workers (CHWs), and peer group support. Drawing on the same experience in Bolivia and Madagascar, Baker [58] reports that “in Bolivia [catchment population 1 million], timely initiation of breastfeeding went from 56% in 2000 to 69% in 2001 and reached 74% by the end of 2003. In Madagascar [catchment population 6 million], the [early] initiation rate went from 34% at baseline in 2000 to 69% in 2001, 76% in 2002, and rose to 78% in 2004.” This was achieved using an approach that emphasized: broad partner and stakeholder engagement, flexible and contextualized scale-up strategies, and saturation marketing approaches.

In a quasi-experimental study conducted in Cambodia[59], pregnant women were counseled on breastfeeding during home visits by community volunteers (Buddhist nuns and “wat grannies”). Over the intervention period, altogether 9,259 pregnant women were reached. At follow-up, women in intervention communities were 62% more likely to initiate breastfeeding within an hour of birth than those in control communities (RR = 1.62, CI: 1.30–2.01)

In a randomized controlled trial in an urban setting in Bangladesh that tested a 4-session antenatal nutrition group counseling intervention[60], those in the intervention arm were more than twice as likely as controls to initiate breastfeeding within an hour of birth (75% vs. 35%, p = 0.001). In a recent Pakistani cluster RCT[61] a cognitive-behavioral approach was used in counseling on breastfeeding, during pregnancy and over the first 6 months of life. Counseling was provided by Lady Health Workers during home visits. Although the intervention resulted in less use of prelacteal feeds in the intervention group (RR = 0.51, 95% CI: 0.33–0.78), timing of initiation was no better than among controls (RR = 1.07, 95% CI: 0.85–1.29).

**Conclusion:** Timing of initiation of breastfeeding appears to be an important remediable factor in newborn mortality risk, particularly in populations with high background mortality rates. Based on results of a study in southern Nepal, it is estimated that in such populations approximately 8% of all neonatal deaths could be avoided by increasing to universal coverage of initiation of breastfeeding within the first day of life; a possible *19%* if initiated within the first hour of life [37]. Note that the expected impact would vary considerably across populations, recalling that delayed initiation is especially prevalent in South Asia and in western and central Sub-Saharan Africa. Desisting from giving pre-lacteal feeds and continuing exclusive breastfeeding through the first days and weeks of life can be expected to confer further reduction in mortality risk.

There have been numerous well-documented experiences with educating and supporting women to adopt optimal breastfeeding practices. Some of these efforts have focused on women late in pregnancy with the goal of influencing timely initiation and continued exclusive breastfeeding over the first hours and days of life. The most appropriate strategies will depend on context, but efforts that successfully support such practices can make an important contribution to reducing risk of newborn death, particularly in disadvantaged populations with high neonatal mortality.

#### Iron/ Anemia

Iron deficiency is the most common nutritional deficiency in the world, with approximately 25% of women and children affected[62]. Anemia is also highly prevalent, with varying proportions attributable to iron deficiency depending on the epidemiologic setting[63]. Current best estimates are that 38% of pregnant women worldwide are anemic with the majority attributable to iron deficiency[63]. Anemia and/or iron deficiency in pregnancy is associated with a number of adverse outcomes for the mother and infant including increased risks for maternal mortality[64–66], preterm birth[67], infant mortality[66, 68] and small-for- gestational-age[69]. Given the challenges to increasing dietary sources of bioavailable iron, especially in low and middle income countries, antenatal iron-folic acid supplementation has been a mainline strategy used around the world to address anemia and iron deficiency in pregnancy. Two approaches to iron-deficiency anemia in pregnancy will be addressed in this review, universal supplementation, and test and treat.

Current guidelines in the industrialized world vary in their recommendations on the choice of these strategies. The US Centers for Disease Control and Prevention[70] and the WHO[71] recommend universal antenatal supplementation whereas the American Academy of Family Physicians[72], the American College of Obstetricians and Gynecologists[73] and the Institute of Medicine of the US National Academy of Sciences[74] all support a test and treat approach. Recently, a systematic review was conducted to update the US Preventive Services Task Force recommendations on this topic[75]. They concluded that no new evidence was available to update their current recommendations. Thus, evidence from the industrialized countries where iron deficiency is much less prevalent or severe offers little guidance on how low and middle-income countries should proceed with the choice between these 2 approaches.

**Universal Antenatal Iron Supplementation:** The most recent Cochrane review in 2015 summarized 44 trials with over 43,000 women enrolled. However, the majority of trials included were conducted in the industrialized world with only 6 done in LMICs in Asia plus an additional 5 done in China, 4 in sub-Saharan Africa, and 2 in the Latin America-Caribbean region. Outcomes evaluated included maternal anemia at term, iron deficiency anemia at term, iron deficiency at term, birthweight, preterm birth, neonatal mortality, and placental malaria infection[76]. Almost all outcomes showed either significant heterogeneity in results and relatively low to moderate quality evidence according to the GRADE criteria. They report significant reductions in anemia, iron deficiency anemia and iron deficiency at term, of 70%, 67%, and 57% respectively, and all confidence intervals excluded no difference. These results on hematologic outcomes are consistent with a previous systematic review[77].

Observational data and underlying biological mechanisms support an elevated risk of maternal mortality among anemic women in LMICs where blood transfusions are difficult to access and other aspects of management of post-partum hemorrhage are often of poor quality. There is an approximate 30% (95% CI: 15%–40%) lower risk of maternal death per every 10g/dL higher hemoglobin level in pregnancy[65]. RCT data support this observation but the strength of evidence in low due to the very small number of maternal deaths even when pooled across studies. The effect size for universal iron supplementation in pregnancy was RR = 0.33 (95% CI: 0.01–8.19)[76].

Infant outcomes showed modest levels of evidence in support of lower risk among those randomized to receive iron supplementation during pregnancy. In the Cochrane systematic review, among infants whose mothers received iron supplementation during pregnancy the proportion born low birthweight (<2500 g) was 8.4% versus 10.3% among controls (RR = 0.84, 95% CI: 0.69–1.03)[76]. It is important to note the low proportion born with low birthweight in the control group rate (10.3%). Rates among populations in south Asia for example would be considerably higher and this likely explains the heterogeneity in results. For example, one study from Nepal showed a similar 16% relative reduction, but reduced low birthweight from 43% in the control group to 34% in the iron-supplemented group (95% CI: 0.72–0.99)[78]. Preterm rates were also lower in the iron supplemented group, but not significantly so in the systematic review (RR = 0.93, 95% CI: 0.84–1.03)[76]. Neonatal mortality rates were lower as well in the supplemented group (RR = 0.91, 95% CI: 0.71–1.18) but, again, the level of evidence was low. Interestingly, when the Nepal group was followed through 7 years of age, the iron supplemented group showed a lower mortality than those in the control group. The relative risk of mortality by 7 years was 0.69 (95% CI: 0.49–0.99)[79, 80].

One very important subgroup of studies is those conducted in areas with endemic malaria. Studies of *childhood* supplementation have suggested that iron supplementation may be hazardous in settings where malaria is not well controlled[81, 82]. The Cochrane systematic review showed no support for an increased risk of *placental* malaria infection[76]. More recently, a trial conducted in Tanzania in pregnant women without anemia and who were iron-replete also showed no adverse impact of iron supplementation in a malaria-endemic setting where good malaria control was practiced[83]. Positive impacts were seen on hematologic outcomes (iron deficiency and iron deficiency anemia) but no impact was observed on birthweight or on placental malaria[83].

**Conclusion:** Universal antenatal iron supplementation (typically combined with folic acid) is safe, even in areas with endemic malaria when malaria control efforts are well implemented, and shows high efficacy for maternal iron deficiency and maternal iron deficiency anemia. Evidence for benefit for other maternal outcomes such as maternal mortality or serious morbidity is weak, but estimates of relative risk are below 1.00 (beneficial) even in studies with limited numbers of maternal deaths. The evidence for benefit to infants from antenatal iron supplementation is also limited but the direction of effects is towards the beneficial and in populations with high rates of iron deficiency anemia and high rates of adverse pregnancy outcomes, there is modest evidence of impact on low birthweight and childhood mortality.

**Test and Treat Antenatal Iron:** Testing for anemia and then treating with iron is an uncommon strategy in most LMICs although it is recommended by many professional groups in industrialized countries. The most recent Cochrane review was done in 2011 but it must be recognized that much of the literature on universal antenatal iron supplementation in fact includes many women who would be eligible under the test and treat approach depending on the rates of anemia in the study populations[84]. The review included less than 3200 women from 23 randomized trials; these studies used a wide range of doses and formulations for both oral and parenteral iron treatment. As a result, conclusions that can be drawn regarding clinical outcomes in both the women and their infants are extremely limited and no consistent differences were noted. The variability in treatment protocols across studies makes it very difficult to come to any solid conclusions. There is good evidence that oral iron treatment improved hematologic outcomes (maternal iron deficiency anemia and maternal anemia) (RR = 0.38, 95% CI: 0.26–0.55) but is based on only one trial, and that parenteral iron (either intramuscular or intravenous) improved these indicators slightly better than oral preparations alone. There was also weak evidence that lower oral doses produced low rates of side-effects such as gastrointestinal symptoms. A more recent trial conducted in Mississippi demonstrated that while a systematic test-and-treat approach was better than test-and treat-among only high risk women, universal supplementation produced better hematologic outcomes than either of the other 2 groups[85]. Women in the control group (high risk pregnancy test-and-treat) had a rate of anemia at 6 months post-partum of 34% versus 43% in the systematic test-and-treat group. The universal supplementation group had a rate of only 22.5% (p<0.001)[85].

**Conclusion:** Test-and-treat strategies may be appropriate for settings where underlying rates of iron deficiency anemia are low and it is likely that selection of women who are anemic in early pregnancy will emphasize the beneficial effect of iron supplementation on hematologic outcomes. There is almost no evidence that clinical outcomes are affected in either direction. The most recent trial from the US suggests this issue is relatively unimportant as universal supplementation seems to improve hematologic outcomes better than test-and-treat and side effects can be limited with the use of lower dose oral preparations.

**Overall Conclusion:** The available data suggest that universal iron supplementation (most commonly including folic acid) can achieve significant improvements in maternal iron deficiency and maternal iron deficiency anemia in LMICs. There is little evidence that a test-and-treat approach would be better from either an outcomes or a cost- effectiveness perspective. The issue of compliance is often raised by those concerned about side effects of oral iron, but program experience and limited evidence available from the literature suggest that in fact women are compliant when supplies are consistent and they receive supportive messages [86]. While the data are very limited, it may be beneficial to combine universal antenatal iron supplementation with deworming treatment especially in settings hookworm infection is prevalent[71].

#### Multi-Micronutrient Supplementation

Various micronutrients have been investigated for their role in pregnancy outcomes (iron, folic acid, calcium, magnesium, zinc, vitamin A/ B-carotene, vitamin D3, iodine, omega-3 fatty acids, and others). A programmatically pragmatic approach has been the use of combined multi-micronutrient supplements. As discussed in the last section, there are unambiguous benefits to iron supplementation. Therefore, trials of micronutrient supplementation have normally used iron-folic acid supplementation as the comparator. It is important to note that the micronutrient composition of the supplements used varies across studies.

**Evidence:** Haider and Bhutta’s Cochrane review[87], using iron-folic acid as comparator, found 11% lower risk of low birthweight (RR = 0.89, 95% CI: 0.83–0.90) and a similar reduction in risk of small for gestational age (RR = 0.87, 95% CI: 0.81–0.95) but no other effects on pregnancy outcomes, including neonatal mortality (RR = 1.01, 95% CI: 0.89–1.15). The same author group did further analysis[88] to be used as a basis for LiST tool effect size. This included disaggregated analysis by settings with institutional delivery rates above or below 60%, finding higher risk of neonatal death among those receiving multi-micronutrient supplementation in trials with lower institutionalized delivery rates (RR = 1.47, 95% CI: 1.13–1.92) but no elevated risk in trials conducted in settings with higher institutionalized delivery rates (RR = 0.94, 95% CI: 0.81–1.09).

Ramakrishnan’s slightly more recent review[89], which included a similar but not identical set of trials to the Cochrane review, also found reduced risk of *low birthweight* (RR = 0.86, 95% CI: 0.81–0.92, from 15 trials), and SGA (RR = 0.83, 95% CI: 0.73–0.95, from 8 trials). No effect was found for *stillbirths* (RR = 1.00, 95% CI: 0.84–1.21, 10 studies) or *neonatal deaths* (RR = 1.07, 95% CI: 0.87–1.32, 9 studies). Ramakrishnan [90] included the same analysis as the review just mentioned[89], but also did sub-group analysis of neonatal deaths in the 5 trials that began supplementation beyond the first trimester and found *elevated risk* (RR = 1.38, 95% CI: 1.05–1.81).

The largest trial to date[91], conducted in Indonesia, did show a reduction in early infant deaths, i.e. through 90 days of age (RR = 0.82, 95% CI: 0.70–0.95). Christian et al.[78], based on work done in Nepal, documented that the excess deaths in their study were restricted to term newborns and that those receiving supplementation were at higher risk of birth asphyxia (2008). The Nepal study[92, 93] also found that although mean birthweight was greater in the supplementation arm, this was a result of a shift of the whole birthweight distribution. By contrast, iron and folic acid supplementation reduced those at the low end of the weight distribution but did not affect the upper tail of the distribution.

**Conclusion:** Most trials have shown reduced risk of low birthweight, in the range of 10–15%. However, studies have *not* shown benefit with regard to risk of stillbirth and there is some suggestion that risk of neonatal death may be *increased* under some circumstances. The evidence for this increased risk has been shown only in settings where the prevalence of maternal stunting is very high. Evidence available suggests that the marginal effect of multi-micronutrient supplementation over iron-folic acid is greater fetal weight-gain across the birthweight distribution. Reductions in mortality risk due to fewer babies born low birthweight may be more than offset, at least in some settings, by increased mortality risk at the top end of the birthweight distribution.

#### Calcium

Along with post-partum hemorrhage, eclampsia/pre-eclampsia is the most important direct cause of maternal deaths worldwide. This condition also significantly increases risk to the newborn (sf. section on pre- eclampsia screening and management). Decades ago, it had been observed that pre-eclampsia rates are higher in populations with lower calcium intake[94]. Since then, calcium supplementation has been tested for efficacy in reducing the burden of poor outcomes associated with pre-eclampsia.

We focus our review on studies in settings with low calcium intake in which supplementation is offered to all pregnant women, not restricting to those at higher risk for pre-eclampsia. With our attention on mortality endpoints and intermediate conditions with a strong association with mortality risk, we have not retained studies that only look at pre-eclampsia as an outcome (i.e. which do not also look at severe outcomes).

**Maternal Death or Serious Morbidity:** The main endpoints in most calcium trials to date have been maternal, with many also considering risk of pre-term birth. According to the most recent Cochrane review[95] the risk of pre-eclampsia is reduced by 55% with supplementation (RR = 0.45, 95% CI: 0.31–0.65). However, it cannot be assumed that there is necessarily a reduction of the same magnitude in associated serious outcomes.

Although severe maternal outcomes were of greatest interest, because they are comparatively rare events the trials were generally underpowered to detect such an effect. This problem was addressed in the large multi- country WHO trial [96] (N = 8,325) with populations known to have low baseline calcium intake, by creating a composite score of *maternal death or serious morbidity* which included all maternal deaths and several other endpoints, some specific to pre-eclampsia (e.g. HELLP syndrome), some not (e.g. death and ICU admission). By this measure, risk was reduced (RR = 0.80, 95% CI: 0.60–0.97). The main driver of this composite endpoint was ICU or special-care unit admission for *any* cause, for which risk was reduced by 16%. These admissions accounted for over 2/3s of the events making up the composite score. Severe pre- eclampsia-specific morbidities not requiring ICU admission were reduced by 23%.

Note that the Villar study[96] is by far the largest calcium supplementation trial and represents the main evidence available on its effects on the endpoints of interest for this review. The study was conducted in 6 countries. Using World Bank categories, 3 are currently considered lower middle income (India, Egypt, Vietnam) and 3 upper middle income (Argentina, Peru, South Africa). The overall maternal mortality ratio among those enrolled in this trial was 84/100,000.

The Hofmeyr Cochrane review[95], retained 4 trials (N = 9,732) using high-dose calcium for the outcome maternal death or serious morbidity, including the Villar study[96], and came up with the same effect size (RR = 0.80, 95% CI: 0.65–0.97).

**Preterm Birth:** In the Villar study[96], risk of *overall* preterm birth was not significantly lower (RR = 0.91, 95% CI: 0.79–1.05) although risk of *early* preterm birth (<32 weeks of gestation) was lower in the intervention arm than in controls (RR = 0.82, 95% CI: 0.71–0.93). The Hofmeyr meta-analysis[95] (11 trials, restricted to those using a dose of 1000mg or higher, 15,275 women) found a lower risk of preterm birth with supplementation (RR = 0.76, 95% CI: 0.60–0.97). Buppasiri’s Cochrane review, updated in 2014[97] added one more trial to those reviewed by Hofmeyr for high dose supplementation and found a similar effect size (RR = 0.81, 95% CI: 0.66–0.99). An earlier review[98], restricted to studies from low and middle income countries, also showed lower risk of preterm birth.

**Other Outcomes:** Villar[96] is the one published paper to report on all-cause *neonatal mortality* as an outcome, finding RR = 0.70 (95% CI: 0.56–0.88). Trials have generally also shown an effect on incidence of *low birthweight*, although this appears to be largely accounted for by the effect on risk of preterm birth rather than on fetal growth. Evidence to date does not suggest a benefit with regard to stillbirth.

**Conclusion:** Effect sizes used in the LiST tool are 0.80 (95% CI: 0.70–0.91) for *severe maternal morbidity/ mortality* attributable to eclampsia/ pre-eclampsia[99] and 0.70 (95%CI: 0.56–0.88) for all-cause *newborn mortality* [98]. As discussed above, the effect size for maternal outcomes in the Villar study was calculated based on *all* maternal deaths and ICU admissions as well as pre-eclampsia-specific severe outcomes, so applying the measured effect size only to the portion of maternal deaths attributed to eclampsia/ pre-eclampsia—as done in LiST—may under-estimate its preventive efficacy. Based on data reported in the Villar trial[96], it appears that a reduction of pre-eclampsia-attributable serious maternal events (including death) of 30–50% could be expected.

In populations with low calcium intake, there is credible evidence of a reduction in risk of maternal death associated with eclampsia/ pre-eclampsia, although evidence available does not permit a confident quantification of benefit. Calcium supplementation appears to reduce the risk of *preterm birth* by around 15–20%. There is no compelling evidence for reduced risk of stillbirth or intrauterine growth restriction (although pre-eclampsia is known to increase risk of intrauterine growth restriction). The Villar study found a 30% lower risk of all-cause neonatal mortality in the intervention arm. This would make calcium supplementation among the most potent interventions for reducing the burden of such deaths, in calcium deficient populations.

The current WHO recommendation for pregnant women in populations with low calcium intake is to use a daily supplement with a dose of 1.5–2.0 gm of elemental calcium (i.e. 3–4 tablets). In the form of calcium carbonate, a 20-week supply would weigh close to 1kg. The weight, bulk and cost pose significant logistical challenges in low income settings where there could be significant health benefits.

In Hofmeyr’s systematic review[100] of supplementation using lower dose calcium (<1gm/ day), risk of pre- eclampsia was reduced (RR = 0.38, 95% CI: 0.28–0.52). At such a dose, logistical challenges would be less imposing. However further dose-finding trials are required to determine if calcium at lower doses can reduce risks for more severe endpoints.

#### Antenatal Vitamin A

Close to 20 million pregnant women annually are estimated to be vitamin A deficient (VAD) to some degree, most in South and Southeast Asia[101]. Yet the ocular manifestations of vitamin A deficiency during pregnancy are rare except for night blindness which typically occurs late in pregnancy and is indicative of depleted liver reserves. Pregnant women who are vitamin A deficient and their infants can suffer significant adverse consequences as a result of this deficiency. As with all supplementation strategies to prevent the complications associated with micronutrient deficiencies, the impact of supplementation in the antenatal period will depend on the overall vitamin A status of the population and the special demands that pregnancy makes on vitamin A reserves. As a result, it should be expected that effect size estimates of supplementation on outcomes will vary based on these parameters and that a common effect size is unlikely. In fact, the current evidence provides some support for this related to vitamin A supplementation during pregnancy. One additional issue for vitamin A supplementation in pregnancy is that high doses of retinol are a well-known teratogen if provided early in gestation[102]. That said, there is also evidence that vitamin A deficiency interferes with proper fetal development[103]. As a result, any routine antenatal supplementation with vitamin A is typically provided on a regular (daily or weekly) basis that is limited to the current recommended daily allowances (770 micrograms daily or 2,600 IU)[104].

**Evidence:** The first cluster randomized RCT was done in a population in rural, southern Nepal with a high prevalence of low vitamin A-intake and poor vitamin A status[105]. This trial randomized 44,646 women of reproductive age to a universal supplementation strategy with weekly dosing of retinol vs. β-carotene vs. placebo over a 3½ year period. 22,189 pregnancies occurred in the 3 study groups. Maternal mortality was the primary endpoint. The study found a RR of 0.60 (95% CI: 0.37–0.97) and 0.51 (95% CI: 0.30–0.86) for retinol and beta-carotene, respectively, versus placebo [105]. Two additional RCTs were conducted in Ghana and Bangladesh to confirm (or refute) the findings from Nepal. Neither found any significant benefit on maternal mortality. The trial in Ghana compared 25000 IU (weekly) of retinol against placebo among 207,781 women aged 15–45 in rural, central Ghana, an area without significant vitamin A deficiency among women[106]. A total of 78,835 pregnancies comprised the primary analysis. The adjusted odds ratio was 0.92 (95% CI: 0.73–1.17)[106]. The trial in Bangladesh included 59,666 pregnancies randomized to vitamin A (7,000 μg), β-carotene (42 mg), or placebo from the first trimester through 12 weeks post-partum[107]. This population had evidence of significant vitamin A deficiency, with night blindness rates during pregnancy approaching 10%. Relative risks for total mortality in the vitamin A and beta carotene groups were 1.15 (95% CI: 0.75–1.76) and 1.21 (95% CI: 0.81–1.81), respectively versus placebo[107]. In summary, there is conflicting evidence for benefit of vitamin A or β-carotene supplementation on maternal mortality. Meta-analyses do not support this intervention as a maternal survival strategy[101, 108] however the most recent such analysis noted significant heterogeneity in trial outcomes (I2 = 52%, p = 0.04) most likely due to variations in underlying population levels of vitamin A deficiency between studies[101].

In addition to the maternal mortality outcomes, trials also reported on infant survival, stillbirth, preterm birth, birthweight, anemia in pregnancy and the infant, and growth of children post-natally. There was no evidence that antenatal vitamin A supplementation reduced infant mortality, stillbirth, preterm birth or increased birthweight[19, 101, 107–110]. In one trial from Indonesia, there was a small increase in birth length among newborns whose mothers received antenatal vitamin A versus placebo or combined vitamin A plus zinc, but the difference was small (0.5 cm) and there was no difference in birthweight[110]. Overall, there is no consistent evidence that antenatal vitamin A supplementation improves growth of infants through the first year of life[101]. The evidence for reductions in anemia in pregnancy show a 19% reduction in anemia (hemoglobin <11 g/dl) and an approximate 0.2 g/dL increase in mean hemoglobin levels at the end of pregnancy[101]. However, there was little evidence for reduction in severe anemia in pregnancy[101].

**Population Subgroups:** Two subgroups have been explored, women with night blindness during pregnancy and those who are HIV positive.

**Night Blindness in Pregnancy:** A stratified analysis of the Nepal trial showed significantly higher infant mortality for children born to women who were night blind during pregnancy and that antenatal vitamin A supplementation attenuated this risk[111]. Increases in infant mortality for the placebo and β- carotene groups were 63% and 50% respectively but only 14% higher in the vitamin A group. These results supports the current recommendation of treating pregnant women with vitamin A who present with night blindness.

**Maternal HIV Infection:** There is no consistent evidence that antenatal vitamin A supplementation reduces risk of mother-to-child-transmission of HIV (MTCT) but there is significant heterogeneity in trial results [101]. One trial from Tanzania showed an *increased* risk of MTCT[112], but 2 others showed no difference[113, 114]. Pooled analysis of these 3 trials with 2,053 enrolled showed a significant reduction in risk of low birth weight (RR = 0.79, 95% CI: 0.64–0.99)[101]. No difference was observed among uninfected women[101].

**Conclusion:** There is questionable benefit but available evidence supports current recommendation for a screen and treat strategy for maternal night blindness during pregnancy. With the major reductions in maternal mortality that have occurred since the Nepal trial was done this may have largely eliminated the attributable portion of maternal deaths due to vitamin A deficiency.

#### Newborn Vitamin A Advance Distribution

As mentioned in the section on antenatal vitamin A supplementation, almost 20 million women are vitamin A deficient, with particularly high prevalence in South and Southeast Asia[101]. All infants are born with limited to modest liver reserves of vitamin A and depend on breastfeeding to provide this essential nutrient for proper growth and development[115]. Women who are vitamin A deficient have lower levels in breastmilk thus limiting the intake during this crucial age[115]. Vitamin A deficiency also increases in severity as infants wean off breastmilk and onto the nutritionally inadequate weaning diets common in low income countries. Multiple studies have demonstrated that vitamin A supplementation among children 6 months to 5 years of age in populations with endemic vitamin A deficiency can reduce mortality[116]. Despite this benefit, it was assumed for many years that children younger than 6 months and breastfeeding either exclusively or for the majority of their intake were protected by the vitamin A in breastmilk. Supporting this view, there was no evidence of benefit of vitamin A supplementation on mortality among children under 6 months[117] until Humphrey et al. published in 1996 a small trial from Indonesia demonstrating a dramatic reduction in mortality among infants dosed within the first day of life[118]. This finding was ignored for many years until the first of a set of randomized trials was published by Rahmathullah et al. from South India [119]. The positive results of this second trial set in motion a series of additional trials that have brought us to our the current state of evidence[120–125].

**Evidence:** The studies identified for this review found varying results of vitamin A supplementation on infant mortality. Findings from trials conducted in South and Southeast Asia have shown significant reductions in early infant mortality. In Indonesia, the RCT observed significant reduction in mortality (RR = 0.36, 95% CI: 0.16–0.87). This study was too small for adequately powered subgroup analysis[118]. Similar findings, although not as dramatic were also seen in the larger RCTs conducted in South India, Bangladesh, and North India. In South India, the impact was a 22% reduction in 6-month infant mortality (95% CI: 4%–37%)[119] and further analysis demonstrated this effect was accomplished through a reduction in case fatality for diarrhea (relative case fatality 0.50, 95% CI: 0.27–0.90) and fever (relative case fatality 0.60, 95% CI: 0.40–0.88)[126]. In Bangladesh, the investigators found showed a 15% reduction in all-cause mortality for supplemented infants (95% CI: 27%–0%)[121]. The most recent South Asian trial conducted in North India demonstrated a 10% reduction in mortality (95% CI: 19%–0%)[123].

In contrast to the South Asian results, all the RCTs conducted in sub-Saharan Africa have shown either no benefit or evidence of slight harm. The first RCT conducted in Zimbabwe was a 2x2 factorial trial with newborn and maternal post-partum supplementation. Among HIV negative women, hazard ratios for the groups who received both maternal and newborn vitamin A, maternal vitamin alone, and newborn supplementation alone relative to placebo for both were 1.28 (95% CI: 0.83–1.98), 1.27 (95% CI: 0.82–1.97), and 1.18 (0.76–1.83), respectively[120]. Among HIV positive women in Zimbabwe, there was no evidence for impact of vitamin A supplementation for mothers and/or their newborns on mortality[127]. A trial in Guinea-Bissau showed that vitamin A supplementation (25,000 IU at birth) was not significantly associated with mortality (RR = 1.08, 95% CI: 0.79–1.47)[122]. The 2 recent trials conducted in Ghana[124] and Tanzania[125] showed no evidence of benefit on 6-month infant mortality with RR = 1.12 (95% CI: 0.95–1.33) and RR = 1.10 (95% CI: 0.95–1.26) respectively.

A number of systematic reviews and meta-analyses have been conducted on this topic, but none have included the 3 recent large trials in North India, Ghana, and Tanzania and are thus out of date. Investigators representing all these trials have met at WHO under the coordination of the Child and Adolescent Health Group to conduct a pooled analysis of the data from all randomized trials. While those results are not yet published, they show a difference between the results of trials conducted in settings with significant maternal vitamin A deficiency (Indonesia, South India, Bangladesh, and North India) and those in settings with little to no maternal vitamin A deficiency (Zimbabwe, Guinea-Bissau, Ghana, and Tanzania). In settings with endemic vitamin A deficiency there is an overall benefit to newborn dosing with regard to early infant mortality whereas in settings without deficiency there is no evidence of benefit.

**Conclusion:** Pooling results across all RCTs shows no benefit from newborn vitamin A supplementation on early infant mortality. However, there is significant heterogeneity in results with benefit evident in settings with significant maternal vitamin A deficiency but no benefit when maternal vitamin A deficiency is lacking. Prepositioning a single capsule of vitamin A (50,000 IU) at the location of delivery in those settings where benefit can be expected (primarily poorer, South Asian populations) is feasible. The expected reduction in mortality burden achievable with this intervention will vary depending on the population burden of vitamin A deficiency among women of reproductive age.

#### Iodine

UNICEF estimates that globally, each year there are approximately 35 million births at risk of complications of iodine deficiency (over 2/3s of which are in Sub-Saharan Africa and South Asia), the most severe of which are cretinism, stillbirth and miscarriage[128]. Inadequate dietary intake of iodine during pregnancy remains the most important preventable cause of mental retardation worldwide. It is estimated that about 18 million infants suffer some significant degree of mental impairment each year[129].

Although, in principle, supplementation could be considered (either complementary to or as a substitute for salt iodization), in this review we are primarily concerned with efforts to ensure high coverage for use of iodized salt by pregnant women.

**Evidence:** Household use of iodized salt varies considerably between countries (as documented in UNICEF MICS, DHS and other surveys). According to the UNICEF global nutrition database[128], average household iodized salt use across recent surveys in sub-Saharan Africa was 59%. The proportion was slightly higher in south Asia (69%). There are many countries however with levels of use considerably lower than these averages. Wu’s Cochrane review[130] applied inclusion/ exclusion criteria such that it was not possible to compare effects of iodized salt use by pregnant women with no supplemental iodine source. Aburto’s recent systematic review for WHO[131] identified 89 relevant studies on the effects of salt iodization. Pooling results from 18 quasi-experimental studies with IQ as outcome, risk of low intelligence was markedly reduced (RR = 0.28, 95% CI: 0.22–0.36).

**Conclusion:** Unlike other interventions addressed in this review, salt iodization does not appear to confer significant protection for the mortality and risk conditions of interest to us. However, its effects on preventing long-term cognitive disability are considerable. Public health measures to achieve high coverage for use of iodized salt, particularly for pregnant women, are important for reducing the burden of preventable cognitive disability. In all countries, regulation and effective enforcement are needed to ensure that all salt produced for domestic consumption is iodized.

#### Balanced Protein-Energy Supplementation

Poor nutrition status before and during pregnancy has long been recognized as an important risk for poor pregnancy outcomes. This has prompted trials of protein-energy supplementation, with protein making up less than 25% of the energy content.

**Evidence:** In the Cochrane review by Ota et al.[132], 11 trials were identified which tested the benefits of balanced protein-energy supplementation (N = 5,385), most of which were conducted in high-income countries. This review included all the relevant trials from LMICs reviewed by Imdad[133] and Stevens[134] discussed below. The proportion born small for gestational age was reduced in the intervention arm (RR = 0.79, 95% CI: 0.69–0.90; 7 studies). Preterm rates did not differ between intervention and control. They found that risk of stillbirth was significantly reduced (RR = 0.62, 95% CI: 0.40–0.98). The pooled effect size for neonatal death was RR = 0.68 (95% CI: 0.43–1.07; 5 studies). Note that the mean neonatal mortality in the comparison arms of these trials was 26/1000. Also, they reported that “rather small net increases in energy intake [were] achieved in most of the trials.” The one exception was a trial conducted in the Gambia (Ceesay[135], N = 2047), which used a supplement with a much higher calorie content. It was also the largest of the trials captured in these reviews. The intervention consisted of supplement biscuits containing roasted groundnuts, rice flour, sugar, and groundnut oil (1017 kcal energy, 22 g protein, 56 g fat, 47 mg calcium, and 1.8 mg iron) consumed daily in the presence of birth attendants. Supplementation began at 20 weeks gestation. In this study, mean body mass index (BMI) post-delivery was 21.3 in intervention group and 20.7 in control. Pre-pregnancy BMI measures were not available, although the study was conducted in a rural setting where residents were reported as generally being “chronically undernourished.” In this study the risk of low birthweight was lower in the intervention arm (OR = 0.61, 95% CI: 0.47–0.79), as was risk of stillbirth (OR = 0.47, 95% CI: 0.23–0.99) and of death within the first week of life (OR = 0.54, 95% CI: 0.35–0.85).

The outcomes addressed in the review by Stevens[134] included birthweight, other newborn anthropometrics, and longer-term measures of infant growth. The main outcomes considered by Imdad[133] were small-for-gestational-age, mean birth weight and neonatal mortality. All of the trials captured were also included in the Cochrane review by Ota discussed above [132]. They found a reduced risk of small-for- gestational-age (RR = 0.69, 95% CI: 0.56–0.85; 6 studies)–which they recommend as the effect size to be used in the LiST tool—and a non-significant effect on neonatal mortality (RR = 0.63, 95% CI 0.37–1.06; 3 studies). Imdad modeled an effect size on small-for-gestational-age of 0.32, and a knock-on assumed effect on stunting for inclusion in the LiST tool.

Imdad[136] used the endpoint “low birthweight” and, not surprisingly, found results similar to those of their earlier review[133], which analyzed small-for-gestational-age (RR = 0.68, 95% CI: 0.51–0.92; 5 studies). They also found a reduced risk of stillbirth (RR = 0.62, 95% CI: 0.40–0.98). Effects were larger in undernourished women than in the adequately nourished.

**Conclusion:** Studies have been done in diverse populations with diverse nutrition status and diverse composition of the supplements. Furthermore, the trials have been comparatively small, with the largest having a sample size just over 2000. This makes interpretation of the available evidence challenging. It can be concluded however that balanced protein-energy food supplementation can significantly reduce the proportion of babies born small for gestational age. The poorer the nutrition status and greater the increase in caloric intake, the larger the expected benefit. The Cochrane review by Ota[132] found a 21% reduction in risk of SGA (7 studies). Retaining a slightly different set of studies, Imdad[133] found an effect size of 31%. Ota also found a 38% reduction in stillbirth. Across 5 studies, Ota calculated a pooled effect size for neonatal mortality of RR = 0.68 (95% CI: 0.43–1.07). A real reduction of 32% in neonatal mortality would, of course, be an important population health effect however with such a wide confidence interval this finding is entirely compatible with no mortality effect. Nevertheless, a significant reduction in small-for-gestational-age would be expected to reduce newborn mortality, as well as stunting in infancy and childhood[137]. Notably, in the largest trial to date[135] there was a significantly reduced risk of mortality through 7 days of life. Unaddressed in this literature is the potential for increased risk of obstructed labor among adolescent girls or women who are stunted (see comments in section on multiple micronutrient supplementation).

So, we can conclude that particularly for malnourished women, pregnancy outcomes (intrauterine growth restriction, stillbirth, and early newborn deaths) can be improved with balanced protein-energy supplementation. However, depending on the size and maturity of the mother, larger babies may result in excess risk of obstructed labor and its consequences. Also, effectively delivering such programs at large scale would be very challenging. Nahar[138] and Liberato[139] discuss practical implementation challenges.

#### Nutrition: Summary

As outlined in Table 2, nutrition-related interventions delivered during pregnancy have significant potential impact on the outcomes considered in this review. Although use of iodized salt does not contribute to the mortality outcomes, its widespread use has been the single most important factor in reducing the global burden of preventable cognitive disability. In settings where use is not yet universal, continued program effort is needed.

**Table 2:** Summary of Effects of Nutrition Interventions on Outcomes of Interest.

Intervention	Effects on:						Comments
	Maternal mortality	Newborn mortality	Stillbirth/ miscarriage	Preterm	IUGR/ LBWt	Other outcomes	
**Breastfeeding –early initiation**		Up to 20%↓ esp. in high mortality populations, evidence based on observational studies [37]					The effectiveness estimate assumes 100% adherence to this behavioral recommendation.
**Iron/ anemia**	↓OR 0.71 for 10g/dL lower Hgb; studies underpowered[65]	↓? studies underpowered	**/**	↓? studies underpowered	up to ~15%↓[78]	Longer term follow-up suggests ~30%↓ in mortality among children 3mo–7yr [80, 92]	No evidence for superiority of screen & treat over universal supplementation
**Multi-micronutrient supplementation**			**/**		~15%↓ [88]	Expected knock-on effects on stunting	
**Calcium**	30%↓ or more reduction in PE/E-specific mortality, in high mortality populations; RCT evidence, but underpowered	30%↓ in all-cause neonatal mortality in calcium-deficient populations, based mainly on WHO RCT [96]	**/**	20%↓ [97]		**/**	Effectiveness estimate assumes 100% coverage.
**Antenatal vitamin A**	**/**	**/**	**/**	**/**	**/**	When given to night-blind pregnant women, infant mortality is ↓d	
**Postnatal vitamin A**						10% or greater ↓ in infant mortality to 6 months of age in areas with endemic vitamin A deficiency [119, 121, 123]	
**Iodine**						↓ cognitive disability; universal coverage could prevent ~18M cases of cognitive impairment/ year [129]	
**Balanced protein-energy supplementation**		Up to ~30%↓?, RCT evidence underpowered [132,135]	Up to ~40%↓ in populations with significant malnutrition [132, 135]	**/**	up to 30%↓ in populations with significant malnutrition [133,135]	Expected knock-on effects on stunting	Effectiveness estimate assumes 100% coverage.

Notes on table: **/** indicates that this has been assessed and there is no evidence of an effect.

Early initiation of breast-feeding (i.e. within an hour of birth) generally receives relatively little program attention but it can be expected to significantly reduce newborn mortality in settings where mortality rates are still high. In calcium-deficient populations, antenatal supplementation can be expected to have large magnitude effects for pregnancy outcomes, but the currently recommended dosing imposes important logistical challenges.

### Infection

Several reviews have been published [140–143] that look at the evidence for the effectiveness of screening and other interventions related to infections during pregnancy. In this section we will consider interventions specifically addressing the following: helminths, malaria, tetanus, sepsis arising through cord-stump exposure, syphilis and HIV/AIDS. Other interventions were considered but not retained because they required lab services often not available at the most peripheral level in low income countries.

#### De-worming

Hookworm and other soil-transmitted helminths such as *Ascaris* and *Trichuris* are associated with iron- deficiency anemia (mainly due to blood loss) and other nutritional deficiencies. Helminth infections during pregnancy are very common, particularly in highly impoverished, rural populations with poor sanitation in sub-Saharan Africa, Southeast Asia and China[144]. In sub-Saharan Africa, an estimated 40 million women of reproductive age and 7 million pregnant women are infected with hookworm[145]. High rates of hookworm infection have also been documented in Asia[20] and South America[146]. Pullan[147] provides population prevalence estimates of soil-transmitted helminths as follows:

- South and South East Asia—over 20% (over 50% in Nepal, Bangladesh, Burma, Vietnam, Malaysia, New Guinea, Philippines)
- over 20% in most countries in southern, eastern and central Africa (over 50% in Madagascar, South Africa, Congo Brazzaville, Gabon)
- over 20% in most countries in Latin America and the Caribbean (over 50% in Venezuela, Ecuador and Guatemala).

Until 1995, anti-helminthics were not recommended at all during pregnancy due to concerns about potential teratogenesis. There has been limited research since then on associations between helminth infections and maternal anemia[145, 148]. As noted elsewhere in this paper, maternal anemia in turn is associated with low birthweight and elevated risk of newborn death.

#### Evidence

Antihelminthic trials have generally given iron-folic acid to both intervention and control groups, which may attenuate the expected effects on maternal hemoglobin or birthweight in the absence of supplementation. There are differences in absorption and effectiveness between anthelminthics; a systematic review[145] found that albendazole was more effective than mebendazole in tempering the decline of Hgb levels during pregnancy but was more poorly absorbed. Most trials to date have been conducted in areas of high hookworm prevalence but low total worm burden (different results could obtain in areas with other characteristics)[149]. Variability across settings (varying intensities of infection, different etiologies of anemia, etc.) could have affected some results within individual studies[145], though there have been studies that have found no interaction by intensity of infection[146].

In Haider’s Cochrane review[150], overall no effect was seen on anemia rates, low birthweight, perinatal mortality or preterm birth. The review included 3 RCTs of single-dose antenatal deworming:

- Elliot [149]: Uganda, N = 103, baseline hookworm prevalence 38%, single-dose albendazole;
- Torlesse [151]: Sierra Leone, N = 125, baseline hookworm prevalence 66%, single dose albendazole, 2nd trimester;
- Laroque [146]: Peru, N = 1042, baseline hookworm prevalence 47%, single dose mebendazole. Incidence of very low birthweight was reduced in the intervention arm (0 vs. 7 in the control arm).

An updated Cochrane review[152] identified one additional trial meeting its inclusion criteria [153] but this study did not address outcomes of interest for the current review. In a subsequently published RCT with a much larger sample size[154], 2507 pregnant women were recruited to participate in a 2x2 factorial trial of albendazole &/or praziquantel vs. placebo. Baseline prevalence of hookworm was 45% (15% of whom had moderate or heavy infection) and of schistosomiasis was 18%; the mean baseline hemoglobin was 11.5 g/dL. The intervention was single-dose albendazole at first ANC contact (either 2nd or 3rd trimester). Hookworm prevalence at term was much lower in the albendazole-treated group, however anemia prevalence at term was no different between treatment groups. Similarly, there was no effect on birthweight. However women with moderate to heavy intensity hookworm infection who received albendazole had a lower risk of anemia (RR = 0.45, 95% CI: 0.21–0.98). Perinatal mortality did not differ by treatment group.

In an earlier *observational study*, De Silva[148] found a significant lower rate of very low birthweight infants among those reporting having taken mebendazole (1.1%) versus those reporting no use (2.3%) (OR 0.47, 95% CI: 0.32–0.71). There was no effort made in this study to measure and control for potential confounding. In another observational study[20] the intervention was 2 doses of albendazole, one in the second trimester and another in the third. This was intended to be given to all participants in an associated multi-micronutrient RCT. Sample size was 3,327 for those for whom a birthweight measure was available; 851 for Hgb. This study compared outcomes of interest between women receiving the intended 2 doses with those receiving one (15% of the total sample) or none (10% of the sample), controlling for measured potential confounders. Baseline hookworm rate was 74%, 38% of which had moderate or heavy infection. Albendazole receipt was associated with significantly lower probability of severe anemia. Birthweight was significantly higher among those receiving 2 doses, and mortality by 6 months of age lower (RR = 0.59, 95% CI: 0.43–0.82), adjusting for nutrient supplementation group, maternal parity, tobacco smoking, early pregnancy weight, height, ethnic group, literacy, gestational duration of pregnancy, and social status). Although there was measurement of potentially important confounders and there was appropriate adjustment made in the analysis, having used an observational design the observed effect may be accounted for at least in part by residual confounding. That said, this is the one study in which antihelminthics were given in both the second and third trimester. The dose-response effect (for 2 vs. 1 vs. 0 doses) seen in this study adds weight to the findings.

There have been 2 reviews published since Haider’s Cochrane review, by Elliot[149] and Imhoff- Kunsch[144]. Based on its findings, Elliott et al suggest moving away from universal dosing due to overall little improvement in birth outcomes and potential side effects and recommended more targeted use. However, Imhoff- Kunsch[144] concluded that continued use is warranted in hookworm-endemic settings where anemia is prevalent, and they support concomitant provision of iron supplements where there are high rates of anemia because, in their words:

- there is insufficient evidence from RCTs to conclude that there is no benefit of deworming (very few RCTs in only select regions, most in populations with low total worm burden),
- observational studies have shown a potential health benefit of antihelminthic treatment in pregnancy,
- albendazole or mebendazole given after the first trimester of pregnancy is not associated with risk of congenital abnormalities,
- studies have reported an inverse relationship between maternal anemia and helminth infection intensity,
- studies in schoolchildren have reported improvements in health, growth and developmental outcomes with deworming, and
- studies in non-pregnant populations report a benefit of deworming on anemia.

**Conclusion:** There is no clear benefit with regard to our primary outcomes of interest, even in settings with high helminth prevalence. But the evidence is insufficient to recommend discontinuing use in settings where anti- helminthics are routinely given in pregnancy.

#### Malaria Prevention

Malaria remains a major public health problem through much of sub-Saharan Africa and Southeast Asia [155]. Pregnant women and their fetuses and newborns are particularly vulnerable—with malaria in pregnancy responsible for increased risk of stillbirth, intrauterine growth restriction (IUGR), neonatal and maternal death, and later infant and child deaths. Steketee, in a review of 20 studies of malaria in pregnancy from across Sub-Saharan Africa[156], found a mean prevalence of peripheral or placental parasitemia of 28%. Placental malaria doubles the risk of low birthweight[157]. Eisele[158] has calculated that malaria accounts for up to 14% of all low birthweight infants worldwide and 11% of low birthweight-attributable infant mortality in Africa. Based on their review, Murphy and Breman[159] estimate that “between 167,000 and 967,000 cases of malaria-associated LBWt occur yearly [and that] malaria-induced LBWt kills 62,000–363,000 newborns each year.”

Walker[160] estimates that approximately 900,000 LBWt deliveries/ year in Africa can be attributed to malaria (i.e. about 7/100 live births). Brabin[161] estimates that in high transmission areas the contribution of malaria to overall maternal mortality ranges from 0.5 to 23.0%. In a hospital-based study in Lusaka, Zambia, Ahmed[162] found that 17% of maternal deaths were attributable to malaria. Similarly, Menendez in a study in Mozambique[163] found an attributable proportion of 10%.

For two interventions, there is now comparatively robust evidence of effectiveness during pregnancy: the use of insecticide-treated bednets (ITNs) and intermittent presumptive treatment in pregnancy (IPTp) using sulfodoxine-pyrimethamine (SP). Trials have been conducted testing each separately, and with the two combined. Some of the trials have included only laboratory endpoints, notably peripheral or placental parasitemia or hemoglobin levels.

The principal interest of this review is *clinical* endpoints—notably death (including stillbirth), but also intrauterine growth restriction and prematurity. In the case of malaria-in-pregnancy interventions, such clinically meaningful endpoints have been assessed in a number of trials, so for these interventions we will not be focusing on studies restricted to intermediate, laboratory-based endpoints. We retained studies (both for IPTp and for ITNs) if they used one of the following outcomes: intrauterine growth restriction, prematurity, low birth weight, perinatal mortality, or neonatal mortality. Trials comparing SP to other antimalarials were excluded, as were studies comparing 2-dose regimens with 3-dose or monthly regimens. Finally, because there is adequate evidence available from RCTs, observational studies were not retained in our review with the exception of one very large-scale study[158] addressing our issues of interest, a pooled analysis of datasets from population-based household surveys conducted in 25 African countries between 2000 and 2010. Data in this study are as reported by women survey respondents who had given birth over the 2 years preceding the survey. The total sample was 140,000. This study used low birthweight and neonatal mortality as endpoints; and it considers IPTp (2 or more doses) and household net ownership of ITNs (for at least 6 months preceding the birth) separately and in combination.

**Insecticide-Treated Nets:** In the Eisele study[158], the investigators did not have access to data on ITN *use during pregnancy*, but instead on *household net ownership at the time of the survey*. There was no reduction in low birthweight associated with household net ownership and no mortality effect. But given evidence from other studies for effectiveness of ITN use by pregnant women in reducing low birthweight (see below), the authors speculate that their “null finding was at least partly caused by measurement error as the result of the inability to directly measure ITN use by pregnant women,” given the nature of the data they were using.

A Cochrane review published in 2006[164], reported on the results of 4 African RCTs testing ITNs vs. no nets and found 23% lower risk of LBW (RR = 0.77, 95% CI: 0.61–0.98), and 33% lower risk of miscarriages and stillbirths (RR = 0.66, 95% CI: 0.47–0.97) among women with parity 1–4, but no effect among those of higher parity. The studies reviewed did not include neonatal or infant mortality as endpoints.

In a review developed to generate effect sizes for the LiST tool for stillbirth, Ishaque[140] retained 6 trials of IPTp and/or ITNs, and did pooled analysis finding a non-significant reduction (RR = 0.78, 95% CI: 0.59–1.03). Restricting the analysis to studies assessing ITNs, they found reduced fetal loss (RR = 0.67, 95% CI: 0.47–0.97).

Eisele’s review[165] was the basis for LBWt effect sizes adopted for the LiST tool. Note that this review did not include any additional studies beyond those considered in the Cochrane review. In their pooled analysis for the protective effect of ITNs and/or IPTp in first or second pregnancies, they found a protective efficacy of 35% (95% CI: 23%–45%).

Since publication of Gamble’s Cochrane review, Kabanywanyi[166] and Oduro[167] have published results from ITN trials showing benefit with regard to low birthweight, in line with the earlier studies. Ndyomugyenyi[168] published a trial from a setting with low, unstable transmission which did not show such an effect.

While most trials conducted to date have shown reduced low birthweight—averaging about 25%—and the Cochrane review by Gamble showed a pooled effect size of 33% for miscarriages and stillbirths—these trials of ITN use in pregnancy have not provided evidence on neonatal mortality risk. That said, the few trials to date that have looked at neonatal or perinatal mortality, with the exception of the very large survey-based meta-analysis by Eisele[158] and the comparatively large trial by ter Kuile in a low transmission setting[169], have been beset by small sample sizes and methodologic problems. So, a neonatal mortality effect cannot be ruled out.

**Intermittent Presumptive Treatment in Pregnancy:** For Intermittent Presumptive Treatment in Pregnancy (IPTp), we have the result of Eisele’s survey-based meta-analysis, described above[165]. Among those receiving IPTp alone, risk of LBWt was 25% lower than among controls (RR = 0.75, 95% CI: 0.71–0.80), with no difference observed by parity. Results were similar for neonatal mortality (RR = 0.80, 95% CI: 0.70–0.90), again with no difference by parity.

Garner and Gulmezoglu’s Cochrane review[170] showed reduced risk of low birthweight (RR = 0.57, 95% CI: 0.46–0.72)–all parities. Sample sizes of the retained trials were small, leaving the meta-analysis underpowered for looking at stillbirths and neonatal mortality. However, there was a borderline significant effect for perinatal mortality in the first 2 pregnancies (RR = 0.73, 95% CI: 0.53–0.99).

Several more trials have been conducted since the Cochrane review comparing IPTp with placebo. Like the trials included in the Cochrane review, Gies[171] and Gutman[172] showed reduced risk of low birthweight. Ndoyomugyeni[168], in an area of low malaria transmission, did not show an effect on incidence of low birthweight (similar to their result for ITNs, which were tested in the same trial). Menendez[173], reporting on a trial in Mozambique testing IPTp+ITNs vs. ITNs alone, documented a reduced risk of newborn death (RR = 0.39%, 95% CI: 0.16–0.93). This result was anomalous, in that most trials testing the combination of IPTp+ITNs with either ITNs alone or IPTp alone have not shown significant differences in outcome.

Radeva-Petrova et al’s updated Cochrane review on malaria chemoprophylaxis in pregnancy[174] came to similar conclusions to the earlier review by Garner and Gulmezoglu. This updated review retained 17 trials, including 6 not included in the earlier Cochrane review, with diverse drug regimens and recruitment criteria. Malaria chemoprevention reduced the risk of moderate to severe anemia, among women in the first or second pregnancy, by 40% (RR = 0.60, 95% CI: 0.47–0.75, 3 trials). Likewise, in such pregnancies, LBWt was reduced by 27% (RR = 0.73, 95% CI: 0.61–0.87, 8 trials). The authors report that for miscarriage and stillbirth, and for perinatal (RR = 0.73, 95% CI: 0.54–1.00, 2 trials) and neonatal mortality (RR = 0.62, 95% CI: 0.37–1.05, 2 trials), the analyses were underpowered to detect an effect. Benefits were not seen in higher parity.

Restricting to trials specifically testing the efficacy of sulfadoxine-pyrimethamine (SP) for presumptive treatment, the pooled effect size for moderate to severe maternal anemia was the same as for malaria chemoprevention (RR = 0.60). For SP, parity 0 or 1, risk of miscarriage was reduced (RR = 0.61, 95% CI: 0.8–0.99, 3 trials) as was risk of low birthweight (RR = 0.81, 95% CI: 0.67–0.99). A statistically significant effect was not found for neonatal mortality (RR = 0.62, 95% CI: 0.37–1.05) or preterm birth (RR = 0.85, 95% CI: 0.66–1.10).

**Conclusion:** We have good evidence that both IPTp and ITN use during pregnancy reduce risk of low birthweight, with a reduction of risk that could be 40% or more. We have some suggestion for an effect for IPTp on newborn mortality (from Eisele[165]), with a calculated effect size of 20%. While it is credible that ITNs may have such an effect, strong evidence is lacking.

Direct evidence for effectiveness of IPTp or ITNs for reduction of risk of stillbirths is sparse. Bhutta[14], based on the documented prevalence of placental malaria in malarious areas and the increased risk of stillbirths associated with placental malaria, estimated a potential reduction in stillbirth risk in these populations of approximately 20% with use of either IPTp or ITNs (RR = 0.78, 95% CI: 0.59–1.03).

As already noted, malaria is estimated to account for approximately 10% of maternal deaths in malarious areas[161] however direct evidence is not available for the effectiveness of IPTp or ITNs in reducing this risk. It is entirely plausible that some significant proportion of this 10% is preventable through use of these interventions. In Pollard[175], using a Delphi process with a group of content experts, it was estimated that one could expect reduction in malaria-specific maternal mortality of approximately 3/4s with use of IPTp and/or ITNs.

Similarly, low birthweight associated with intrauterine growth restriction is a known risk factor for stunting [19] and for mortality at ages 1–59 months[18]. There is no reason to believe that growth restriction resulting from malaria in pregnancy—preventable through IPTp or ITN use—does not have these longer term consequences, and therefore it is credible that the benefits of IPTp and ITN use would include growth and mortality effects later in infancy and childhood.

In all settings with endemic *Plasmodium falciparum*, improved maternal and newborn outcomes can be expected with these 2 interventions. Proportionate contribution to mortality reduction will depend on population malaria burden.

As van Eijk reports[176], among 47 malaria-endemic countries surveyed, 45 reported having policies of ITN distribution to pregnant women, however mean coverage for ITN use in pregnancy in these countries was only 17%. In general, coverage was lowest in countries with the *highest* transmission (with a few positive exceptions, e.g. Zambia). Among 22 malaria-endemic countries with recent DHS data on intervention coverage during pregnancies over the preceding 2 years, on average only 20% of women reported receiving at least 2 doses of SP[5]. Two recent papers address strategies for increasing coverage of these essential interventions[177, 178].

#### Tetanus Toxoid

In 2010, among vaccine-preventable diseases, tetanus was second only to measles as a cause of mortality among children[179]. However, significant progress has been made in the past 30 years as global efforts have resulted in a reduction of 93%, from an estimated 787,000 neonatal deaths due to neonatal tetanus in 1988[180] to an estimated 58,000 deaths in 2010[181]. High-income countries have very low rates of tetanus-related mortality and some low- and middle-income countries (such as Nepal, Bangladesh and Turkey) have made significant progress towards the same[179]. Certain populous countries, such as India and Nigeria, have made progress but still have comparatively high rates of tetanus-attributable deaths in newborns. Fourteen percent of neonatal deaths in a hospital study in Nigeria (n = 3,051) were attributable to tetanus[182] while modeling based on district-level household surveys (N = ~60,000) estimated 6% of neonatal deaths in India as potentially attributable to a lack of tetanus vaccination during pregnancy [183].

Neonatal tetanus, most often contracted via the cut umbilical cord, presents during the first 3–14 days of life, causing the loss of the ability to suck, muscle rigidity and spasms [184]. Case fatality rates without medical care can approach 100%; among the studies captured for this paper, case fatality rates for newborns treated in a medical facility ranged from 51% to 76%[182, 184–186]. Prevention includes clean cord care and other “cleans” as well as administration of tetanus toxoid vaccine prenatally[184, 187]. The WHO recommends 2 doses during pregnancy, at least one month apart and the first dose as soon as possible during the pregnancy, with an additional booster dose during subsequent pregnancies (up to 5 doses total)[188].

Note that other methods of preventing tetanus-attributable neonatal mortality, such as the use of chlorhexidine and various clean cord practices are addressed elsewhere in this paper.

**Evidence:** The majority of studies captured and analyzed were observational, with one RCT included comparing cholera toxoid to tetanus diphtheria toxoid. Almost all studies compared mortality rates amongst neonates whose mothers received 1–2 doses of tetanus toxoid during pregnancy to neonates whose mothers received no tetanus toxoid vaccination during pregnancy and did not have existing immunity during pregnancy from earlier vaccination. Many studies compared 2 doses of tetanus toxoid to none, while several included 1 dose of TT as well[189–192]. Several studies captured were broader[183, 193, 194], looking at the effect of various antenatal care interventions with the inclusion of tetanus toxoid.

A study conducted in northern India[187] followed 1,688 newborns of mothers with varying tetanus toxoid immunization status prospectively, finding an approximate 88% reduction in risk of neonatal mortality attributable to tetanus (95% CI: 59%–98%) amongst newborns of mothers with complete antenatal immunization (2 doses at least 1 month apart).

A Cochrane review on the prevention of neonatal tetanus[195] retained 2 older randomized and quasi- randomized trials[196, 197] with a combined sample size of 10,560, using an influenza vaccine as a comparator in one study and cholera toxoid in another, in comparison to tetanus diphtheria toxoid, with neonatal mortality as outcome, and showwed results suggestive of high efficacy. Two other reviews were captured by our search[179, 184]; the former looking specifically at the impact of tetanus toxoid (2 doses) and finding a pooled RR of 0.062 (95% CI: 0.02–0.20) across the 2 trials retained.

**Conclusion:** Almost all reviewed studies showed a very substantial reduction in the risk of neonatal mortality caused by tetanus. When the mother is fully immunized for tetanus toxoid, protection to the newborn against tetanus approaches 100%. Since case-fatality is very high even with good medical care and tetanus spores are ubiquitous, it is important where TT coverage is high that it be maintained. In settings (like northern Nigeria) where coverage is relatively poor and tetanus still accounts for a significant proportion of newborn deaths, considerable population-level impact is achievable if high levels of TT coverage can be reached.

#### Chlorhexidine Advance Distribution

Sepsis, pneumonia and meningitis together account for approximately a third of newborn deaths. Early exposure to potential pathogens, particularly through the umbilical cord stump, could represent an important remediable contributor to bacterial infection deaths. Antiseptics have long been used for umbilical cord care, although WHO recommendations from 1999 suggest that unless there is a particularly high risk of unhygienic exposures, use of antimicrobial products is unnecessary. However, since that time a number of studies have been published suggesting mortality reduction benefit of chlorhexidine. WHO now recommends its use in countries with high newborn mortality[198].

For the purposes of this review, we focused on interventions delivered during pregnancy. In settings where a significant proportion of births take place at home without the assistance of a health professional there is potential to make certain interventions available or to influence practices around birth and the first hours and days of life through contact during pregnancy, either during antenatal care or contact with community health workers. Advance provision of chlorhexidine one such intervention.

Only chlorhexidine interventions that could lend themselves to advance distribution for use by family members were considered. Therefore cord application and whole-body wipes were retained but vaginal cleansing was not. Only studies from low and middle income countries were included in this review.

**Evidence:** There have been 3 cluster randomized controlled trials published relevant to the mortality risk reduction effectiveness of chlorhexidine application to the *umbilical cord stump*—by Mullany[199], Arifeen[200], and Soofi[201]. A pooled analysis of the results of these trials was done by Imdad[202]. All 3 trials used a 7.1% w/v aqueous solution of chlorhexidine. All were conducted in south Asia, with delivery of the intervention at household level. The Soofi study, conducted in Pakistan, included only home births attended by traditional birth attendants (TBA). The other 2 studies (in Nepal and Bangladesh) were directed primarily to newborns giving birth at home, but also included a smaller proportion born in health facilities.

There were differences in the application protocols:

- In the Pakistan study, day of birth application was done by the TBA, with subsequent daily applications continued for 2 weeks by family members.
- In the Nepal study, application was by study field staff. Per protocol, it was to be initiated on the day of birth. However for 27% of those randomized to chlorhexidine, first application was more than 24 hours after birth. In the Nepal study, there were to be 7 applications over the first 10 days of life.
- In the Bangladesh study, there were 2 intervention arms: single application on the day of birth, and daily application for the first 7 days of life, with application done by study staff.

There was diversity across the 3 study populations in cord-care practices, with application of other materials by most mothers in the Pakistan study[201], by very few in the Bangladesh study[200] and by an intermediate proportion in Nepal[199]. All 3 studies showed reduced mortality risk. By per protocol analysis, the Nepal study showed a mortality effect of RR = 0.76 (95% CI: 0.55–1.04). Restricting the analysis to those reached on the first day of life, the mortality effect was RR = 0.66 (95% CI: 0.46–0.95). Among those randomized to the treatment arm who were first reached *beyond* the day of birth, mortality risk was no different than in the control group, RR = 1.02 (95% CI: 0.54–1.92). In the Bangladesh study, the multi-day arm showed a RR of 0.94, which was not statistically significant, while the single-application arm had a RR of 0.80 (95% CI: 0.65–0.98). The p-value on the difference between the 2 intervention arms was p = 0.14, and the investigators concluded that the apparently poorer performance in the multi-day arm was a result of chance. In the Pakistan study, the mortality reduction effect size was RR = 0.62 (95% CI: 0.45–0.85).

A meta-analysis, including the 3 studies[202], found a pooled effect size of 0.77 (95% CI: 0.63–0.94). Restricting the analysis to births taking place in a health facility (from the Nepal and Bangladesh studies), the effect size was RR = 0.50 (95% CI: 0.27–0.92). Note that the effect sizes given for the meta-analysis and for the 3 studies above do not refer to impact on *overall* neonatal mortality, since newborns dying very early (for example, due to asphyxia) would not have had an opportunity to be enrolled in these trials.

Two further trials[203, 204] have been conducted with study populations with considerably lower background newborn mortality, results of which have not yet been published.

*Whole-body wipes* or sponge-baths using chlorhexidine were first assessed in before-and-after studies conducted in Malawi[205] and Egypt[206], both using mortality as an endpoint. In these 2 studies, the cleansing interventions tested included vaginal cleansing and swabbing the newborn using chlorhexidine. The Malawi before-after study showed a trend towards lower mortality during the intervention period (28.6 vs. 36.9/1000 at baseline, p = 0.06), and lower infection-attributable mortality (2.4 vs. 7.3/1000, p<0.005). Likewise, the Egyptian study[206] showed reduced mortality (28 vs. 42/1000, p = 0.01). Subsequently, there have been 3 large randomized controlled trials of whole-body wipes, also with mortality as an endpoint—one hospital- based (Cutland[207], in South Africa) and 2 community-based (Tielsch[208], in Nepal, and Saleem[209] in Pakistan). These trials did not include vaginal cleansing. The South African hospital-based RCT[207] demonstrated lower mortality among newborns randomized to chlorhexidine (8.3 vs. 12.8/1000, p = 0.049). However, most of the excess deaths in the control group occurred within 9 hours of delivery. Therefore, the authors do not attribute the observed mortality difference to the intervention. These before-and-after and RCT studies were retained in a meta-analysis[210] along with several smaller studies inadequately powered to investigate a mortality effect. This analysis did not show a reduction in mortality risk.

**Conclusion:** Application of chlorhexidine to the umbilical cord-stump can very substantially reduce deaths that otherwise would have occurred due to sepsis, at least in settings such as those where the 3 published cord-care trials were conducted, with reductions in overall newborn mortality up to 15–20%. Of course in settings where sepsis arising from such exposure is much less common, this intervention can be expected to have much less impact.

As an antenatal intervention, a suitable chlorhexidine product would need to be made available late in pregnancy to women at risk of delivery at home. This strategy is currently implemented at scale in Nepal and is being introduced elsewhere.

#### Syphilis Screening and Treatment

It is estimated that globally, between 1.2 and 1.6 million pregnant women per year have probable active syphilis[211], resulting in:

- between 175,000 and 250,000 stillbirths (>28 weeks gestation) or early fetal deaths (22–28 weeks),
- 75–110,000 neonatal deaths,
- 60–70,000 preterm or low birthweight infants, and
- 120–190,000 infected newborns.

With current levels of antenatal screening and treatment, only about a quarter of expected adverse events are being averted. Africa is the most affected region, with a little over 2% of ANC attendees seropositive for syphilis. In several countries in the region, however, rates are over 5%, notably in south-east Africa (Madagascar, Mozambique, Zambia); also Chad, Central African Republic, and Ghana. In areas of high syphilis prevalence, up to half of *all* stillbirths can be attributed to syphilis[212].

The quality of evidence available is limited by a number of factors. Even with a standardized screening and treatment protocol, in a given study setting there will be considerable diversity in what point along the disease evolution a woman is at the time of diagnosis and treatment. Given that penicillin is known to be efficacious, it would be ethically unacceptable to conduct trials that entail withholding treatment. With observational research methodologies it is generally not possible to adequately control for all possible sources of confounding. Furthermore, the intervention considered has several elements, including—first—diagnosis, which may include a single step or multiple steps, and—second—treatment.

Programs that have sought to make antenatal syphilis screening and treatment services more readily available have depended on one-step, point-of-service testing. Non-treponemal tests (notably RPR, with titres >1:4) have had good specificity for active infection. Treponemal tests, like the ICS rapid tests now being used do not differentiate active, untreated infection from treated infection. Penicillin is a well-established treatment, with the standard now being use of benzanthine penicillin at a dose of 2.4 million units. A single dose is normally sufficient to eradicate syphilis in the fetus, but the mother is not considered adequately treated without an additional 2 doses[213].

**Evidence:** In a meta-analysis of 8 observational studies with stillbirth as an outcome, comparing those receiving treatment with syphilis-infected cases that did not receive treatment[213], (risk was very substantially reduced (RR = 0.18, 95% CI: 0.10–0.33). However, in these studies there was no attempt to control for confounding. Blencowe also reports that studies comparing stillbirth rates among treated syphilis-infected cases to non-infected cases, rates were generally similar, further confirming that screening and treatment prevents most stillbirths that would otherwise have resulted from syphilis infection. Blencowe reports on 4 other studies conducted in southern Africa that used perinatal mortality as an endpoint, not disaggregating stillbirth from newborn deaths, in 2 of which there were efforts to control for confounding—with results broadly similar across the 4 studies (RR in the range of 0.34 to 0.65). Five studies used all-cause newborn mortality as an outcome, comparing treated vs. infected women who did not receive treatment. They showed a pooled effect size similar to that observed for stillbirth (RR = 0.20, 95% CI: 0.13–0.32). Two of these studies were the US, one in South Africa, another in the Russian Federation and the last in Zimbabwe.

Blencowe also presents evidence for significant reduction in preterm birth, from 7 observational studies which did not control for confounding and 2 using paired cohorts. Overall, this meta-analysis yielded an effect size of RR = 0.65 (95% CI: 0.26–0.47).

Programmatic strategies have been tested to increase coverage of timely and appropriate screening and treatment[214], most of which entail single-visit, point-of-service antenatal testing and administration of the first treatment dose. There was considerable diversity with regard to context and approach but a clear pattern across studies of improved outcomes, with marked reduction in risk of perinatal mortality and of stillbirth.

**Conclusion:** Timely screening and adequate treatment of active syphilis in pregnancy can prevent most instances of serious adverse outcomes: 97% of congenital syphilis, 82% of syphilis-attributable stillbirths, and 80% of syphilis-attributable newborn deaths[213]. In settings with high syphilis prevalence, achieving high coverage for timely antenatal screening and appropriate treatment of syphilis in pregnant women has the potential to prevent many stillbirths, in some settings close to half.

Based on the burden estimates presented above, in a fairly typical African context with NMR 30/1000 and a rate of active untreated syphilis of 2% of pregnancies, timely and appropriate treatment can be expected to reduce all-cause neonatal mortality by approximately 5% and stillbirths by 12%. In settings with similar NMR but active syphilis rates of 5%, successfully identifying and treating all cases on a timely basis would be expected to reduce all-cause NMR by about 12% and stillbirths by 30%. In such settings one might even consider universal presumptive treatment as is done with malaria IPTp.

#### HIV/PMTCT

In the 31 countries in Sub-Saharan Africa for which there are DHS data on HIV testing in ANC (as of 1 May 2015 –Stat-Compiler), the median proportion of women pregnant over the previous 3 years reporting at least one ANC visit was 93%. However, the proportion reporting having had an HIV test and received the result was only 41%. Among the 10 countries on this list with adult HIV prevalence of 5% or higher, the median value for any ANC visits was 96% but, for HIV testing, only 70%. In Lesotho and Zambia, where coverage was the lowest of these countries, the proportions tested were only 43% and 40%, respectively. Perhaps not surprisingly, there are still approximately 240,000 new pediatric HIV infections per year[215], most acquired perinatally. Such infections are largely preventable but that requires, first, that HIV+ pregnant women be identified. Furthermore, in settings where most of those infected with HIV have not been diagnosed, ANC offers an opportunity for case-detection and initiation of ongoing treatment.

Program standards in this area have been evolving relatively quickly: from opt-in to opt-out provisions for testing; and from simple, short-course, one- or 2-drug, preventive regimens to combined antiretroviral therapy (ART), including provisions for life-long treatment of the mother. The WHO 2013 guidelines[216] recommend providing either:

- lifelong ART to all pregnant and breastfeeding women living with HIV regardless of CD4 count or clinical stage, or
- ART (ARV drugs) for pregnant and breastfeeding women with HIV during the mother-to-child transmission risk period and then continuing lifelong ART for those women eligible for treatment for their own health.

Papers of interest for this review address effectiveness of strategies for detecting HIV infection in pregnant women and initiating appropriate ARV use (either for PMTCT or treatment), particularly at the most peripheral level antenatal services, where laboratory capacity (e.g. for determining CD4 count) may not be available and services may be provided by health auxiliary level workers.

**Evidence:** Without treatment, risk of transmission in non-breastfeeding populations in resource-poor populations is in the range of 15–30% (although transmission rates up to 48% have been documented in untreated non-breast- feeding infants in some settings), with 2/3s to 3/4s occurring intrapartum[217]. Breastfeeding results in an additional 5–20% risk.

In a trial of newborn *post-exposure prophylaxis* comparing single-dose nevirapine (NVP) vs. NVP plus twice-daily zidovudine (ZDV) for 1 week[218], among those HIV negative at birth, by 6–8 weeks of age 12.1% of those receiving *only* NVP were infected vs. 7.7% of those receiving the 2 drugs. Overall infection rates by 6–8 weeks of age were 20.9% for the single-dose regimen and 15.3% for the 2-dose regimen. There was no placebo comparator, so the study provides no evidence on what the transmission rate would have been in the absence of ARVs, although the data reported by de Cock[217], above, give some notion of the order of magnitude.

For breastfeeding populations, ZDV plus 3TC given to mothers during labor only (PETRA ‘regimen C’) did not reduce transmission compared to placebo [219]. Beginning earlier makes a difference: as Sigfried concludes in a recent Cochrane review[220] reviewing published and unpublished data from the Kesho Bora study[221], ZDV given to mothers during the antenatal period, followed by ZDV+3TC intrapartum and postpartum for one week, and single-dose NVP given to infants within 72 hours of delivery and ZDV for one week, can be effective. Compared to triple therapy beginning before 34 weeks gestation (and continuing to 6 months of age), there was no statistically significant difference between groups in HIV infection rates at birth or by 6 months of age. Among exposed newborns in the short-course arm, HIV transmission at birth was 5.0%. Among those breastfeeding, HIV transmission status by 12 months was superior in the triple ARV arm, with 5.6% infected vs. 10.7% in the short-course arm (p = 0.02). But in comparison with expected infection rates *without* treatment, as determined by de Cock[217], either of these treatments give markedly better outcomes.

In a cohort study conducted in Zambia assessing risk of perinatal transmission by timing of initiation[222], those on antenatal HAART for 4 weeks or less had 5.2 fold higher odds of transmission than those initiating at least 13 weeks before birth. So starting earlier makes a difference.

As Sigfried[220] notes, ZDV monotherapy is also useful, especially if it includes a long antenatal treatment component (although this is no longer a recommended treatment). Connor [223], tested ZDV monotherapy given during pregnancy beginning at 14–34 weeks gestation, given intrapartum and then to the newborn for 6 weeks; at 18 months, they found 8.3% of those in the treatment arm HIV+ vs. 25.5% in the placebo arm. However, to achieve these benefits, women who are HIV+ need to be identified. If identified before or during pregnancy, they can be offered effective short or longer-course ARV treatments (including initiating life-long treatment for those eligible).

Typically, there is significant loss along the “cascade” from screening through completion of effective treatment. In a systematic review of 41 descriptive studies in low and middle-income countries, Tudor Car[224] found that although the proportion of those attending ANC who were tested was high, the majority of those who were HIV+ did *not* receive ARV prophylaxis in antenatal care. Wettstein[225] did a review which included 44 studies from 15 countries across sub-Saharan Africa, assessing performance along the cascade from testing through treatment. They concluded that treatment coverage is improved by providing ARV treatment beginning in the ANC clinic to those women testing positive, rather than requiring referral. Similarly, in a subsequent large quasi-experimental study in Cape Town, South Africa, Stinson[226] found higher antenatal initiation of ARVs using a model in which this service was offered at the same point of service as routine ANC.

In a recent study in Zambia, South Africa, Cote d’Ivoire, and Cameroon, based on a household survey of women having given birth over the previous 2 years[227], among those documented to be HIV+ at the time of the survey, 98% reported they had received at least some antenatal care and 87% received HIV testing during pregnancy but fewer than half reported receiving their test result and, overall, only 36% received both antenatal and infant ARV prophylaxis. The biggest decrement in the cascade was between having accepting HIV testing and receiving a positive test result, even though rapid HIV testing algorithms were in use in the study settings. Although it is not possible to disentangle from the study results, it appears that the apparent performance decrement may be a function of both service provider and beneficiary-related factors.

With service delivery complications—including equipping health workers to do counseling, testing, and treatment initiation at peripheral level on an integrated basis with other aspects of ANC—and beneficiary- side factors—including stigma and fear of disclosure—it can be a significant programmatic challenge to achieve high population coverage. However, a robust ANC platform that is functional at the most peripheral level and able to provide both HIV testing and initiation of antenatal ARVs on an integrated basis appears to offer the greatest promise for effectively reaching a high proportion of those requiring this service.

Clearly, too, robust services are required across the service delivery continuum, through labor and delivery care and the postnatal period, to ensure adherence to appropriate ARV regimens and optimal feeding practices.

**Conclusion:** Most cases of perinatal transmission can be prevented. Combined ART regimens are most effective but even ZDV monotherapy initiated early (<34 weeks gestation) can prevent most cases. Population-level effectiveness requires strategies that reach a high proportion of women through ANC and that minimize loss across the cascade.

#### Infection: Summary

As outlined in Table 3 (below), in affected populations there are a number of infection-related interventions that have significant potential impact on the outcomes considered in this review. The battle against neonatal tetanus can be considered a global health success. However, there remain subpopulations with low antenatal tetanus toxoid coverage and significant neonatal tetanus disease burden, notably in certain parts of south Asia and northern Nigeria. In these settings, special efforts are needed. Elsewhere, the high levels of coverage that have been achieved need to be sustained.

**Table 3:** Summary of Effects of Infection-Related Interventions on Outcomes of Interest.

Intervention	Effects on:						Comments
	Maternal mortality	Newborn mortality	Stillbirth/ miscarriage	Preterm	IUGR/ LBWt	Other outcomes	
**Deworming**		/		/	/		
**IPTp/ ITNs**	Up to ~8–10%↓ in highly malarious areas?; studies not powered for a definitive estimation [161, 175]	About 20%↓ for IPTp in malarious areas; effect for ITNs may be similar but evidence lacking [165]	~25–30%↓ both for IPTp & ITNs [14, 164]		~25%↓ both for IPTp & ITNs [165]	Expected knock-on effects on stunting	Effectiveness estimate assumes 100% coverage.
**Tetanus toxoid**		Close to 100%↓ of tetanus-specific mortality; in certain settings w/ low TT coverage, could ↓ all- cause NMR by up to 10% [182]					In most settings, because coverage is already high there is little scope for ↑d impact, but this high coverage needs to be sustained.
**Chlorhexidine**		15–20%↓ of all-cause NMR in populations with high NMR					Effectiveness estimate assumes 100% coverage.
**Syphilis screening & treatment**		~5%↓ in all-cause NMR in many African populations; up to 10%↓ or more in some populations	~12%↓ in many African populations; up to 30%↓ or more in some [213]	35%↓ for cases with syphilis [213]			Effectiveness estimate assumes 100% coverage.
**PMTCT**						Can prevent a very high proportion of perinatal HIV transmission [220]	

Notes on table: **/** indicates that this has been assessed and there is no evidence of an effect.

In highly malarious settings, IPTp and ITNs have large magnitude effects on multiple pregnancy outcomes.

In populations with comparatively high syphilis prevalence, routine antenatal screening and treatment can make an important contribution particularly in reduction of stillbirths.

Chlorhexidine applied to the newborn’s umbilical cord stump is a new intervention that is beginning to be scaled up in several countries. Where newborn mortality is high and the contribution of sepsis is important, this intervention can significantly reduce risk of newborn death. Where many births take place at home, advance distribution during pregnancy can be a suitable delivery strategy.

### Obstetrical and Other

In addition to interventions addressing nutrition and infection, this review includes 5 interventions which can be classified as obstetrical:

- prevention of post-partum hemorrhage at home births through advance distribution of misoprostol
- timely detection and referral of pregnancy-induced hypertension/ pre-eclampsia
- clean delivery practices
- thermal care of the newborn, at the time of birth
- birth preparedness/ complication readiness

In addition, in this section we review evidence for the benefits of antenatal counseling addressing alcohol and tobacco use, and family planning.

#### Misoprostol Advance Distribution

Postpartum hemorrhage (PPH) is estimated to cause over one in 4 maternal deaths worldwide (Say 2014). Almost all of these deaths occur in LMICs. Most are due to uterine atony. The WHO recommends oxytocin as the first-line uterotonic, as it is more effective than misoprostol in reducing post-partum blood loss[228], but as an injectable drug requiring temperature-controlled storage, circumstances where it can feasibly be used are more limited than for misoprostol[229].

Misoprostol is an oral uterotonic medication given immediately after birth to reduce postpartum hemorrhage[230]. Because misoprostol is a relatively effective oral uterotonic, inexpensive, easily transportable and thermally stable, the WHO recommends its use in circumstances where use of oxytocin is not feasible[229].

It should be noted however that misoprostol degrades with exposure to humidity at the time of manufacture and, if inadequate packaging is used, during subsequent transport and storage. Furthermore, as a potent uterotonic, like oxytocin it can have unintended negative consequences with mistimed use; when taken before the woman has given birth, there have been cases of ruptured uterus. It can also be used for medically-induced abortions, making its use for other obstetrical indications sensitive in some political-cultural settings. Shivering and pyrexia are common side-effects that could compromise acceptability, although they do not represent a safety concern. Likelihood of such side-effects is dose-dependent.

**Evidence:** As is generally the case for preventive interventions for maternal outcomes, because death is a rare outcome studies tend to be underpowered for this endpoint. Not surprisingly then, the Cochrane review by Hofmeyr et al[231] specifically on misoprostol for PPH prevention (updating a review first done in 2009), considering mortality as an endpoint, found no statistically significant difference in outcome between misoprostol and placebo or, for that matter, between misoprostol and other uterotonics. As a proxy, for uterotonic medications for PPH prevention, severe blood loss (≥1000cc, or need for blood transfusion) is commonly used as an endpoint, as was the case in the Cochrane review by Tuncalp et al.[230] (updating an earlier version[232]).

The extent to which significant blood loss correlates with risk of death depends on context, e.g. where there are low rates of moderate to severe anemia, good medical care and timely access to IV fluids, blood, and procedures for controlling bleeding, this level of blood loss need not be a big concern, but in settings with much less robust care, correlation with risk of death can be much higher. Hofmeyr et al [231] found that compared to placebo, misoprostol reduced the need for blood transfusions by more than 2/3s (RR = 0.31, 95% CI: 0.10–0.94, 4 trials, 3519 women); it also reduced risk of significant blood loss (≥1000cc) although due to heterogeneity between the studies, no summary measure was calculated.

When compared against other uterotonics, risk of severe PPH with use of misoprostol was higher (RR = 1.33, 95% CI: 1.16–1.52, 17 trials), although there was a trend towards fewer blood transfusions with misoprostol (RR = 0.84, 95% CI: 0.66–1.06, 15 trials, 28,213 women). The largest of the studies comparing misoprostol with other uterotonics was Gülmezoglu[228] a multi-center WHO trial, N = 18,530, conducted in 9 countries (Argentina, China, Egypt, Ireland, Nigeria, South Africa, Switzerland, Thailand, and Vietnam).

The 3 retained trials most relevant to the current review were placebo-controlled studies conducted in low income countries at household or primary health care center level. They included the following (with relative risks given for severe PPH):

- Guinea Bissau[233], drug administered by midwives in primary care centers, N = 661, RR = 0.66 (95% CI: 0.45–0.98)
- India[234, 235], administered by auxiliary nurse midwives either at home births or in a primary care center, N = 1620, RR = 0.20 (95% CI: 0.04–0.91)
- Pakistan[236], administered by TBAs at home births, N = 1072, RR = 0.57 (95% CI: 0.27–1.22).

Other reviews relevant to misoprostol use for PPH prevention include an earlier systematic review/ meta- analysis with results similar to Tuncalp[237] and a review and meta-analysis that included disaggregating community-based trials[238]. Their meta-analysis of community-based trials includes the Guinea Bissau[233] and India[234] trials mentioned above, along with a study done in the Gambia[239] which used ergometrine as the comparator for misoprostol.

Another more recent review[240], similarly, looks at evidence from RCTs and other studies on the effectiveness and safety of community-based use of misoprostol for PPH prevention. Pooling data from 6 studies, they found a 42% reduction in risk of PPH (RR = 0.58, 95% CI: 0.38–0.87). This review included Derman but also several papers not captured in other reviews, including studies conducted in: Afganistan [241], Ethiopia [242], Indonesia[243], Bangladesh [244] and Pakistan [236].

From data presented in a modeling study[245], it was determined that for home births without access to hospital care, use of misoprostol after birth could avert up to 100 deaths per 100,000 live births. In another modeling study[246], Sutherland et al. compared use of preventive misoprostol to standard management of home births in India, using data from Derman[234]. They found a total of 108 lives saved per 100,000 women delivering at home, using misoprostol for PPH prevention, in a setting where there are an estimated 130 PPH-attributable deaths per 100,000 live births. In a more recent modeling study based on Bangladesh data[247], with current maternal mortality ratio (MMR) (approximately 200/100,000), evidence from the literature on efficacy, and cause-specific mortality in their study population, the authors determined that with “high coverage” of 80%, using misoprostol after home births approximately 13 deaths could be averted per 100,000 live births (i.e. about 6% lower than current mortality).

In an “integrative review” [248], Smith reports on documented program experience implementing community-level use of misoprostol for PPH prevention. The review addressed important implementation issues, rather than effectiveness per se. Reports found included 8 from the peer-reviewed literature and 10 from grey literature, and were drawn from programs in 15 countries across Asia and Africa. Approaches used were diverse, reflecting the diverse program settings. There were very low rates of mistimed use and no reported serious adverse effects. Higher coverage tended to be achieved with advance distribution and self- administration. Similarly, higher coverage tended to be achieved in programs relying primarily on CHW or TBA distribution, as opposed to distribution primarily through ANC contacts. Several of the programs documented were operating at relatively large scale, reaching over 10,000 pregnant women with misoprostol during the period reported; these programs or large-scale pilots were conducted in Bangladesh[249, 250], Nepal[251], and Zambia[252].

**Conclusion:** Oxytocin is the drug of choice for prevention of PPH, which remains the leading cause of maternal mortality. However, where its use is not feasible, misoprostol is an alternative which can prevent approximately 3 cases of severe PPH for every 4 cases that would otherwise have been prevented using oxytocin. Operations research studies conducted to date have demonstrated that its use at community level, with advance distribution by health workers or community health workers, can be effective and safe.

Modeling studies provide some indication of expected magnitude of benefit. Clearly, misoprostol use at home deliveries is an inadequate substitute for quality institutional births. But in settings where many births take place without ready access to such care, a significant proportion of PPH deaths that would otherwise occur can be prevented.

#### Pregnancy-Induced Hypertension/ Pre-eclampsia: Screening and Treatment

Hypertensive disorders of pregnancy are second only to hemorrhage as a direct cause of maternal mortality, and are currently estimated to cause about 14% of *maternal deaths*[253]. In sub-Saharan Africa approximately 1 in 1500 pregnancies ends in a maternal death attributable to eclampsia/pre-eclampsia; in South Asia the ratio is about 1 in 3000 (calculated from Kassebaum[254]). Eclampsia and pre-eclampsia make an even more important contribution to *perinatal mortality*. According to data from a 6-country WHO calcium supplementation trial[255], *eclampsia/ pre-eclampsia is the primary obstetrical cause for 24% of perinatal deaths*. Furthermore, as Duley reports[256], “Pre-eclampsia is an antecedent for up to 12% of infants born small for gestational age and one-fifth of those born preterm.”

How can the most peripheral level health services for pregnant women contribute to reducing this disease burden? Unlike some of the other areas addressed in this review, screening for pregnancy-induced hypertension cannot be expected *on its own* to improve outcomes but it is one element (we will argue, a key element) in a systems process to improve outcomes and is best understood within the continuum of care paradigm, with the two dimensions of time and level of care.

Conceptually, the first step is screening. This needs to reliably detect cases on a timely basis and can, in principle, be done at the most peripheral level and does not necessarily require professionally-trained health workers. With such cases detected, either by blood pressure or proteinuria, they need close case management by professionally-trained health workers, with effective links to a hospital where definitive care can be provided, which brings us to the third step—definitive care—which includes timely delivery and managing any complications arising (notably severe pre-eclampsia or eclampsia). For this to work effectively, each of the elements needs to be robust and linkages across steps and levels need to be functional.

As we will see, there is at least some evidence available on certain specific components, notably on the use of MgS0_4_ and antihypertensive drugs for managing complications. And there is evidence of a different kind for performance of the system as a whole. For the piece that is of particular interest for the current review—screening at the most peripheral level—evidence is lacking.

**Evidence:** The Cochrane review on the use of **MgSO**_**4**_ and other anticonvulsants for pre-eclampsia[257] included 6 trials that compared MgSO_4_ with either a placebo or no anticonvulsant. Use of MgSO_4_ significantly reduced the risk of developing eclampsia (RR = 0.41, 95% CI: 0.29–0.58) and placental abruption (RR = 0.64, 95% CI: 0.50–0.83). The review found a non-significant reduction in maternal death (RR = 0.54, 95% CI: 0.26–1.10), and did not find a clear difference in risk of stillbirth or newborn death. By far the largest study contributing to the Cochrane result was the Magpie trial, published in 2002[258], which was conducted in 33 high, middle and low-income countries. The trial randomized 10,141 women with eclampsia or severe pre-eclampsia to receive either MgSO_4_ or a placebo; the primary maternal outcome examined was eclampsia; when women were randomized to treatment before delivery, stillbirth and pre-discharge neonatal mortality were also considered. Those receiving MgSO_4_ had reduced risk of eclampsia (RR = 0.42, 95% CI: 0.29–0.60). Maternal mortality was lower, but the difference was not statistically significant (RR = 0.55, 95% CI: 0.26–1.14). MgSO_4_ has also been shown to be more effective than other anticonvulsants.

In a review of before-and-after, cohort, and serial cross-sectional studies in Bangladesh, India, Pakistan, UK, and Nigeria, McDonald[259] found lower rates among those receiving MgSO_4_ for: eclampsia, maternal death, recurrent seizure, neonatal mortality, and major maternal morbidity; there was no decrease in stillbirth.

Severe complications can arise from the very high blood pressure that can be associated with pre-eclampsia. There have been trials of different rapidly acting **antihypertensive drugs** used for this indication [260]. Calcium channel blockers and hydralazine have been common choices. Studies have been comparatively small and the evidence is inadequate with regard to severe endpoints, although calcium channel blockers have been shown to be superior to hydralazine in reducing persisting high blood pressure (RR = 0.37, 95% CI: 0.21–0.66). The benefits of use of antihypertensives for mild-to-moderate hypertension in pregnancy are unclear[261],

A review by Ronsmans and Campbell[99] sought to quantify the population-level effectiveness for reduction of maternal mortality of appropriate **case management** for eclampsia and severe pre-eclampsia and concludes that a reduction of 85% or more is possible where there is effective case detection and functional referral to a level at which definitive care can be provided. In an historical review of experience with hypertensive disease in pregnancy in high-income countries since the early 20th century, Goldberg et al[262] found a very marked decline in maternal mortality attributable to this condition, with most of the improvement over the period 1940 through 1970, during which incidence of eclampsia dropped by about 90% and, among women who developed eclampsia, case-fatality dropped by 90%. As the authors conclude, the “most important interventions [accounting for this decline] were widespread use of prenatal care with blood pressure and urine protein measurement, and increased access to hospital care for timely induction of labor or cesarean delivery for women with severe pre-eclampsia or seizures.”

A review by Jabeen et al.[263] specifically addressed the effects of pre-eclampsia-associated interventions on stillbirths. They reported that for calcium supplementation, there was a borderline significant reduction in stillbirths (RR = 0.81, 95% CI 0.63–1.03). For LiST, using a Delphi process, they propose an effect size for reduction in overall risk of stillbirth of 0.80 for all PE interventions combined, including screening and treatment.

Although spot microalbumin-to-creatinine ratio correlates better with 24-hour urine protein than do simple albuminuria dipsticks[264], the extent of proteinuria is not a good predictor of the severity of pre- eclampsia[265]. New, low-cost diagnostics are under development including proteinuria test strips made of filter paper and the use of inks and dyes to detect excess protein; more accurate blood pressure measurement is also becoming feasible at the community level—semi-automated measurement tools are being developed to increase the reliability of measurements taken in the field, requiring a lower level of skill than manual blood pressure cuffs[266]. However, even with existing technologies, in principle reliable, timely case- identification should be possible from the most peripheral level (Box 1).

**Box 1. Key Elements of Pre-Eclampsia Screening and Case-Management**

- **At primary care level:** Reliable early detection
- **1° level—hospital linkages:** Functional protocols, procedures, communication, coordination, logistics
- **Hospital-level management** (non-severe): Close monitoring, timely delivery
- **Additional care for severe cases:** MgSO4, control very high BP

**Conclusion:** As is evident from the experience in middle and high income countries, the overwhelming majority of maternal deaths due to pregnancy-induced hypertension can be prevented with reliable early detection, timely delivery and appropriate management of complications. Similarly, timely detection and appropriate treatment can prevent a large proportion of the 24% of all perinatal deaths resulting from eclampsia/ pre- eclampsia. While new technologies for simpler, more accurate case-detection may be helpful, existing technologies are adequate. More important is that pregnant women are adequately assessed. Justus Hofmeyr[267] has commented that: “An increased number of routine visits may detect asymptomatic conditions such as preeclampsia … allowing more timely intervention. The importance of the content and quality of routine antenatal care should not be lost to policy makers when decisions about numbers of visits with the available resources are being made.” Such case-detection can be done at the most peripheral level, even by health workers with limited training, like health auxiliaries. Having detected these cases of pre- eclampsia, to achieve improved outcomes then requires functional referral linkages to definitive care.

#### Clean Delivery Practices

An estimated 520,000 newborns die from neonatal sepsis annually (up to 15% of neonatal deaths worldwide) and 60,000 from tetanus, the majority of these in low-income countries[268, 269]. For women who end up giving birth without the assistance of a trained health worker, there is still scope to influence practices around the time of delivery by reaching her with suitable counseling or health education during pregnancy. Practices affecting infectious exposure may be important, particularly at the time of delivery and over the first hours and days of life. In this section we review evidence for the health impact of such practices and program efforts addressing them (Box 2).

**Box 2. Clean Delivery Practices**

- Use of a clean blade for cutting the umbilical cord
- Use of clean cord-tie or clamp
- Handwashing by birth attendant, using soap and water
- Use of clean cloths for wrapping/ covering the baby
- Practices can be facilitated by providing a kit with necessary materials

Clean delivery kits (CDKs) provide basic items for use during delivery for homebirths and typically include items such as: blade and thread, plastic sheet, gloves, cord ties and soap. Programmatically, the kits are often accompanied with training or counseling on use of the kit, hand washing and cord care[269, 270] although there has also been widespread distribution through social marketing programs.

Studies which included counseling or health education on clean delivery and postnatal practices as one element of broader set of behavioral messages, and which did not attempt to disaggregate the effect of hygiene promotion were not retained. For the purposes of this review, we have not included studies focused on hygienic practices of trained traditional birth attendants.

**Evidence:** The available evidence comes largely from studies in which the majority of births were conducted at home. A particular challenge is that although clean practices have been included in a number of “packages” tested, the design of such studies does not allow for disaggregating the contribution of the components comprising the package. The review yielded 15 studies on *clean birth practices*, one of which used newborn mortality as an outcome. There were 17 retained studies on *use of antimicrobial substances* on the cord, skin or in the vagina, 6 of which used newborn mortality as an endpoint. Two studies addressed *hand-washing during the post-natal period*. And there were 9 studies on use of *clean delivery kits*, 3 of which used newborn mortality as an outcome.

**Clean practices at birth:** In a review looking specifically at evidence from African settings, Penfold et al.[271] found reduced risk of newborn death associated with delivery on a clean surface. A large cohort study conducted in Nepal[272] showed lower newborn mortality for births at which the attendants were reported by the mothers as having washed their hands (RR = 0.81, 95% CI: 0.66–0.99). In the Blencowe review[268] discussed below, protective effects of birth attendant *hand-washing* were also seen for neonatal tetanus (4 studies) and cord infection (2 studies). Evidence for other components of clean practices at birth is weaker. The usual study design has been case- control, usually with some effort to control for potential confounding. However the studies have generally lacked statistical power. Most studies reviewed in the Blencowe paper addressing clean perineum, birth surface, instrument for cutting the cord and cord ties have not been able to show an effect for tetanus or sepsis, and none were powered for a mortality effect. Three of 7 studies investigating the use of a clean blade found reduced tetanus risk.

In a more recent cross-sectional study in Uttar Pradesh[273] the association between clean cord care practices at the time of birth (cutting instrument, tie, application of substances other than antiseptics to the cord) and neonatal mortality was assessed, adjusting for a range of potential confounders including: mother’s age, education, religion, caste/tribe, household wealth, and other newborn care practices such as thermal care. Use of an appropriate new or clean cutting instrument was virtually universal. Lower mortality was seen for both use of a *clean cord tie* (RR = 0.70, 95% CI: 0.54–0.91) and *no application of substances other than antiseptics* (RR = 0.70, 95% CI: 0.53–0.91).

**Clean delivery kits:** Blencowe[268] (citing Wkly Epidemiol Rec[274]) reports that “in China, training of traditional birth attendants (TBAs) and providing them with a clean birth kit … led to a reduction in neonatal tetanus rates from 32/1000 in 1948 to 2/1000 in 1961.” Blencowe found 3 studies on clean delivery kits with neonatal mortality as an outcome, all showing a reduction[275–277]. The Meegan study[275] was done in a setting where it was routine to pack the cord-stump with cow dung and is therefore not readily generalizable to other settings. Kapoor[276] documents before-and-after results of an intervention the most important element of which was tetanus toxoid administration, so it does not allow for a disaggregated assessment of the contribution of use of the clean delivery kit. Jokhio[277] reports on results of a cluster RCT in Pakistan, involving 19,557 women. The intervention included the provision of CDKs to existing TBAs and training them on use of CDKs, the 6 “cleans,” and newborn danger signs. Compared to control talukas, the intervention talukas had significantly lower odds of neonatal death (adjusted OR 0.71, 95% CI: 0.62–0.83). A reduction was also found in risk of puerperal sepsis (OR 0.18, 95% CI: 0.14–0.22). Although the study examined maternal mortality, statistical power was insufficient to unequivocally show an effect. Hundley’s 2012 review[278], limited to program approaches which included birth kits, included the same 9 studies considered by Blencowe et al.[268] and comes to similar conclusions. This is also the case for the recent review by Penfold[271].

Published subsequent to, and not included in these systematic reviews, was a study by Seward et al.[269], which examined the association between CDK use and neonatal mortality among home births, using data from the control arms of 3 RCTs conducted in Bangladesh, India and Nepal. In all 3 studies, kits were promoted and distributed through the health care system, although not widely used. Hand-washing was much more common among those using a kit. Lower neonatal mortality was found across the pooled dataset (aOR 0.52, 95% CI: 0.39–0.68). Analysis adjusted for potential confounders including maternal age, education, household assets, number of ANC visits, delivery by health worker, exclusive breast-feeding, fever during the 3 days preceding the delivery and season of birth. When controlling for kit use and potential confounders, associations between specific clean practices and mortality was found for use of: boiled blade (aOR 0.73, 95% CI: 0.59–0.90), antiseptics for cord cleansing (aOR 0.16, 95% CI: 0.04–0.64), use of a boiled tie (aOR 0.71, 95% CI: 0.56–0.90), and a plastic sheet as clean delivery surface (aOR 0.69, 95% CI: 0.51–0.93). With each additional clean practice, the authors found a 16% relative reduction in newborn mortality (OR 0.84, 95% CI: 0.77–0.92). A recently published matched case control study in Pakistan[279] found an increased odds of non-use of CDK comparing tetanus cases with non-cases (aOR 2.0, 95% CI: 1.3–3.1), with adjustment for socio- economic factors.

**Hygiene practices during the postnatal period:** Hygiene practices during the postnatal period addressed in the Blencowe review[268] included use of antimicrobial products on the skin or cord-stump (reviewed earlier in this paper). They also included avoidance of potentially harmful substances for application to the cord, reporting on 2 studies showing elevated risk associated with use of various traditional substances. The review cites evidence from Nepal[272] for lower mortality associated with maternal hand-washing, as reported by the mother at 2 weeks post-delivery (adjusted RR = 0.56, 95% CI: 0.38–0.82). Although adjustment was made in the analysis for a number of potential confounders, residual confounding may still account for at least part of the adjusted effect size.

A RCT in Pakistan[201], using a factorial design, randomized clusters to one of 2 interventions with the control group receiving usual care. All groups had births attended by trained TBAs and received birth kits. One intervention tested was chlorhexidine for cord application (not discussed here). The other intervention was soap plus educational messages given by the TBA promoting hand-washing before handling the baby. The hand-washing promotion was not found to have any effect on mortality (RR = 1.08, 95% CI: 0.79–1.48).

In the face of the relatively sparse evidence available in this area, Blencowe[268] reports on the outcome of a Delphi process with newborn content experts to estimate expected effects of specific clean birth and postnatal care practices, to be used in the LiST tool. The experts estimated that 15% (IQR 10–20) of sepsis- related deaths could be prevented by clean birth practices for home deliveries and 40% (IQR 25–50) by clean practices in the postnatal period.

**Conclusion:** This is an area where, although we have reason to believe that appropriate hygiene practices would be a significant factor in mortality risk, their contribution cannot be easily quantified. Clearly, in any given setting how much reduction in risk of serious infection or death would be achieved depends on how bad things are at baseline. In settings where unhygienic practices and conditions are widespread, one would expect considerably greater impact than in settings where conditions are better.

In the real world, interventions are generally not delivered in isolation. Complementary infection prevention interventions (notably tetatus toxoid and chlorhexidine, addressed elsewhere in this review) further reduce risk, as do other behavioral interventions (e.g. early and exclusive breastfeeding, thermal care). Actual strategy used should be driven by local conditions. For example, clean delivery kits can make a useful contribution under certain conditions.

#### Thermal Care

Newborns have limited ability to regulate their body temperature, particularly in the first twelve hours or so of life[280]. This is particularly so for those born pre-term. Hypothermia is an important risk factor for neonatal death, especially among low-birth-weight infants but also among infants of all birth weights and gestational ages[280]. Hypothermia is also extraordinarily common in infants, regardless where the birth takes place: 32% to 85% of infants born in hospitals have been found to develop hypothermia at some point, while the frequency has been documented from 11% to 92% among babies born at home[281]. As Bissinger notes [282], thermal stress can be critically important especially during the “golden hour” after birth, and especially for very low birthweight newborns. Papers reporting on packages of interventions in which thermal care was one element, but not the particular focus of the intervention or the analysis, were not retained.

**Evidence:** For the purpose of this review our interest is in the possibility that interventions aiming to prevent hypothermia could reduce mortality risk. Such direct evidence is relatively sparse, so this review also considers evidence from descriptive studies which attempt to determine the contribution of hypothermia to subsequent development of serious illness. However, hypothermia can be both a symptom and a contributing cause of life-threatening infection in the newborn. That can pose a challenge in interpreting findings from descriptive studies showing an association between hypothermia and mortality risk.

**Hypothermia—frequency, contributing preventable factors (thermal care):** In Uganda, an RCT in a hospital setting[283], N = 239, found that bathing within one hour of birth was associated with increased hypothermia (<36.5°C rectal temperature) at 70 and 90 minutes postpartum compared with no bathing (OR = 2.90, 95% CI: 1.69–5.04; and OR = 3.88, 95% CI: 2.18–6.91, respectively). In a small observational study at 4 levels of care, Christensson[284] found that about half of newborns were hypothermic; thermal care-practices by midwives were suboptimal. In a large community-based study in Nepal (N = 23,240) with repeated measures of temperature over the first 28 days of life[285], approximately half of newborns were found to have at least one temperature measure of moderate to severe hypothermia (<36.0°C axillary), with the highest likelihood over the first 24–72 hours of life. Risk of hypothermia varied by season but even in the hottest season one in 5 newborns were found to be hypothermic.

**Mortality Risk:** Sodemann[286], in a study in Guinea-Bisseau which used a design allowing population-based effect size to be determined, found that those with temperatures <34.5°C were at 5 times higher mortality risk over the first week of life, with risk persisting to 2 months of age. In a large population-based study in Nepal tracking 23,240 newborns[287], most births had occurred at home. Regular axillary temperature assessments were done (median time to first temperature measurement was 19 hours after birth; 88% were within 72 hours of birth), as well as ambient temperature readings. Analysis was done in a way to reduce the contribution of reverse causality (hypothermia as a sign of potentially severe infection).

Using newly established temperature cut-offs for grading hypothermia (which better correlated with risk), compared with normothermic newborns, risk of death for *first measured temperature* was found to be elevated, as follows:

- Grade 1 (36.0–36.5°C): RR = 1.51 (95% CI: 1.03–2.21)
- Grade 2 (35.0–36.0°C): RR = 1.75 (95% CI: 1.15–2.68)
- Grade 3 (34.0–35.0°C): RR = 5.03 (95% CI: 3.13–8.06)
- Grade 4 (<34.0°C): RR = 9.21 (95% CI: 5.51–15.41)

In this analysis, effect size was adjusted for age, ambient temperature, sex, early breastfeeding initiation, ethnic group, weight, hand-washing practices of mother and birth attendant, place of delivery, and illness on day of measurement.

**Interventions:** We included health facility-based studies, to the extent that they contribute insights relevant to this paper; i.e. relevant to counseling/ health-education content of contacts during pregnancy, with potential to influence care-practices after birth (Box 3).

**Box 3. Thermal Care**

- At birth, immediate drying, placing skin-to-skin on mother’s abdomen or chest, and covering
- Any care requiring that the baby be exposed (e.g. for weighing) should be done under a radiant heat lamp
- Delaying bathing till the second day of life
- Continued skin-to-skin care (especially for very small newborns)

**Reviews:** A Cochrane review by Moore et al[288] of early skin-to-skin care for healthy *full-term newborns* found greater physiologic stability and better breastfeeding outcomes (but the studies included were variable with regard to intervention and comparators). McCall’s Cochrane review[280] on interventions immediately at birth beyond “routine” thermal care for *preterm or low birthweight* newborns found plastic wraps/ caps to be effective for those of gestational age <29 weeks in reducing hypothermia risk; stockinet caps were not.

The Cochrane review of Kangaroo Mother Care by Conde-Agudelo[289] addressed evidence from RCTs of intermittent or continuous skin-to-skin care for *low birthweight* newborns in hospital, generally in comparison to care in an incubator or under a radiant warmer and found lower mortality risk (RR = 0.60, 95% CI: 0.39–0.92, 8 trials). With the exception of one small US-based trial[290] (N = 60), all of these studies with mortality as an outcome were conducted in low and middle-income countries. Although thermal care was a central component of the intervention package tested, there were other elements, notably focused on support for feeding. Findings from these reviews do, however, provide evidence for the effectiveness of extended skin-to-skin care on thermal stability and consequent survival.

**Important individual papers:** In a hospital-based, before-and-after study conducted in Nepal[291] at baseline the usual practice was not to immediately dry and wrap newborns. At 2 hours after birth 85% had rectal temperatures <36°C (N = 495) and at 24 hours after birth, 50% had temperatures that low (N = 405) and 14% had temperatures <35°C. After introducing the new practice of immediate drying and wrapping, at 2 hours after birth 38% had rectal temperatures <36°C (N = 298) and at 24 hours, the proportion with temperatures that low was 18% (N = 231); none had temperatures <35°C.

Bergman[292], in a study of late preterm newborns, showed greater cardio-respiratory stability if they were kept skin-to-skin and blood glucose level 75 to 90 minutes following the birth was significantly higher (compared to those receiving incubator care).

In a RCT in a tertiary care setting in Gujarat including term and late preterm newborns[293], skin-to-skin care was started within 30–60 minutes of delivery and continued for 24 hours. Newborns in the skin-to-skin care group achieved more rapid thermal control compared to controls and significantly lower incidence of hypothermia over first 48 hours. In this study those in the control group had 8 times higher risk of developing hypothermia than those receiving skin-to-skin care (RR = 8.0, 95% CI: 1.9–33.0).

In a community-level cluster RCT in Bangladesh[52], household-level antenatal counseling for all pregnant women encouraged continuous 24 hour per day skin-to-skin care, particularly over the first 2 days of life, regardless of birthweight. The trial showed effects on maternal care practices including: earlier initiation of breastfeeding (although even in the intervention arm this was generally quite delayed, at an average of 5 hours after birth), much reduced bathing within the first day of life, and at least some practice of skin-to-skin care (with 24% of those in the intervention arm reporting more than 7 hours a day of such care). However, no mortality effect was demonstrated. Since overall mortality across both intervention and control arms was lower than assumed for the study power calculations and the sample size was determined to detect a reduction in neonatal mortality of 27.5% or more, the study may have been underpowered to show a mortality effect. Furthermore, while such thermal care practices can be beneficial for normal birthweight babies, it is particularly among those who are low birthweight that one would expect a significant mortality effect. The same investigators did further analysis of data from their original study[294] and found a dose-response relationship with the lowest mortality for those held skin-to-skin at least 7 hours per day over the first 2 days of life; neonatal mortality in this group was just 1/4 of the national rural average. Newborns kept skin-to-skin for less than 7 hours per day did not have better health or survival than infants that those receiving no skin-to-skin care. Although this is suggestive of benefit, an important contribution from uncontrolled confounding cannot be ruled out.

In a somewhat similar community-based cluster RCT in Uttar Pradesh, Kumar[295] showed significantly lower mortality than in their comparison arm (RR = 0.46, 95% CI: 0.35–0.60). This study was conducted in a rural area where most births take place at home. Baseline mortality was very high (84/1000 in the control arm). The intervention focused on prevention of hypothermia (including skin-to-skin care) but also addressed birth preparedness, clean delivery and cord care, breastfeeding, and care-seeking from suitable providers. Home visitation by community health workers during pregnancy and post-natally was an important element. Unlike the Bangladesh study[52], the approach also entailed fairly intensive community mobilization. Although the emphasis was on thermal care, because the actual package delivered was considerably broader it is not possible to factor out its contribution to the observed reduction in mortality risk.

**Conclusion:** Thermal care practices can be seen as part of a broader behavioral cluster also including early initiation/ exclusive breast-feeding and immediate stimulation at birth. Optimal practice of this suite of behaviors is important for all babies especially during the first day of life but especially important for very small babies, extending over the first days and weeks of life. In settings where many births happen at home, it is particularly during pregnancy that there are opportunities to influence such practices at birth and over the first hours afterwards. From data published in Mullany[287], we have evidence that hypothermia is very common and is a potent risk factor for newborn mortality. The causal pathway we have been considering goes as follows:

thermal care practices → hypothermia → serious infection or other pathology → death

We do not have definitive evidence available to confidently tease apart such causal flow from other potentially related factors. In the analysis in Mullany[287], various moves were made to control out “reverse causality”–cases in which hypothermia was in fact a sign accompanying sepsis leading to death—as well as a number of known potential confounders including: place of birth, gestational age, timing of breastfeeding initiation, and handwashing practices of the mother. With such adjustments made in the analysis, large effect sizes remained. For all of those in the sample found to be hypothermic at first temperature measurement, if their risk of death were reduced to that of those who were normothermic (with the analysis controlled for these various potential confounders), overall mortality in the sample would have been reduced by 45%. Clearly, there is more going in this population than what is outlined in the causal pathway above. The association seen between hypothermia and mortality risk may be confounded by other unmeasured factors. Furthermore, thermal care practices are certainly not the only important driver of hypothermia prevalence. So the actual mortality effect of significantly improving thermal care practices would certainly be less than 45%, but these data suggest the effect would nevertheless be quite large. There are simple measures that can be taken over the first minutes and hours of life that can meaningfully reduce thermal stress. For the magnitude of reduction in risk of death that can plausibly be inferred from available evidence, the minimal programmatic attention given to improving such practices is hard to explain. Specifically within the remit of the current paper, contact during pregnancy represents an important—and usually missed—opportunity for influencing thermal practices at and after birth for those happening at home. There is clearly much room for improvement in thermal care practices for institutional deliveries as well, but that falls beyond the scope of the current paper.

#### Birth Preparedness/ Complication Readiness

There has been a focus in Safe Motherhood work, dating from the seminal paper by Thaddeus and Maine[296], on addressing 3 delays to definitive care for complications, the first 2 of which are function of household level recognition, decision-making, and the challenges of getting to a health facility. Program approaches have tended to focus on one or more of the following:

- Awareness/ knowledge addressed through “behavior change communications”, mass, print and multi-channel
- Supporting household level problem solving, typically through counseling by a CHW or health worker, and sometimes using some form of birth planning card to work out specific details of a plan
- Guided community empowerment using techniques like the community action cycle
- Addressing practical barriers e.g. through community transport and financing schemes

By the late 1990s, there were program efforts in a number of countries addressing readiness to respond quickly and appropriately in the event of signs of a serious complication. These included program experiences under MotherCare in the late 1990s in Indonesia, Guatemala, Bolivia and Uganda; other early program experience in Egypt, under a USAID-funded bilateral project, and in Bangladesh under CARE; and further work under the USAID-funded MNH project from around 1999–2000, with large-scale programs in Nepal, Indonesia and Guatemala. Under MotherCare, rubrics like the following were used to describe this area of work, “Improving Decision-Making, Preparation, and Care Seeking for Obstetric Complications.”[297]

It may have been under the USAID-funded Maternal Newborn Health project (MNH) that the term “Birth Preparedness/ Complication Readiness” was first coined to characterize this area of program focus[298]. Similar language is used in WHO guidance documents from the early 2000s (“Birth and emergency preparedness” [299]).

Typically, in programs of this kind the range of content covers:

1. Birth preparedness/ complication readiness (may include use of “birth preparedness card”):
  - determining the attendant and location for birth,
  - working out arrangements for emergency transport,
  - saving for expected expenses and possible complications
  - having any needed materials if expecting a home delivery, e.g. clean delivery kit, clean cloths for drying/wrapping the newborn, and often …
2. Danger sign recognition and response during pregnancy, at birth, and during the postnatal period.

Other content areas that, in some programs, are addressed together with birth preparedness and complication readiness include the following:

- Routine care-seeking for: ANC, tetanus toxoid, institutional delivery
- Essential newborn care practices, especially at birth [see earlier section]:
  - early breastfeeding initiation,
  - thermal care,
  - clean birth practices (CDKs)
- Self-care practices during pregnancy

In this section we will confine our attention to program efforts addressing the 2 areas above (birth preparations and danger sign recognition and response). Note that in the Discussion section, there is a broader discussion on program strategies, including “packages,” which addresses these other areas of program focus. Even confining ourselves to program efforts addressing the first 2 areas, as discussed above, audiences targeted for such work have varied, with some programs focusing specifically on the pregnant woman and other key decision-makers in her household and others focusing mainly on the community.

We retain papers documenting the results of program approaches focusing on birth preparedness/ planning and on recognition and appropriate response in the case of danger signs. We exclude papers reporting on implementation of broader packages in which the birth preparedness/ complication readiness component is not central or is not the subject of disaggregated assessment of contribution. We also exclude studies that document initiatives not specifically targeting pregnant women and their families.

**Evidence:** Soubeiga[300], which restricted its review to RCTs, captures 12 trials: 7 (at 6 South Asian sites) using the participatory women’s group approach pioneered by Costello and colleagues, and 5 other South Asian community trials using pregnancy and postnatal home visits by CHWs, along with community education or community mobilization of varying intensity across sites.

In the participatory women’s group trials, although the content focus included birth preparedness, the programs generally did not specifically target pregnant women (although in the more recent trials more effort was made to encourage participation in the group meetings by pregnant women). The focus in these programs has tended to be on what *communities* can do to help facilitate access to maternal-newborn care, particularly for labor and delivery complications, rather than directly seeking to influence household decision-making.

Across all 7 trials, Soubeiga found a pooled effect size for neonatal mortality of RR = 0.83 (95% CI: 0.70–0.98). Across these studies, there was not a statistically significant reduction in maternal mortality (RR = 0.72 (95% CI: 0.46–1.13) although, considering only the 4 trials in which at least 30% of pregnant women were reached by the women’s groups, the pooled effect size was 0.47 (95% CI: 0.26–0.87). Prost[301] reviewed the same participatory women’s group trials covered in the Soubeiga review; their analysis yields different numbers but essentially the same conclusion. They write: “With all 7 trials the results indicate a 34% reduction in maternal mortality (0.66, 95% CI: 0.39–0.93), and a 20% reduction in neonatal mortality (0.80, 95% CI: 0.68–0.93).”

So, overall, this approach has shown effectiveness but with considerable variation across cases. In the one urban-based study[302], conducted in Mumbai, there was no effect found on newborn mortality (the main trial outcome). Similarly, a first effort in Bangladesh in which there was a comparatively low ratio of group facilitator to population also failed to show an effect[303]. When support was intensified by increasing the number of facilitators, an effect was documented on newborn mortality[304]. In summary, at least under certain conditions, use of the community action cycle approach can reduce maternal and newborn deaths.

As mentioned above, the 5 other community level cRCTs reviewed in Soubeiga tested approaches that included community health worker home visits during pregnancy and after the birth, supplemented to varying degrees of intensity by community level promotion. Results were mixed across these studies, with no effect found on newborn mortality (the main endpoint) in studies in Bangladesh[305] and Ghana [306] but mortality reduction seen in the 3 others[51, 295, 307]. Various factors may have contributed to the different results, notably differences in context and in the details of what was done. Unlike the participatory women’s group studies, in all of the second group of studies, the intervention targeted both the period of pregnancy and postnatal. And the content focus was not restricted to birth preparedness/ complication readiness but included important elements of household level newborn care. So the analysis does not allow for a disaggregated assessment of the contribution of the BP/CR component to the observed results.

For new WHO guidelines on behavioral interventions for maternal-newborn health[308] an evidence review was conducted, which included evidence on program approaches addressing birth preparedness. The review captured some studies not included in the Soubeiga review, including one cRCT[309] and several studies using quasi-experimental or pre-post only designs. The study by Midhet at al.[309], conducted in a setting in Pakistan with low levels of institutional deliveries, included: individual-level birth preparedness and community mobilization elements, but also a transport scheme and training of TBAs in clean delivery and danger sign recognition. The investigators demonstrated a reduction in perinatal mortality, but the design does not allow for disaggregation of the specific contribution of the birth preparedness component. Of the studies using quasi-experimental or pre-post only designs, all showed effects on awareness of danger signs and birth preparations; most showed increases in some aspects of care-seeking (e.g. Brazier [310]), though not all showed increases in institutional delivery. The intervention in Hodgins study in Nepal[311] consisted primarily of CHW counseling for pregnant women, including birth preparedness and essential newborn care; it showed a significant reduction in newborn mortality, although due to the pre-post only design the effect cannot confidently be attributed to the intervention.

Another study captured in the WHO review was done at district-wide scale in Nepal[312] and used a pre-post design with no comparison area. The population in the program area was 615,000. The program addressed birth preparedness/ complication readiness plus essential newborn care and relied primarily on counseling of pregnant women by female community health volunteers who are part of the government health structure.

The program was delivered with modest external support. From a survey with a representative sample of women who had delivered over the previous 12 months, 54% were found to have been reached directly by the program and another 18% indirectly exposed. Compared to baseline, the study found significant improvements in newborn care practices and in scores on a birth preparedness index, but no changes in the institutional delivery rate (17%).

There are a number of other relevant studies and documented pilot projects which were not included in these reviews. A multi-site randomized controlled trial [313–315] in Argentina, Brazil and Cuba (N = 2,235), tested a targeted strategy of home visits for pregnant women considered to be of high psychosocial risk. For each of the participating pregnant women, there were 4 to 6 home visits made by nurses or social workers, the main objective of which was to strengthen the pregnant woman’s social network. Health education was provided on self-care practices and the women were encouraged to make use of ANC. The primary endpoints were low birthweight, preterm birth and several categories of maternal and newborn morbidity. The trial did not show impact on any of these outcomes.

In a quasi-experimental study in Burkina Faso[310] birth preparedness planning was done as part of ANC, supplemented by a community-level campaign through outreach agents, which included community theatre and other activities. The intervention also included significant “supply-side strengthening.” The intervention resulted in big increases in service utilization; institutional deliveries rose from 29% to 57% in the intervention district but remained unchanged in the comparison district. Due to the design of the study it is not possible to disaggregate the effect of the birth preparedness component, but one can conclude that when both supply and demand-side factors are adequately addressed utilization can markedly improve.

A cluster RCT (N = 905) conducted in Tanzania included 16 dispensaries and 23 health workers[316]. Pregnant women in the intervention arm were supported to develop birth plans through routine ANC. This had the effect of doubling the average length of the ANC consultation. The study demonstrated a somewhat higher institutional delivery rate and significantly higher utilization of postnatal care.

**Conclusions:** Clearly, timely and appropriate management of complications during the periods of pregnancy, labor and delivery and postnatally is one of the most important elements, and some would argue *the* most important, in efforts to drive down maternal and newborn deaths and stillbirths. Reducing delays in recognizing a problem and deciding to seek care, and then in actually getting to a health facility where the problem can be addressed can go a long way towards meeting the needed conditions for good outcomes. Program efforts over the past 15–20 years have focused on this need in various ways, typically as part of a broader effort with goals beyond “preparedness.” The documented experiences have consistently shown that knowledge of danger signs can be improved. In most cases, these studies have shown at least a modest effect in increasing service utilization although often there has been no evidence of increased use of *emergency* services. In some cases, particularly when implemented as one element in a broader package, and when implementation support has been comparatively intensive, mortality reduction has been demonstrated.

#### Tobacco & Alcohol

More than 80% of all smokers live in low- and middle-income countries[15]. While smoking among women in low- and middle-income countries is generally low compared to men, there are rising rates of smoking among women in many LMICs; on current trends this is expected to rise from an estimated 9% to 20% in low- and middle-income countries by 2025[317, 318]. The highest rates of smoking during pregnancy are reported for Latin America (e.g. 18% in Uruguay) and Eastern Europe (e.g. 15% in Romania), with low rates (1–3%) in Southeast Asia[318]. *Smokeless* tobacco use is higher than rates of smoking among women in India (5% to 34%) and some African countries (6% to 8%)[318].

Zelner et al[319], reviewing *alcohol* use in pregnancy across multiple surveys, found rates in USA and Canada around 5–15%, with considerably lower levels of high consumption or binge pattern drinking. Higher rates were reported in Europe (20–40%) and Australia (25–30%); 57% in a study in Chile; 47–59% in Russia and 46–73% in Mexico. Comparable data are not generally available for low income countries.

In a comprehensive review of studies mostly from high-income countries[320], comparing women who smoke during pregnancy to those who do not (controlling for confounders), relatie risk effect sizes (RR) were determined as presented in Table 4, below.

**Table 4:** Increased Risk Associated with Smoking in Pregnancy.

Outcome	RR	Notes
Preterm premature rupture of membranes	2–4	Studies comparing women who quit during pregnancy show lower risk than those continuing. Risk increased with amount smoked.
Abruptio	1.4–2.4	
Placenta previa	1.5–3.0	
Preterm delivery	1.2–2.0	Risk most increased for early preterm, i.e. before 33 weeks, with risk reduced to a level similar to non-smokers if quitting during 1st trimester
Stillbirth	14	Dose-response relationship seen
Neonatal mortality	1.2	Dose-dependent effect not clear; risk reduced if woman quits
Perinatal mortality	1.3, 1.2	For cohort and case-control studies, respectively
Low birth weight	1.5–3.5	Weight of babies of smokers on average about 250gm lower than non- smokers, gap increasing with amount smoked

From a cohort study[321] following about 3 million pregnancies (about 80% of births in the US in 1997), the adjusted risk for infant mortality was 40% higher among pregnant smokers (with a dose-response effect seen), much of which was mediated through IUGR. Analysis controlled for race/ethnicity, maternal age, parity, maternal education, number of alcohol drinks consumed per week, and adequacy of prenatal care. Overall 5% of infant deaths were attributable to smoking, in a population in which 13% of pregnant women smoked.

A review of studies addressing *smokeless tobacco* use and adverse pregnancy outcomes[322] found some evidence for increased risk of low birthweight, preterm birth and stillbirth.

Heavy drinking in pregnancy is well known for its long term effects on the offspring. For purposes of the current review, our focus is primarily on mortality outcomes and intermediate states like preterm birth which increase risk of death. In a review by Bailey et al. [323] most fetal deaths attributable to alcohol were reported to be early in pregnancy. There was clearest evidence for such an effect among those having ≥5 drinks per week. Evidence for effects on preterm births was not as strong, although more robust for higher level intake. Patra’s review[324] on dose-response for fetal alcohol exposure reported no evidence for effect on growth restriction up to one alcohol unit per day or for preterm birth up to 1.5 units/day, but with monotonic increases beyond those levels. A review by Henderson[325] on low to moderate alcohol use found no convincing evidence of effects with regard to fetal death, IUGR, preterm birth, growth restriction or fetal alcohol syndrome. A review on tobacco/ alcohol synergies[326] found a more-than-additive effect for preterm birth and low birthweight.

We aimed to include studies that showed the impact of various education and behaviorally-based interventions on birth and neonatal outcomes. While studies based in high-income countries normally fall outside our inclusion criteria, the vast majority of studies captured were conducted in high-income countries and were therefore included to provide insight into existing evidence.

**Evidence:** A recent Cochrane review[317] found that counselling interventions had a significant effect on *smoking* abstinence in late pregnancy compared to usual antenatal care (24 studies; aRR = 1.44, 95% CI 1.19–1.75). The authors also found that risk was reduced for *preterm births* (14 studies; average RR = 0.82, 95% CI: 0.70–0.96) and low birthweight (14 studies; average RR = 0.82, 95% CI: 0.71–0.94). Cognitive-behavioral interventions were most common, though those entailing use of rewards had higher effect sizes. The authors found insufficient evidence to judge whether any type of counselling strategy is more effective than others.

Three studies of smoking cessation counseling support in pregnancy were identified from low and middle- income countries. In an RCT in Argentina, Brazil, Mexico and Cuba (N = 2,235), pregnant women at risk were targeted[314]. Women in the intervention arm received 4–6 psycho-social support home visits by social workers or nurse practitioners, the focus of which included alcohol and tobacco use. However no significant effect was demonstrated for either of these behaviors. In a cluster RCT conducted in Poland (N = 386), 4 midwifery visits were done during pregnancy for those smoking to provide counseling support on smoking cessation. Odds of cessation were higher in the intervention arm, OR = 2.5, 95% CI: 1.8–3.7 [327]. In a quasi-experimental study[328] conducted in South Africa (N = 949), an intervention consisting of self- help quit materials and counseling by midwives and peer counselors was compared to normal ANC care. Significantly higher quit rates were achieved in the intervention cohort, with an absolute difference of 5.3% between cohorts (95% CI: 3.2%–7.4%, p < 0.0001).

A Cochrane review on interventions addressing *alcohol use* in pregnancy[329] found limited evidence suggesting “that psychological and educational interventions may result in … a reduction in alcohol consumption among pregnant women.”

**Conclusion:** Smoking and alcohol use during pregnancy can have important mortality and developmental consequences for the fetus/ newborn. Smoking has widespread negative consequences in pregnancy. Alcohol intake beyond 1 to 1.5 drinks/ day increases risk of stillbirth and preterm birth. As indicated above, although tobacco use by women in most settings of high maternal and newborn mortality is currently comparatively low, rates are rising. To the extent that use of tobacco and alcohol during pregnancy is significant, efforts are warranted to address this problem. Counseling and health education interventions during pregnancy have been shown to be effective for smoking cessation, albeit with modest effects. Psychological and educational interventions may result in increased abstinence from alcohol[329]. Broader strategies are also warranted, addressing advertising, taxation, packaging, and second-hand smoke exposure.

#### Family Planning

Family planning is relevant for ANC counseling/ health education for several reasons. ANC provides access to women who may not otherwise make regular use of health services. Informing and encouraging women to plan for a suitable interval of time before a subsequent pregnancy, if effective, can contribute to better health outcomes for the woman and her offspring. For adoption of longer-term or permanent methods administered at the time of delivery (tubal ligation, IUCD insertion), counseling needs to be done during pregnancy.

Included were articles focused on contraceptive prevalence and counseling during pregnancy and articles that connect birth spacing or family planning with outcomes of interest including fetal loss, infant mortality and maternal mortality via modeling or observation.

**Inter-Pregnancy Intervals and Outcomes of Interest:** Kozuki[330] conducted a review/ meta-analysis based on 5 cohort studies (3 from the Brazilian Pelotas study [331–333], data from a vitamin A study in Zimbabwe[120], and a Filipino study of intrauterine growth restriction[334]), controlling for confounders. They found short birth interval (<18 months) associated with higher risk of SGA (pooled aOR = 1.51, 95% CI: 1.31–1.75), preterm birth (pooled aOR = 1.58, 95% CI: 1.19–2.10), and infant death (pooled aOR = 1.83, 95% CI: 1.19–2.81). In another review published at the same time[335] meta-analysis was done based on 47 DHS surveys with results similar overall to the review above. However, in analysis stratifying by mother’s parity, the association between short birth interval and higher child mortality was greatly attenuated at low birth order.

An earlier pooled analysis from DHS data[336]– 52 DHS surveys conducted between 2000 and 2005 –included over 1 million births. With preceding birth-to-conception interval of <6months, there was a higher likelihood of low birthweight. Similar findings were seen for stunting. Under-5 mortality was higher with birth-to-pregnancy interval <36 months, with highest risk for the shortest interval, with a doubling of risk (RR = 2.2) for birth-to-pregnancy interval of <6 months, compared to the reference interval of 36–47 months. Similar findings were seen for infant mortality. The author reported that the population-attributable risk for under-5 mortality for avoiding birth-to-conception intervals of <24 months was 0.134.

Another recent large review[337] found significant correlations between short inter-pregnancy intervals and birth outcomes (25 studies, predominantly in middle- and high-income countries). An increased risk was found for stillbirth with inter-pregnancy interval of <7 months (compared to 27–50 months) of pOR = 1.35 (95% CI: 1.07–1.71, 3 studies including Conde-Agudelo[338] and Da Vanzo[339]). Similarly, they found a pooled effect size of OR = 1.29 (95% CI: 1.02–1.64, 3 studies) for death within 7 days of birth.

A systematic review and meta-analysis by Conde-Agudelo[340] found that, compared with inter-pregnancy intervals of 18 to 23 months, intervals <6 months were associated with increased risks of preterm birth (pOR = 1.40, 95% CI: 1.24–1.58), low birth weight (pOR = 1.61, 95% CI: 1.39–1.86), and small for gestational age (pOR = 1.26, 95% CI: 1.18–1.33).

DaVanzo[339], a large observational study in Matlab, Bangladesh, was based on data of from 67,000 mothers and newborns. For births with a live birth initiating the preceding birth interval, the authors found a 3.3-fold increase in the odds of a miscarriage (95% CI: 2.8–3.9) and a 1.6-fold increase in the odds of a stillbirth (95% CI: 1.2–2.1) compared with a 27- to 50-month inter-pregnancy interval.

A large study from Latin America[341] including almost 500,000 births found, after adjustment for major confounding factors, higher risk for maternal death for women with an inter-pregnancy interval of <6 months (OR = 2.54, 95% CI: 1.22–5.38) compared with those conceiving at 18 to 23 months after a previous birth. In subsequent analysis from the same multi-country Latin American database[338], data from over 1 million births were used, finding—in comparison with inter-pregnancy intervals of 18–23 months—higher risk for those of intervals <6 months as follows: early neonatal death—aOR = 1.49 (95% CI: 1.06–1.96), small for gestational age—aOR = 1.30 (95% CI: 1.25–1.36), and preterm birth—aOR = 1.80 (95% CI: 1.71–1.89).

**Counseling and Contraceptive Uptake/Prevalence:** A review by Lopez[342] included quasi-experimental studies of educational or counseling interventions to increase postpartum family planning use. Only 2 of the 6 studies included antenatal counseling (Abdel- Tawab[343], Sebastian[344]). Abdel-Tawab[343] reports on a quasi-experimental study in Egypt testing 2 different approaches: model I delivering post-partum family planning messages in ANC and PNC at facilities and model II also including a community component. In comparison with the control arm, those in the 2 intervention groups were more likely to use contraception at 10 to 11 months postpartum (48% and 43% versus 31% among controls).

In another quasi-experimental study, conducted in India by Sebastien[344], government community workers (Accredited Social Health Activists and Anganwadi workers) who already have an established role providing services to pregnant women, counseled pregnant women (N = 477) and their mothers-in-law or oldest female household member on healthy timing and spacing, postpartum contraception and related topics. This was complemented by other activities focusing on husbands and other community members. Pregnant women in the comparison area (N = 482) were not exposed to the campaign. Most women delivering in the intervention area reported having been exposed to at least some aspects of the campaign. In both intervention and comparison areas most recently-delivering women reported having received counseling on family planning but the proportion was markedly higher in the intervention area (among those 9 months postpartum, the rate was 93% in the intervention area, 69% in the comparison area, p<0.01) and reports on having received counseling on postpartum checkups, lactational amenorrhea, STIs and HIV/AIDS were much more frequent in the intervention than in the control arm. At 9 months postpartum, the proportion using modern family planning was significantly higher in the intervention than in the comparison arm (57% vs 30%, p<0.01).

Smith[345] conducted a randomized trial of antenatal FP counseling in Scotland, China and South Africa (N = 1637). At one year after birth, there was no difference in contraceptive use between intervention and controls (with contraceptive prevalence over 79% in all centers). With such high background rates of use, it is not clear the study was adequately powered to detect an effect. A randomized controlled trial (N = 200) in Egypt tested an antenatal education intervention and demonstrated significantly higher post-partum family planning use, 91% vs 43% in the comparison arm[346]. A pre-/post-intervention study in Honduras[347] included ANC counselling on family planning and wider method choice; it resulted in an increase in uptake of sterilization, IUD and oral contraceptives from 8.8% at baseline to 41.2% at endline. In a randomized controlled trial (N = 216) conducted in Nigeria[348] participants were randomized to receive either several one-on-one antenatal counseling sessions on family planning or a single one-on-one session at the time of their 6-week post-partum follow-up, as was routinely practiced in that center. At 6 months post- partum, use of a modern contraceptive method was significantly higher in the antenatal counseling arm (57% vs 35% in the control arm, p = 0.002). In an under-powered randomized controlled trial conducted in Turkey[349], 60 pregnant women were randomized to receive a single 30-minute one-on-one antenatal counseling on family planning, and 120 to the control group, members of which were given an educational pamphlet on family planning. At follow-up 6–9 months postpartum, although contraceptive use was higher in the counseling than in the pamphlet arm (86% vs 76%) the difference was not statistically significant.

**Conclusion:** Short preceding inter-pregnancy interval is associated with poorer birth outcomes. One author [336], based on analysis of 52 DHS surveys, estimates that 13.4% of under-5 deaths could be averted if there were no births with an inter-pregnancy interval <24 months. As with almost all interventions, evidence for an effect on maternal mortality is sparser, though a large study in Latin America[341] found a 2½ fold higher risk of maternal death with a preceding inter-pregnancy interval of <6 months, compared to those delivering after an 18–24 month interval.

To close the gap for unmet need for birth spacing, clearly more is needed than counseling during pregnancy. However, there is documented program experience from a wide variety of settings for increased rates of postpartum family planning achieved through antenatal counseling. There have been successful experiences using community health workers or health auxiliaries involved in providing services to pregnant women[344].

#### Obstetrical and Other Interventions: Summary

As illustrated in Table 5 (below), several of the interventions considered in this review can be expected to significantly reduce the burden of important outcomes we have considered. Efforts to much more systematically identify pre-eclampsia cases early and to ensure timely delivery can substantially reduce both maternal and newborn mortality. Thermal care of the newborn, especially on the day of birth is deficient in many settings. By better ensuring good thermal care practices a substantial reduction in newborn mortality is achievable, particularly in settings where such rates are high. In settings where many births still occur at home and definitive emergency obstetrical care is not readily available, advance distribution of misoprostol for use immediately after birth can very substantially reduce the risk of life-threatening post-partum hemorrhage.

**Table 5:** Summary of Effects of Obstetrical and Other Interventions on Outcomes of Interest.

Intervention	Effects on:						Comments
	Maternal mortality	Newborn mortality	Stillbirth/ miscarriage	Preterm	IUGR/ LBWt	Other outcomes	
**Misoprostol** (advance distribution)	~50%↓ in PPH-specific mortality in populations with poor access to definitive care [233, 234, 235, 236]						Effectiveness estimate assumes 100% coverage.
**Pregnancy-induced hypertension screening & follow-up**	90%+↓ in eclampsia/ pre-eclampsia-specific mortality, if functional referral and case-management are available [262]	↓ by close to ¼? (PIH is 1° obstetrical cause of ~¼ of perinatal deaths) [255]	↓ by close to ¼? (PIH is 1° obstetrical cause of ~¼ of stillbirths) [255]				
**Clean delivery**		↓					
**Thermal care**		↓, evidence suggestive of large magnitude effect in high mortality settings, 30%+? [287]					
**Birth preparedness**	↓[300]	↓[300]	↓				
**Tobacco, alcohol**		↓	↓	↓	↓		[320, 323]
**FP counseling**	↓, sparse evidence for effect size	↓				Up to ~13% of under-5 deaths avertable by avoiding short birth interval [336]	

## Discussion

In this paper, “interventions” selected largely fall into the categories of: 1) behavioral and 2) clinical preventive, with the second category subdividing into *screening* (and subsequent case-management or referral), and *universal dosing*. Most of the interventions address nutritional, infectious or obstetrical issues. Although interventions retained can be delivered during pregnancy, some are put into operation at birth or later on and, so, are not normally considered part of “antenatal care.” The rationale for including these is that the paper is intended to address circumstances still prevailing in many high mortality burden settings, where many births still occur at home without care from a health professional, and therefore contacts during pregnancy present an important opportunity to influence practices at or soon after childbirth.

The interventions were selected as potentially deliverable through the most peripheral level of the primary healthcare system in low income settings. So those requiring laboratory services beyond simple, currently- available, point-of-service diagnostics or requiring competencies beyond those of a health auxiliary (like an auxiliary nurse-midwife) have been excluded. Where greater capacity is available, more can be done. And as progress is made in the development of simple, inexpensive, point-of-use diagnostics, other interventions would meet the criteria for this review. For example screening and treatment of vaginitis/ vaginosis has been demonstrated effective in reducing preterm birth[350], so as simple, inexpensive test kits with adequate test performance characteristics become available, they could be appropriately deployed at the most peripheral level. Furthermore, there are various routine antenatal functions not included here that are nevertheless important in ensuring good pregnancy outcomes (e.g. determining gestational age, assessing for twins).

How much can the interventions considered here contribute to reducing risk of death? As we have seen in this review (and as summarized in Table 5, below), a few of them can contribute a lot in many circumstances; they can be candidates for a universal core package (e.g. pre-eclampsia screening and case management). Some of them, in at least some circumstances, can contribute a lot; in such situations, they should be prioritized (e.g. ITNs and IPTp in highly malarious settings). Some of them, though they may have other benefits, do not appear to significantly reduce risk of our key outcomes of interest (e.g. deworming).

In addition to expected impact, prioritization of interventions or program strategies needs to be driven by how feasible they are in the particular setting for which their use is being considered. In Table 6 below, we have offered very brief comments on such implementation considerations.

**Table 6:** “Best Buy” Ratings for Interventions Reviewed.

Intervention	Population Impact	Ease of Delivery	Comments
Breastfeeding—early initiation	**✓**	☺	Large magnitude effect on newborn mortality
Iron/ anemia	**✓✓✓**	☺	Likely a significant mortality effect, but evidence is weak; clear benefit however for IUGR
*Multi-micronutrient supplementation*	**✓**	☺	*Although IUGR is reduced*, *in many low income settings there may be no net benefit; under some conditions there may be net harm*
Calcium	**✓✓✓**	**😐**	Large magnitude effects but significant logistical barriers at currently recommended dose
*Antenatal vitamin A*		☺	*No effects on our outcomes of interest*
Postnatal vitamin A	**✓**	☺	Evidence for impact on early infant deaths in vitamin-A deficient populations
Iodine	**✓**	☺	Major benefit in ↓’d burden of cognitive disability
Balanced protein-energy supplementation	**✓✓✓**	**😐**	Significant benefits, but cost & logistical challenges limit scalability
*Deworming*		☺	*No effects on our outcomes of interest*
IPTp/ITNs	**✓✓✓✓**	☺	Large magnitude effects in malarious areas
Tetanus toxoid	**✓**	☺	In most settings, coverage is now relatively high; this needs to be sustained
Chlorhexidine advance distribution	**✓**	☺	Large magnitude effect on newborn mortality in high NMR populations
Syphilis screening & treatment	**✓✓✓**	☺	Relatively inexpensive and straightforward diagnostics and treatment
PMTCT	**✓**	**😐**	High population effective coverage requires robust services and systems
Misoprostol advance distribution	**✓**	☺	To ensure safety, delivery strategy needs to reliably ensure counseling on correct timing of use (i.e. immediately after birth)
Pregnancy-induced hypertension screening & follow-up	**✓✓✓**	**😐**	Large magnitude effects but requires robust referral linkages between 1° level and hospital
Clean delivery	**✓**	☺	Magnitude of effect not readily determinable
Thermal care	**✓**	☺	Large magnitude of effect for newborn mortality
Birth preparedness	**✓✓✓**	☺	Magnitude of effect not readily determinable
Tobacco, alcohol	**✓✓✓✓**	☺	In populations where use is common in pregnancy, effectively ↓’ing this has large magnitude effects
FP counseling	**✓✓✓**	☺	Magnitude of effect not readily determinable

Notes on table:

• The number of **✓**’s indicates the number of outcomes of interest (maternal mortality, newborn mortality, etc.) for which there is evidence of an expected impact of ≥10% in at least some settings.

• Ratings of “ease of delivery” are based on costs, logistical and other systems demands, and are categorized: relatively easy ☺, more challenging **😐**.

• For interventions indicated in *italics*, evidence from this review suggests no net benefits on our outcomes of interest.

As has been commented repeatedly through this review, for most of the interventions considered, it is not meaningful to speak of a pure universal effect size. The effect size in a particular study only represents the intervention effects in that particular setting under the specific conditions of the study. As such, pooling such results from several studies cannot be counted on to yield a pure, context-free effect size. Generally, single interventions work in interaction with other interventions, contributing to an overall effect; we may oversimplify by trying to isolate an effect size. The overall effect in a particular setting is a function of: “intervention” plus support factors plus other factors which—together—yield a complete, sufficient set of necessary conditions [16, 351, 352]. The actual contribution will depend in part on what other interventions are delivered; e.g. if quality case-management of complications is reliably available, the contribution of an otherwise effective corresponding preventive intervention to mortality reduction may be quite modest.

More emphasis is needed on taking advantage of opportunities during pregnancy to improve outcomes. Maternal-newborn program work has tended to heavily prioritize the time around childbirth. Although clearly this is a vitally important point in time, as results of this review demonstrate there are important missed opportunities for improving outcomes if interventions delivered during pregnancy are neglected. For example, historical analysis by Goldenberg reveals that most of the decline in eclampsia/pre-eclampsia- related mortality in high income countries over the past 80 years can be attributed to reliable early detection and timely delivery. A much smaller proportion of the decline can be attributed to better management of cases that had reached a life-threatening state.

For antenatal program effort, a shift is needed from an almost exclusive focus on contact (number of ANC visits), to an emphasis on actual content delivered [5]. This review has sought to cast light on the role such specific content can play in reducing the burden of bad outcomes. These interventions have been selected to be easy, in the sense that they are inexpensive and have modest demands with regard to human resources and technology, so in principle wherever they can potentially contribute a lot, we should strive for high effective coverage.

A broader conception is needed than conventional ANC. There is other content delivered during pregnancy that the maternal-newborn communities have tended to see as falling under other technical programs (e.g. HIV, malaria, nutrition, immunization), which can be quite potent with regard to improving important pregnancy outcomes.

For populations in which many births still happen at home, program or service delivery contacts during pregnancy offer an opportunity to influence practices at the time of childbirth and in the hours and days that follow. Such contacts—whether through regular ANC visits, during outreach services, with CHWs or other program activities—should be better exploited to increase effective coverage for key content.

### Delivery Approaches for Effectiveness and Feasibility

While some specific interventions are widely applicable, optimal strategies for delivering content will depend on characteristics of the setting. In recent global literature on maternal-newborn health, the concept of “package” has often been used as a conceptually useful way to organize diverse topics (e.g. interventions during pregnancy at household and community levels). However, as we have noted, *how* services or programs are delivered needs to be driven by the actual situation on the ground, which can differ substantially from one place to another. Furthermore, reaching specific disadvantaged populations may require special localized approaches. So, globally prescribing the use of particular standardized “packages” or service delivery approaches risks yielding less than optimal program effectiveness.

Only in exceptional cases will the best choice be a single-intervention, vertical delivery strategy; in most cases a particular delivery channel or contact is best used to pursue more than one objective and to deliver more than one intervention.

Of the interventions reviewed here, several were behavioral (thermal care practices, breastfeeding, birth preparedness, etc.). Several studies have been reviewed in this paper which lumped a number of components together. This complicates interpretation of the study result, since the methodologies used generally preclude disaggregating the contributions of individual components. However, in program work it can make sense to address “behavioral suites.” There are some natural groupings, by timing and setting. For example, where many deliveries happen at home, there is a set of key practices immediately at the time of birth: 1) clean delivery care & gentle stimulation (note that LiST models an effect for “immediate assessment and stimulation” 0.1 for asphyxia mortality risk, and 0.1 for preterm-attributable mortality risk[353]);2) thermal care; and 3) early initiation of breastfeeding, for which this review has found evidence of effectiveness, that—in program work—would generally be best approached as a bundle or package. Depending on the setting, this could be supplemented by clinical preventive interventions that can be put into operation at the time of home births, notably the use of misoprostol for prevention of PPH, application of chlorhexidine to the umbilical cord stump and newborn vitamin A supplementation.

A number of interventions considered here would be delivered as counseling or health education during pregnancy. Depending on program setting, it may be appropriate to bundle such content.

Depending on the setting there may be a variety of available channels to support and influence pregnant women. These issues can certainly be addressed (though often are not) during regular ANC visits. They can also be delivered by community health workers or health auxiliaries in individual or group counseling sessions.

As was discussed in the section on Birth Preparedness, in programs of this type the range of content can cover:

1. Birth preparedness/ complication readiness
2. Danger sign recognition and response during pregnancy, at birth, and during the postnatal period.
3. Routine care-seeking for: ANC, TT, institutional delivery
4. Essential newborn care practices, especially at birth
5. Self-care practices during pregnancy

In a number of settings there have been trials or demonstration projects and, in some settings, programs implemented at scale using *community health workers*[311]. Much of this content can also, in principle, be included in antenatal care provided by professional health workers (although see von Both[354] on actual time available for ANC counseling). Although individual counseling is the most common strategy, there has also been experience with group teaching and use of the community action cycle or other community mobilization approaches, as well as mass and other media communication strategies. In some programs there are associated community-level transport and funding schemes. Potential impact of such program effort is a function of: 1) the effectiveness of the approaches used in influencing these practices (counseling, health education, community mobilization, etc.), and 2) the etiologic fraction associated with the particular practice or intervention promoted which, in turn, is a function of the effectiveness of the intervention and what proportion of deaths in a particular population are a result of causes addressed by that intervention. So, for example, counseling women during pregnancy to sleep under an ITN may or may not increase her likelihood of adopting the practice; impact will also depend on the efficacy of ITN use and the background malaria risk in that setting.

As has been noted throughout this paper, although there are certain interventions that can be recommended for virtually all settings, in many instances what interventions are prioritized and how they are delivered should be driven by contextual considerations. Certainly, epidemiology will often be a key consideration. All things being equal, interventions that effectively address conditions accounting for high population disease burden should be prioritized. But equally important is what is available for delivery of such content. If an intervention has systems requirements that cannot readily met in a given setting, in general that intervention should not be prioritized unless or until such conditions can be met. In Table 6 (below) for each of the interventions reviewed we have briefly outlined a number of potentially important considerations.

Delivery strategies selected in any particular setting should be determined in part by what platforms are available (see Table 7, below). An obvious one is formal antenatal care. In many countries, although the content delivered through ANC is quite inadequate, contacts *are* happening. That represents an important, if not fully exploited, opportunity. In some settings, community health workers are active and involved in related services. Depending on the circumstances, they may be a good means for providing at least some of the needed interventions during pregnancy. One important criterion for inclusion of interventions in this review has been that they are simple enough not to require professional health workers. So, in principle, virtually all of the interventions considered here could be delivered by community health workers.

**Table 7:** Contextual Considerations.

Intervention	
Breastfeeding—early initiation	Will require different strategies for home births and facility births. High impact and, in principle, easy but tends not to get sufficient attention. Should be prioritized in all settings.
Iron/ anemia	To be recommended in virtually all settings. In principle easy but—inappropriately—tends not to be adequately prioritized.
Multi-micronutrient supplementation	Some evidence to suggest that such supplementation should only be done in settings where births are happening in high quality health facilities with comprehensive emergency obstetrical care capability.
Calcium	Significant expected benefit in calcium-deficient populations. But current recommended dose imposes important logistical difficulties.
Antenatal vitamin A	Routine supplementation not indicated but in vitamin A deficient populations screening for night blindness and treating such cases with vitamin A can be recommended.
Postnatal vitamin A	Benefit expected in vitamin A deficient populations (e.g. in Asian settings where this has been tested and shown impact in early infant mortality).
Iodine	In settings where at least some segments of the population use un-iodized salt, active measures are needed to support universal use of iodized product.
Balanced protein-energy supplementation	Although of significant benefit for malnourished pregnant women, due to cost and logistical challenges, in general delivery of this intervention will only be feasible under special conditions.
Deworming	In settings with high hookworm burden, there may be reasons to sustain this practice however our review of evidence does not show impact on the outcomes considered.
IPTp/ITNs	In heavily malarious areas, can have large magnitude effects but coverage is low in many such settings. Requires more effective program attention, e.g. not simply doing population ITN distribution but making efforts to ensure use by pregnant women.
Tetanus toxoid	In most settings this is being delivered at high coverage. Where it is not, special efforts are warranted. Where high coverage has been achieved, it needs to be sustained.
Chlorhexidine advance distribution	Advance distribution is especially appropriate where a significant proportion of births still happen at home. Magnitude of effect can be expected to be proportionate to levels of NMR.
Syphilis screening & treatment	In populations where syphilis prevalence is relatively high, magnitude of benefit can be large. In low prevalence settings, with very limited resources, it may be appropriate to deprioritize.
PMTCT	An important intervention in settings with high HIV prevalence.
Misoprostol advance distribution	In settings where a significant proportion of births still happen at home and where access to good quality emergency obstetrical care is a problem, this should be prioritized.
Pregnancy-induced hypertension screening & follow-up	In all settings this is expected to have large magnitude effects. However, effectively providing such a service requires strengthening referral linkages between primary and hospital levels; in some settings this will be challenging.
Clean delivery	Needed everywhere. In principle, easy. In settings where this remains a significant problem it needs priority attention.
Thermal care	Important in all settings. High impact and, in principle, easy but tends not to get sufficient attention. Should be prioritized in all settings.
Birth preparedness	In many settings, counseling/ health education for pregnant women gets short shrift. Strategies will depend on available delivery platforms (ANC, CHWs, pregnant women’s groups, etc.).
Tobacco, alcohol	In many low-income, high burden settings population etiologic fraction attributable to tobacco and alcohol use may be relatively small. But where use is common and expected etiologic fraction is high, this should receive priority attention.
Family planning counseling	In populations in which short birth interval is common, emphasis on FP counseling in pregnancy will be appropriate.

In cases where there is an existing program targeting pregnant women which is achieving high coverage, it may be possible to effectively piggy-back other interventions. For example, if the immunization program is doing a good job reaching pregnant women with tetanus toxoid, perhaps this implementation platform can be used to carry other interventions. Similarly, in settings that are achieving high coverage with iron-folate supplementation, there could be promise in making use of the same supply-chain and delivery strategies to provide other supplements (like calcium).

## Conclusion

Antenatal care has tended to be dismissed as relatively unimportant to maternal and newborn outcomes. And, it has tended to be seen as an amorphous package. Program performance has been tracked in terms of contacts, with the ANC4+ indicator being the main performance measure used. Often this has been operationalized specifying that the contact needs to be with a “skilled birth attendant”, i.e. a health worker with the full set of midwifery competencies (excluding services given by other categories of health worker or health auxiliary). But the content of care has not received equal attention from the maternal-newborn community. Instead, it is other programs (malaria, nutrition, immunization, HIV, etc.) that have given attention to particular interventions provided during pregnancy. Such neglect of content represents an important missed opportunity to achieve better population maternal-newborn outcomes. Optimal strategy for delivering such content will depend on context. But with more serious attention to ensuring provision of an appropriate set of interventions at high coverage, particularly among disadvantaged populations, substantially reduced mortality is achievable.

## Supporting Information

S1 File Search Syntax. (DOCX)

S1 Note Additional Methodologic Notes. (DOCX)
