# Supplementary material for: A New Look at Care in Pregnancy: Simple, Effective Interventions for Neglected Populations
Source: PLoS One. 2016 Aug 18;11(8):e0160562. doi: 10.1371/journal.pone.0160562 (PMC4990268; doi:10.1371/journal.pone.0160562)
Supplement: S1 File — (DOCX) [file pone.0160562.s001.docx]

**S1: Search Syntax**

**Pubmed**

**Additional “OR”s added on 1/30/14 are marked in **red**. British-spelling versions of existing search terms are marked in **blue.** Moved iodine terms, now in correct concept 4, marked in **green****

**ALL RESULTS SHOULD BE LIMITED TO THE PAST 30 YEARS**

Filters activated: Publication date from 1982/01/01 to 2012/07/31, English, human-only

**SETS**

Set 1: Screen and Treat

Antenatal terms (Concept 1)

AND

Conditions (“Blood Pressure” et. al) (Concept 2)

AND

Screen Terminology (Concept 3)

RESULTS= 6,284

Set 2: Universal Dosing

Antenatal terms (Concept 1)

AND

Universal Dosing (“Calcium” et. al) (Concept 4)

AND

LMIC List (Concept 6)

RESULTS 3,671

Set 3: Behavioral/Counseling

Antenatal terms (Concept 1)

AND

Behavioral (“Antenatal family planning” et. al) (Concept 5)

AND

LMIC List (Concept 6)

5,455

**Concept 1: antenatal**

“Antenatal”[tw] OR “prenatal”[tw] OR “perinatal”[tw] OR “neonate”[tw] OR “neonates”[tw] OR “neonatal”[tw] OR “Newborn Infant”[tw] OR “Newborn Infants”[tw] OR “neonate”[tw] OR “neonates”[tw] OR “newborn”[tw] OR “newborns”[tw] OR "Infant"[Mesh] OR “Infant, Newborn”[mesh] OR “infant”[tw] OR “infants”[tw] OR “neonate”[tw] OR “newborn”[tw] OR “baby”[tw] OR “babies”[tw] OR "Premature Birth"[Mesh] OR “Premature Birth”[tw] OR “Premature Births”[tw] OR “Preterm Births”[tw] **OR** “Preterm Birth”[tw] OR "Infant, Premature"[Mesh] OR “Premature Infant”[tw] OR “Premature Infants”[tw] OR “Premature Infant”[tw] OR “Prematurity”[tw] OR "Obstetric Labor, Premature"[Mesh] OR “Premature Labor”[tw] OR “Preterm Labor”[tw] OR “Premature Obstetric Labor”[tw] OR "Stillbirth"[Mesh] OR "Stillbirth"[tw] OR "Stillbirths"[tw] OR “fetal death”[tw] OR “Gestation”[tw] OR "Infant, Low Birth Weight"[Mesh] OR “Low Birth Weights”[tw] OR “Low Birth Weight”[tw] OR "Infant, Very Low Birth Weight"[Mesh] OR "Infant, Extremely Low Birth Weight"[Mesh] OR “Omphalitis”[tw] OR “postnatal”[tw] OR “post partum”[tw] OR “postpartum”[tw] OR “post partum”[tw] OR "Postnatal Care"[Mesh] OR "Postpartum Period"[Mesh] **OR "Premature Labour"[tw] OR "Preterm Labour"[tw] OR "Premature Obstetric Labour"[tw] OR “foetal death”[tw]**

**Concept 2: Interventions and Diseases**

“Blood pressure”[tw] OR "Blood Pressure"[Mesh] OR "Proteinuria"[Mesh] OR “proteinuria”[tw] OR “GU infection”[tw] OR “GenitoUrinary tract infection”[tw] OR “GenitoUrinary infection”[tw] OR “GenitoUrinary infection”[tw] OR "Urinary Tract Infections"[Mesh] OR "Urinary Tract Infections"[tw] OR “urinary tract infection”[tw] OR “urinary infection”[tw] OR “urinary infections”[tw] OR "Bacteriuria"[Mesh] OR "Bacteriuria"[tw] OR “Pyuria"[Mesh] OR “Pyuria"[tw] OR “Pyurias”[tw] OR "Vaginosis, Bacterial"[Mesh] OR “bacterial vaginosis”[tw] OR “Bacterial Vaginoses”[tw] OR “Bacterial Vaginitis”[tw] OR “genital tract infection”[tw] OR “genital tract infections”[tw] OR "reproductive tract infections"[MeSH Terms] OR "reproductive tract infections"[tw] OR “Reproductive Tract Infection”[tw] OR “Genital Tract Infections”[tw] OR “Genital Tract Infection”[tw] OR "Syphilis"[Mesh] OR “Syphilis”[tw] OR “anemia”[tw] OR “anemia”[mesh] OR Anemias OR "Anemia, Iron-Deficiency"[Mesh] OR “Transplacental Exposure"[tw] OR "Maternal-Fetal Exchange"[Mesh] OR "Maternal-Fetal Exchange"[tw] OR “Mother to child transmission”[tw] OR “MTCT”[tw] OR “prevention of mother-to-child transmission”[tw] OR “PMTCT”[tw] OR “prevention of parent-to-child transmission”[tw] OR “PPTCT”[tw] OR “HIV infected pregnant women”[tw] OR “maternal transmission”[tw] OR “transplacental transmission”[tw] **OR "Maternal-Foetal Exchange"[tw]**

**Concept 3: screening**

screen*[tw] OR "Neonatal Screening"[Mesh] OR "Mass Screening"[Mesh] OR "Prenatal Diagnosis"[Mesh] **OR “antenatal diagnosis”[tw]**

**Concept 4: Universal Dosing**

"Calcium"[Mesh] OR “Deworming”[tw] OR “IPTp”[tw] OR “intermittent preventive treatment”[tw] OR "Iron, Dietary"[Mesh] OR “iron supplements”[tw] OR “iron supplementation”[tw] OR “iron supplement”[tw] OR “iron folate supplements”[tw] OR “iron folate supplement”[tw] OR “Iron folate supplementation”[tw] OR “iron acid supplement”[tw] OR “iron acid supplements”[tw] OR “iron acid supplementation”[tw] OR "Diet Therapy"[Mesh] OR “therapeutic diets”[tw] OR "Nutritional Support"[Mesh] OR "Maternal Nutritional Physiological Phenomena"[Mesh] OR “Maternal diet”[tw] or “maternal nutritional supplementation”[tw] OR “maternal nutrition”[tw] OR “Micronutrient supplementation”[tw] OR "Tetanus Toxoid"[Mesh] OR “Tetanus toxoid”[tw] OR “Chlorhexidine”[tw] OR "Chlorhexidine"[Mesh] OR "Misoprostol"[Mesh] OR “misoprostol”[tw] OR “Retinol”[tw] OR “anthelmintic”[tw] OR “Antihelmintics”[tw] OR “Vermifuges”[tw] OR "Anthelmintics"[Mesh] OR "Albendazole"[Mesh] OR “albendazole”[tw] OR "Mebendazole"[Mesh] OR “mebendazole”[tw] OR "Praziquantel"[Mesh] OR "Praziquantel"[tw] OR "beta Carotene"[Mesh] OR “beta carotene”[tw] OR “Betacarotene”[tw] OR "Vitamin A"[Mesh] OR “vitamin A”[tw] OR "Tetanus Toxoid"[Mesh] OR “Tetanus Vaccine”[tw] OR “TT vaccine”[tw] OR “TT immunization”[tw] OR "Diphtheria-Tetanus-acellular Pertussis Vaccines"[Mesh] OR “Diphtheria Tetanus acellular Pertussis Vaccines”[tw] OR "Diphtheria-Tetanus-Pertussis Vaccine"[Mesh] OR "Diphtheria-Tetanus Vaccine"[Mesh] OR “Diphtheria Tetanus Vaccine”[tw] OR “DPT” [tw] OR “TDAP”[tw] OR **"Iodine"[Mesh] OR "Iodine"[tw] OR "Iodides"[Mesh] OR "iodized oils"[tw] OR "iodides"[tw] OR "iodine-deficiency"[tw]**

**Concept 5: Behavioral/Counseling**

“Antenatal family planning”[tw] OR “Antenatal family planning counseling”[tw] OR “voluntary counseling and testing”[tw] OR “VCTC”[tw] OR “integrated counseling and testing”[tw] OR “ICTC”[tw] OR “Birth-preparedness Programmes”[tw] OR “birth-preparedness package”[tw] OR “Birth-preparedness Programs”[tw] OR “clean delivery”[tw] OR “delivery kits”[tw] OR “birth preparedness planning”[tw] OR “birth preparedness counseling”[tw] OR “birth preparedness education”[tw]OR "Patient Education as Topic"[Mesh] OR “health education”[tw] OR “health education”[mesh] OR “Delivery incentives”[tw] OR “Antenatal incentives”[tw] OR "Reimbursement, Incentive"[mesh] OR "Incentive reimbursements"[tw] OR "incentive reimbursement"[tw] OR "conditional cash transfer"[tw] OR "conditional cash transfers"[tw] OR "CCT"[tw] OR "health promotion/economics"[mesh] OR "health promotion/methods"[mesh] OR "health promotion/economics"[mesh] OR “vouchers”[tw] OR “voucher”[tw] OR “fee waiver”[tw] OR “fee waivers”[tw] OR “fee incentive”[tw] OR “Newborn resuscitation”[tw] OR “Thermal care”[tw] OR "Kangaroo-Mother Care Method"[Mesh] OR “Kangaroo mother care”[tw] OR “skin-to-skin”[tw] OR “breastfeeding”[tw] OR “umbilical cord care”[tw] OR “hygiene”[tw] OR “hand-washing practices”[tw] OR "Handwashing"[Mesh] OR “Hand Sanitization”[tw] OR “Hand Disinfection”[tw] OR “birth preparedness”[tw] OR “complication readiness”[tw] OR “complication readiness counseling”[tw] OR “complication readiness education”[tw] OR “post partum family planning”[tw] OR “post partum family planning counseling”[tw] OR “essential newborn care”[tw] OR “newborn care”[tw] OR "Hypothermia"[Mesh] OR “hypothermia”[tw] OR “Hypothermias”[tw] OR “Cigarette”[tw] OR “Cigarettes”[tw] OR "Smoking"[Mesh] **OR** "Tobacco Use Disorder"[Mesh] OR “Nicotine”[tw] OR "Substance-Related Disorders"[Mesh] OR “Drug Use”[tw] OR “Drug Usage”[tw] OR "Substance-Related Disorders"[Mesh] **OR** “Substance Abuses”[tw] OR “Substance Abuse”[tw] OR “Drug Dependence”[tw] OR "Street Drugs"[Mesh] OR "Tobacco"[Mesh] OR "Smoking"[Mesh] OR "Nicotine"[mesh] OR “alcohol”[tw] OR "Alcohol Drinking"[Mesh] OR “methamphetamine”[tw] OR “methamphetamines”[tw] OR "Methamphetamine"[Mesh] OR "Heroin Dependence"[Mesh] OR “heroin”[tw] OR "Heroin"[Mesh] OR "Cocaine-Related Disorders"[Mesh] OR "Cocaine"[Mesh] OR "Crack Cocaine"[Mesh] OR “cocaine”[tw] OR “opium”[tw] OR "Opium"[Mesh] OR “barbiturate”[tw] OR "Barbiturates"[Mesh] OR "Barbiturates"[tw] OR “amphetamines”[tw] **OR “Antenatal family planning counselling”[tw] OR "voluntary counselling and testing"[tw] OR “integrated counselling and testing”[tw] OR “birth preparedness counselling”[tw] OR “complication readiness counselling”[tw] OR “post partum family planning counselling”[tw] OR “prenatal family planning” [tw] OR “prenatal family planning counseling” [tw]**

**Concept 6: LMIC** (originally-used list)

“low-resource settings”[tw] OR “low-resource economies”[tw] OR "Lower-middle-income economies"[tw] OR “low income economies”[tw] OR "Developing countries"[mesh] OR "developing countries"[tw] OR "developing country"[tw] OR "under-developed countries"[tw] OR "under-developed country"[tw] OR "third-world countries"[tw] OR "third-world country"[tw] OR "developing nations"[tw] OR "developing nation"[tw] OR "under-developed nations"[tw] OR "third-world nations"[tw] OR "third-world nation"[tw] OR "less-developed countries"[tw] OR "less-developed country"[tw] OR "less-developed nations"[tw] OR “low and middle income countries”[tw] OR lmic[tw] OR “low income country”[tw] OR “low income countries”[tw] OR “lower income countries”[tw] OR “middle income country”[tw] OR “middle income countries”[tw] OR “lower middle income country”[tw] OR “lower middle income countries”[tw] OR “Least Developed Countries”[tw] OR Albania[tiab] OR Algeria[tiab] OR "American Samoa"[tiab] OR "Antigua and Barbuda"[tiab] OR Argentina[tiab] OR Azerbaijan[tiab] OR Belarus[tiab] OR "Bosnia and Herzegovina"[tiab] OR "Bosnia-Herzegovina"[tiab] OR Botswana[tiab] OR Brazil[tiab] OR Bulgaria[tiab] OR Chile[tiab] OR Colombia[tiab] OR "Costa Rica"[tiab] OR Cuba[tiab] OR Dominica[tiab] OR Dominican Republic[tiab] OR Fiji[tiab] OR Gabon[tiab] OR Grenada[tiab] OR Iran[tiab] OR Jamaica[tiab] OR Kazakhstan[tiab] OR Lebanon[tiab] OR Libya[tiab] OR Lithuania[tiab] OR Macedonia[tiab] OR Malaysia[tiab] OR Mauritius[tiab] OR Mayotte[tiab] OR Mexico[tiab] OR Montenegro[tiab] OR Namibia[tiab] OR Palau[tiab] OR Panama[tiab] OR Peru[tiab] OR Romania[tiab] OR Russia[tiab] OR "Russian Federation"[tiab] OR Serbia[tiab] OR Seychelles[tiab] OR "South Africa"[tiab] OR "Saint Kitts and Nevis"[tiab] OR "St Kitts and Nevis"[tiab] OR "Saint Vincent and the Grenadines"[tiab] OR "St Vincent and the Grenadines"[tiab] OR Suriname[tiab] OR Turkey[tiab] OR Uruguay[tiab] OR Venezuela[tiab] OR “Angola”[tiab] OR “Armenia”[tiab] OR “Belize”[tiab] OR “Bhutan”[tiab] OR “Bolivia”[tiab] OR “Cameroon”[tiab] OR "Cape Verde"[tiab] OR “China”[tiab] OR “Congo”[tiab] OR "Cote d'Ivoire"[tiab] OR "Ivory Coast"[tiab] OR” Djibouti”[tiab] OR “Ecuador”[tiab] OR “Egypt”[tiab] OR "El Salvador"[tiab] OR “Georgia”[tiab] OR “Guyana”[tiab] OR Guiana[tiab] OR Guatemala[tiab] OR Honduras[tiab] OR Indonesia[tiab] OR India[tiab] OR Iraq[tiab] OR Jordan[tiab] OR Kiribati[tiab] OR Kosovo[tiab] OR Lesotho[tiab] OR Maldives[tiab] OR “Johnston Island”[tiab] OR "Marshall Islands"[tiab] OR “Gilbert Islands”[tiab] OR “Nauru Marshall Islands”[tiab] OR “Pacific Islands”[tiab] OR “Tuvalu”[tiab] OR “Ellice Islands”[tiab] OR “Caroline Islands”[tiab] OR “Kiribati”[tiab] OR “Kosovo”[tiab] OR **“Lao PDR”[tiab] OR** “Marshall Islands”[tiab] OR **“Mauritania”[tiab]** OR “Basutoland”[tiab] OR Micronesia[tiab] OR “Moldavian SSR”[tiab] OR “Moldavian S.S.R”[tiab] OR Moldova[tiab] OR Mongolia[tiab] OR “Ifni”[tiab] OR Morocco[tiab] OR Nicaragua[tiab] OR Nigeria[tiab] OR Pakistan[tiab] OR "Papua New Guinea"[tiab] OR Paraguay[tiab] OR Philippines[tiab] OR Samoa[tiab] OR “Navigator Islands”[tiab] OR Senegal[tiab] OR "Sri Lanka"[tiab] OR Sudan[tiab] OR Swaziland[tiab] OR "Syrian Arab Republic"[tiab] OR Syria[tiab] OR Thailand[tiab] OR "Timor-Leste"[tiab] OR "East Timor"[tiab] OR Tonga[tiab] OR Tunisia[tiab] OR Turkmenistan[tiab] OR Tuvalu[tiab] OR Ukraine[tiab] OR Uzbekistan[tiab] OR Vanuatu[tiab] OR Vietnam[tiab] OR "West Bank and Gaza"[tiab] OR Yemen[tiab] OR **“Zambia”[tiab] OR** Afghanistan[tiab] OR Bangladesh[tiab] OR Benin[tiab] OR "Burkina Faso"[tiab] OR Burundi[tiab] OR Cambodia[tiab] OR "Central African Republic"[tiab] OR Chad[tiab] OR Comoros[tiab] OR "Democratic Republic of the Congo"[tiab] OR "Congo"[tiab] OR Eritrea[tiab] OR Ethiopia[tiab] OR Gambia[tiab] OR Ghana[tiab] OR Guinea[tiab] OR "Guinea-Bissau"[tiab] OR Haiti[tiab] OR Kenya[tiab] OR "Kyrgyz Republic"[tiab] OR "Kyrgyzstan"[tiab] OR "Lao PDR"[tiab] OR "Laos"[tiab] OR Liberia[tiab] OR Madagascar[tiab] OR Malawi[tiab] OR Mali[tiab] OR Mauritania[tiab] OR Myanmar[tiab] OR Nepal[tiab] OR Niger[tiab] OR Rwanda[tiab] OR "Sierra Leone"[tiab] OR "Solomon Islands"[tiab] OR Somalia[tiab] OR Tajikistan[tiab] OR Tanzania[tiab] OR Togo[tiab] OR Uganda[tiab] OR Zambia[tiab] OR Zimbabwe[tiab] OR Melanesia[tiab] OR “Zaire"[tiab] OR “Burma"[tiab] OR “Upper Volta"[tiab] OR ”Yugoslavia"[tiab]

**Scopus Syntax**

**Additional {OR{s added on 1/30/14 are marked in **red**. British-spelling versions of existing search terms are marked in **blue.** Moved iodine terms, now in correct concept 4, marked in **green** **

**ALL RESULTS SHOULD BE LIMITED TO THE PAST 30 YEARS**

Filters activated: Publication date from 1982/01/01 to 2012/07/31, English, human-only

**SETS**

Set 1: Screen and Treat

Antenatal terms (Concept 1)

AND

Conditions ({Blood Pressure{ et. al) (Concept 2)

AND

Screen Terminology (Concept 3)

RESULTS= 3,182

Set 2: Universal Dosing

Antenatal terms (Concept 1)

AND

Universal Dosing ({Calcium{ et. al) (Concept 4)

AND

LMIC List (Concept 6)

RESULTS= 6,095

Set 3: Behavioral/Counseling

Antenatal terms (Concept 1)

AND

Behavioral ({Antenatal family planning{ et. al) (Concept 5)

AND

LMIC List (Concept 6)

RECORDS= 10,855

**Concept 1: antenatal**

TITLE-ABS-KEY({Antenatal} OR {prenatal} OR {perinatal} OR {neonate} OR {neonates} OR {neonatal} OR {Newborn Infant} OR {Newborn Infants} OR {neonate} OR {neonates} OR {newborn} OR {newborns} OR {Infant} OR {Infant, Newborn} OR {infant} OR {infants} OR {neonate} OR {newborn} OR {baby} OR {babies} OR {Premature Birth} OR {Premature Birth} OR {Premature Births} OR {Preterm Births} **OR** {Preterm Birth} OR {Infant, Premature} OR {Premature Infant} OR {Premature Infants} OR {Premature Infant} OR {Prematurity} OR {Obstetric Labor, Premature} OR {Premature Labor} OR {Preterm Labor} OR {Premature Obstetric Labor} OR {Stillbirth} OR {Stillbirth} OR {Stillbirths} OR {fetal death} OR {Gestation} OR {Infant, Low Birth Weight} OR {Low Birth Weights} OR {Low Birth Weight} OR {Infant, Very Low Birth Weight} OR {Infant, Extremely Low Birth Weight} OR {Omphalitis} OR {postnatal} OR {post partum} OR {postpartum} OR {post partum} OR {Postnatal Care} OR {Postpartum Period} **OR {Premature Labour} OR {Preterm Labour} OR {Premature Obstetric Labour} OR {foetal death})**

**Concept 2: Interventions and Diseases**

({Blood pressure} OR {Blood Pressure} OR {Proteinuria} OR {proteinuria} OR {GU infection} OR {GenitoUrinary tract infection} OR {GenitoUrinary infection} OR {Urinary Tract Infections} OR {urinary tract infection} OR {urinary infection} OR {urinary infections} OR {Bacteriuria} OR {Pyuria} OR {Pyurias} OR {bacterial vaginosis} OR {Bacterial Vaginoses} OR {Bacterial Vaginitis} OR {genital tract infection} OR {genital tract infections} OR {reproductive tract infections} OR {Reproductive Tract Infection} OR {Genital Tract Infection} OR {Syphilis} OR {anemia} OR {Anemias} OR {Transplacental Exposure} OR {Maternal-Fetal Exchange} OR {Mother to child transmission} OR {MTCT} OR {prevention of mother-to-child transmission} OR {PMTCT} OR {prevention of parent-to-child transmission} OR {PPTCT} OR {HIV infected pregnant women} OR {maternal transmission} OR {transplacental transmission} **OR {Maternal-Foetal Exchange} OR {anaemia} OR {anaemias})**

**Concept 3: screening**

TITLE-ABS-KEY({screen*} OR {Neonatal Screening} OR {Mass Screening} OR {Prenatal Diagnosis} **OR {antenatal diagnosis})**

**Concept 4: Universal Dosing**

TITLE-ABS-KEY({Calcium} OR {Deworming} OR {IPTp} OR {intermittent preventive treatment} OR {iron supplements} OR {iron supplementation} OR {iron supplement} OR {iron folate supplements} OR {iron folate supplement} OR {Iron folate supplementation} OR {iron acid supplement} OR {iron acid supplements} OR {iron acid supplementation} OR {Diet Therapy} OR {therapeutic diets} OR {Nutritional Support} OR {Maternal Nutritional Physiological Phenomena} OR {Maternal diet} or {maternal nutritional supplementation} OR {maternal nutrition} OR {Micronutrient supplementation} OR {Tetanus toxoid} OR {Chlorhexidine} OR {misoprostol} OR {Retinol} OR {anthelmintic} OR {Antihelmintics} OR {Vermifuges} OR {albendazole} OR {Mebendazole} OR {Praziquantel} OR {Praziquantel} OR {beta Carotene} OR {Betacarotene} OR {vitamin A} OR {Tetanus Toxoid} OR {Tetanus Vaccine} OR {TT vaccine} OR {TT immunization} OR {Diphtheria-Tetanus-acellular Pertussis Vaccines} OR {Diphtheria-Tetanus-Pertussis Vaccine} OR {Diphtheria-Tetanus Vaccine} OR {Diphtheria Tetanus Vaccines} OR {DPT} OR {TDAP} **OR {Iodine} OR {Iodides} OR {iodized oils} OR {iodine-deficiency}**)

**Concept 5: Behavioral/Counseling**

TITLE-ABS-KEY({Antenatal family planning} OR {Antenatal family planning counseling} OR {voluntary counseling and testing} OR {VCTC} OR {integrated counseling and testing} OR {ICTC} OR {Birth-preparedness Programmes} OR {birth-preparedness package} OR {Birth-preparedness Programs} OR {clean delivery} OR {delivery kits} OR {birth preparedness planning} OR {birth preparedness counseling} OR {birth preparedness education}OR {Patient Education} OR {health education} OR {health education} OR {Delivery incentives} OR {Antenatal incentives} OR {Incentive reimbursements} OR {incentive reimbursement} OR {conditional cash transfer} OR {conditional cash transfers} OR {CCT} OR {health promotion } OR {vouchers} OR {voucher} OR {fee waiver} OR {fee waivers} OR {fee incentive} OR {Newborn resuscitation} OR {Thermal care} OR {Kangaroo-Mother Care Method} OR {Kangaroo mother care} OR {skin-to-skin} OR {breastfeeding} OR {umbilical cord care} OR {hygiene} OR {Handwashing} OR {Hand Sanitization} OR {Hand Disinfection} OR {birth preparedness} OR {complication readiness} OR {complication readiness counseling} OR {complication readiness education} OR {post partum family planning} OR {newborn care} OR {hypothermia} OR {Hypothermias} OR {Cigarette} OR {Cigarettes} OR {Smoking} **OR** {Tobacco Use Disorder} OR {Nicotine} OR {Substance-Related Disorders} OR {Drug Use} OR {Drug Usage} OR {Substance-Related Disorders} **OR** {Substance Abuses} OR {Substance Abuse} OR {Drug Dependence} OR {Street Drugs} OR {Tobacco} OR {Smoking} OR {alcohol} OR {methamphetamine} OR {methamphetamines} OR {heroin} OR {Cocaine} OR {Opium} OR {barbiturate} OR {Barbiturates} OR {amphetamines} **OR {Antenatal family planning counselling} OR {voluntary counselling and testing} OR {integrated counselling and testing} OR {birth preparedness counselling} OR {complication readiness counselling} OR {post partum family planning counselling} OR {prenatal family planning} OR {prenatal family planning counseling})**

**Concept 6: LMIC** (originally-used list)

TITLE-ABS-KEY({low-resource settings} OR {low-resource economies} OR {Lower-middle-income economies} OR {low income economies} OR {Developing countries} OR {developing countries} OR {developing country} OR {under-developed countries} OR {under-developed country} OR {third-world countries} OR {third-world country} OR {developing nations} OR {developing nation} OR {under-developed nations} OR {third-world nations} OR {third-world nation} OR {less-developed countries} OR {less-developed country} OR {less-developed nations} OR {low and middle income countries} OR {lmic} OR {low income country} OR {low income countries} OR {lower income countries} OR {middle income country} OR {middle income countries} OR {lower middle income country} OR {lower middle income countries} OR {Least Developed Countries} OR {Albania} OR {Algeria} OR {American Samoa} OR {Antigua and Barbuda} OR {Argentina} OR {Azerbaijan} OR {Belarus} OR {Bosnia and Herzegovina} OR {Bosnia-Herzegovina} OR {Botswana} OR {Brazil} OR {Bulgaria} OR {Chile} OR {Colombia} OR {Costa Rica} OR {Cuba} OR {Dominica} OR {Dominican Republic} OR {Fiji} OR {Gabon} OR {Grenada} OR {Iran} OR {Jamaica} OR {Kazakhstan} OR {Lebanon} OR {Libya} OR {Lithuania} OR {Macedonia} OR {Malaysia} OR {Mauritius} OR {Mayotte} OR {Mexico} OR {Montenegro} OR {Namibia} OR {Palau} OR {Panama} OR {Peru} OR {Romania} OR {Russia} OR {Russian Federation} OR {Serbia} OR {Seychelles} OR {South Africa} OR {Saint Kitts and Nevis} OR {St Kitts and Nevis} OR {Saint Vincent and the Grenadines} OR {St Vincent and the Grenadines} OR {Suriname} OR {Turkey} OR {Uruguay} OR {Venezuela} OR {Angola} OR {Armenia} OR {Belize} OR {Bhutan} OR {Bolivia} OR {Cameroon} OR {Cape Verde} OR {China} OR {Congo} OR {Cote d Ivoire} OR {Ivory Coast} OR{ Djibouti} OR {Ecuador} OR {Egypt} OR {El Salvador} OR {Georgia} OR {Guyana} OR {Guiana} OR {Guatemala} OR {Honduras} OR {Indonesia} OR {India} OR {Iraq} OR {Jordan} OR {Kiribati} OR {Kosovo} OR {Lesotho} OR {Maldives} OR {Johnston Island} OR {Marshall Islands} OR {Gilbert Islands} OR {Nauru Marshall Islands} OR {Pacific Islands} OR {Tuvalu} OR {Ellice Islands} OR {Caroline Islands} OR {Kiribati} OR {Kosovo} OR **{Lao PDR} OR** {Marshall Islands} OR **{Mauritania}** OR {Basutoland} OR {Micronesia} OR {Moldavian SSR} OR {Moldavian S.S.R} OR {Moldova} OR {Mongolia} OR {Ifni} OR {Morocco} OR {Nicaragua} OR {Nigeria} OR {Pakistan} OR {Papua New Guinea} OR {Paraguay} OR {Philippines} OR {Samoa} OR {Navigator Islands} OR {Senegal} OR {Sri Lanka} OR {Sudan} OR {Swaziland} OR {Syrian Arab Republic} OR {Syria} OR {Thailand} OR {Timor-Leste} OR {East Timor} OR {Tonga} OR {Tunisia} OR {Turkmenistan} OR {Tuvalu} OR {Ukraine} OR {Uzbekistan} OR {Vanuatu} OR {Vietnam} OR {West Bank and Gaza} OR {Yemen} OR **{Zambia} OR {**Afghanistan} OR {Bangladesh} OR {Benin} OR {Burkina Faso} OR {Burundi} OR {Cambodia} OR {Central African Republic} OR {Chad} OR {Comoros} OR {Democratic Republic of the Congo} OR {Congo} OR {Eritrea} OR {Ethiopia} OR {Gambia} OR {Ghana} OR {Guinea} OR {Guinea-Bissau} OR {Haiti} OR {Kenya} OR {Kyrgyz Republic} OR {Kyrgyzstan} OR {Lao PDR} OR {Laos} OR {Liberia} OR {Madagascar} OR {Malawi} OR {Mali} OR {Mauritania} OR {Myanmar} OR {Nepal} OR {Niger} OR {Rwanda} OR {Sierra Leone} OR {Solomon Islands} OR {Somalia} OR {Tajikistan} OR {Tanzania} OR {Togo} OR {Uganda} OR {Zambia} OR {Zimbabwe} OR {Melanesia} OR {Zaire} OR {Burma} OR {Upper Volta} OR {Yugoslavia})

**Embase Syntax**

**Additional “OR”s added on 1/30/14 are marked in **red**. British-spelling versions of existing search terms are marked in **blue.** Moved iodine terms, now in correct concept 4, marked in **green** **

**ALL RESULTS SHOULD BE LIMITED TO THE PAST 30 YEARS**

Filters activated: Publication date from 1982/01/01 to 2012/07/31, English, human-only

**SETS**

Set 1: Screen and Treat

Antenatal terms (Concept 1)

AND

Conditions (“Blood Pressure” et. al) (Concept 2)

AND

Screen Terminology (Concept 3)

RECORDS= 13,073

Set 2: Universal Dosing

Antenatal terms (Concept 1)

AND

Universal Dosing (“Calcium” et. al) (Concept 4)

AND

LMIC List (Concept 6)

RECORDS= 4,239

Set 3: Behavioral/Counseling

Antenatal terms (Concept 1)

AND

Behavioral (“Antenatal family planning” et. al) (Concept 5)

AND

LMIC List (Concept 6)

RECORDS= 9,160

**Concept 1: antenatal**

“prenatal period”/exp OR “newborn”/exp OR “Infant”/exp OR "Prematurity"/exp OR 'premature labor'/exp OR “Low Birth Weight"/de OR "Very Low Birth Weight"/de OR "Extremely Low Birth Weight"/de OR 'puerperium'/de OR "Stillbirth"/de OR 'immature and premature labor'/exp OR “Antenatal” OR “prenatal” OR “perinatal” OR “neonate” OR “neonates” OR “neonatal” OR “Newborn Infant” OR “Newborn Infants” OR “neonate” OR “neonates” OR “newborn” OR “newborns” OR "Infant" OR “Infant, Newborn” OR “infant” OR “infants” OR “neonate” OR “newborn” OR “baby” OR “babies” OR "Premature Birth" OR “Premature Birth” OR “Premature Births” OR “Preterm Births” **OR** “Preterm Birth” OR "Infant, Premature" OR “Premature Infant” OR “Premature Infants” OR “Premature Infant” OR “Prematurity” OR "Obstetric Labor, Premature" OR “Premature Labor” OR “Preterm Labor” OR “Premature Obstetric Labor” OR "Stillbirth" OR "Stillbirth" OR "Stillbirths" OR “fetal death” OR “Gestation” OR "Infant, Low Birth Weight" OR “Low Birth Weights” OR “Low Birth Weight” OR "Infant, Very Low Birth Weight" OR "Infant, Extremely Low Birth Weight" OR “Omphalitis” OR “postnatal” OR “post partum” OR “postpartum” OR “post partum” OR "Postnatal Care" OR "Postpartum Period" **OR "Premature Labour" OR "Preterm Labour" OR "Premature Obstetric Labour" OR “foetal death”**

**Concept 2: Interventions and Diseases**

"Blood Pressure"/exp OR "Blood Pressure" OR “proteinuria"/exp OR “proteinuria” OR “GU infection” OR “GenitoUrinary tract infection” OR “GenitoUrinary infection” OR “GenitoUrinary infection” OR "Urinary Tract Infections" OR "Urinary Tract Infections" OR “Urinary tract infection"/exp OR “urinary infection” OR “urinary infections” OR "Bacteriuria" OR "Bacteriuria"/de OR “Pyuria" OR “Pyuria" OR “Pyurias” OR "Vaginosis, Bacterial" OR “bacterial vaginosis” OR “Bacterial Vaginoses” OR “Bacterial Vaginitis” OR “Vaginitis”/de OR “genital tract infection” OR “genital tract infections” OR "reproductive tract infections" OR "reproductive tract infections" OR “Reproductive Tract Infection” OR “Genital Tract Infections” OR 'genital tract infection'/de OR “Genital Tract Infection” OR “Syphilis"/de OR "Syphilis" OR “Syphilis” OR “anemia"/de OR “anemia” OR Anemias OR 'iron deficiency anemia'/de OR “Transplacental Exposure" OR "Maternal-Fetal Exchange" OR "Maternal-Fetal Exchange" OR 'fetomaternal transfusion'/de OR “Mother to child transmission” OR “MTCT” OR “prevention of mother-to-child transmission” OR “PMTCT” OR “prevention of parent-to-child transmission” OR “PPTCT” OR “HIV infected pregnant women” OR “maternal transmission” OR “transplacental transmission” **OR "Maternal-Foetal Exchange" OR “anaemia” OR “anaemia”/de OR “anaemias”**

**Concept 3: screening**

screen* OR "Neonatal Screening" OR 'newborn screening'/exp OR 'mass screening'/exp OR "Prenatal Diagnosis"/exp OR "Mass Screening" OR "Prenatal Diagnosis" **OR “antenatal diagnosis”**

**Concept 4: Universal Dosing**

"Calcium” OR "Calcium”/exp OR “Deworming” OR “IPTp” OR “intermittent preventive treatment” OR "Dietary iron" OR **"Iron intake”/exp** OR “iron supplements” OR “iron supplementation” OR “iron supplement” OR “iron folate supplements” OR “iron folate supplement” OR “Iron folate supplementation” OR “iron acid supplement” OR “iron acid supplements” OR “iron acid supplementation” OR "Diet Therapy" OR "Diet Therapy”/exp OR “therapeutic diets” OR "Nutritional Support”/exp OR "Nutritional Support" OR "Maternal Nutritional Physiological Phenomena" OR “Maternal diet” or “maternal nutritional supplementation” OR “maternal nutrition” OR “maternal nutrition”/exp OR “Micronutrient supplementation” OR "Tetanus Toxoid" OR “Tetanus toxoid” OR “Tetanus toxoid”/exp OR “Chlorhexidine” OR "Chlorhexidine" OR "Chlorhexidine”/exp OR "Misoprostol" OR “misoprostol” OR “Retinol” OR “anthelmintic” OR “Antihelmintics” OR “Vermifuges” OR "Anthelmintics" OR "Albendazole" OR “albendazole” OR "Mebendazole" OR “mebendazole” OR "Praziquantel" OR "Praziquantel" OR "beta Carotene" OR “beta carotene” OR “Betacarotene” OR "Vitamin A" OR “vitamin A” OR “vitamin A supplementation” OR “vitamin A supplements” OR “vitamin A supplement” OR “vitamin A dosing” OR “vitamin A dose” OR "Tetanus Toxoid" OR “Tetanus Vaccine” OR “TT vaccine” OR “TT immunization” OR "Diphtheria-Tetanus-acellular Pertussis Vaccines" OR “Diphtheria Tetanus acellular Pertussis Vaccines” OR "Diphtheria-Tetanus-Pertussis Vaccine" OR "Diphtheria-Tetanus Vaccine" OR “Diphtheria Tetanus Vaccine” OR “DPT” OR “TDAP” OR “misoprostol”/exp OR “Retinol”/exp OR 'anthelmintic agent'/exp OR “mebendazole”/exp **OR "Praziquantel”/exp**  OR “beta carotene”/exp OR "Diphtheria Tetanus acellular Pertussis Vaccine”/exp **OR 'iodine'/de OR 'iodide'/de OR 'iodine deficiency'/de OR "Iodine" OR "Iodides" OR "iodized oils" OR "iodides" OR "iodine-deficiency"**

**Concept 5: Behavioral/Counseling**

"Opiate”/exp OR "Cocaine”/exp OR 'diamorphine'/exp OR “smoking”/exp OR “Substance Abuse”/exp OR 'nicotine'/exp OR “hypothermia"/exp OR “newborn care"/exp OR “hygiene"/exp OR 'breast feeding education'/exp OR “Kangaroo care"/exp OR "health promotion"/exp OR "Reimbursement"/exp OR “Antenatal family planning” OR “voluntary counseling and testing” OR “VCTC” OR “integrated counseling and testing” OR “ICTC” OR “Birth-preparedness Programmes” OR “birth-preparedness package” OR “Birth-preparedness Programs” OR “clean delivery” OR “delivery kits” OR “birth preparedness planning” OR “birth preparedness counseling” OR “birth preparedness education” OR “health education” OR “Delivery incentives” OR “Antenatal incentives” OR "Incentive reimbursements" OR "incentive reimbursement" OR "conditional cash transfer" OR "conditional cash transfers" OR "CCT" OR “vouchers” OR “voucher” OR “fee waiver” OR “fee waivers” OR “fee incentive” OR “Newborn resuscitation” OR “Thermal care” OR “Kangaroo mother care” OR “skin-to-skin” OR “breastfeeding” OR “umbilical cord care” OR “hygiene” OR “hygiene” OR “hand-washing practices” OR "Handwashing" OR “Hand Sanitization” OR “Hand Disinfection” OR “birth preparedness” OR “complication readiness” OR “complication readiness education” OR “post partum family planning” OR “post partum family planning counseling” OR “essential newborn care” OR “newborn care” OR "Hypothermia" OR “hypothermia” OR “Hypothermias” OR “Cigarette” OR “Cigarettes” OR "Smoking" **OR** "Tobacco Use Disorder" OR “Nicotine” OR "Substance-Related Disorders" OR “Drug Use” OR “Drug Usage” OR "Substance-Related Disorders" **OR** “Substance Abuses” OR “Substance Abuse” OR “Drug Dependence” OR "Street Drugs" OR "Tobacco" OR "Smoking" OR "Nicotine" OR “alcohol” OR "Alcohol Drinking" OR “methamphetamine” OR “methamphetamines” OR "Methamphetamine" OR "Heroin Dependence" OR “heroin” OR "Heroin" OR "Cocaine-Related Disorders" OR "Cocaine" OR "Crack Cocaine" OR “cocaine” OR “opium” OR "Opium" OR “barbiturate” OR "Barbiturates" OR "Barbiturates" OR “amphetamines” **OR “Antenatal family planning counselling” OR "voluntary counselling and testing" OR “integrated counselling and testing” OR “birth preparedness counselling” OR “complication readiness counselling” OR “post partum family planning counselling” OR “prenatal family planning” OR “prenatal family planning counseling”**

**Concept 6: LMIC** (originally-used list)

(“low-resource settings” OR “low-resource economies” OR "Lower-middle-income economies" OR “low income economies” OR "Developing countries" OR "developing countries" OR "developing country" OR "under-developed countries" OR "under-developed country" OR "third-world countries" OR "third-world country" OR "developing nations" OR "developing nation" OR "under-developed nations" OR "third-world nations" OR "third-world nation" OR "less-developed countries" OR "less-developed country" OR "less-developed nations" OR “low and middle income countries” OR lmic OR “low income country” OR “low income countries” OR “lower income countries” OR “middle income country” OR “middle income countries” OR “lower middle income country” OR “lower middle income countries” OR “Least Developed Countries” OR Albania OR Algeria OR "American Samoa" OR "Antigua and Barbuda" OR Argentina OR Azerbaijan OR Belarus OR "Bosnia and Herzegovina" OR "Bosnia-Herzegovina" OR Botswana OR Brazil OR Bulgaria OR Chile OR Colombia OR "Costa Rica" OR Cuba OR Dominica OR Dominican Republic OR Fiji OR Gabon OR Grenada OR Iran OR Jamaica OR Kazakhstan OR Lebanon OR Libya OR Lithuania OR Macedonia OR Malaysia OR Mauritius OR Mayotte OR Mexico OR Montenegro OR Namibia OR Palau OR Panama OR Peru OR Romania OR Russia OR "Russian Federation" OR Serbia OR Seychelles OR "South Africa" OR "Saint Kitts and Nevis" OR "St Kitts and Nevis" OR "Saint Vincent and the Grenadines" OR "St Vincent and the Grenadines" OR Suriname OR Turkey OR Uruguay OR Venezuela OR “Angola” OR “Armenia” OR “Belize” OR “Bhutan” OR “Bolivia” OR “Cameroon” OR "Cape Verde" OR “China” OR “Congo” OR "Cote d'Ivoire" OR "Ivory Coast" OR "Djibouti” OR “Ecuador” OR “Egypt” OR "El Salvador" OR “Georgia” OR “Guyana” OR Guiana OR Guatemala OR Honduras OR Indonesia OR India OR Iraq OR Jordan OR Kiribati OR Kosovo OR Lesotho OR Maldives OR “Johnston Island” OR "Marshall Islands" OR “Gilbert Islands” OR “Nauru Marshall Islands” OR “Pacific Islands” OR “Tuvalu” OR “Ellice Islands” OR “Caroline Islands” OR “Kiribati” OR “Kosovo” OR **“Lao PDR” OR** “Marshall Islands” OR **“Mauritania”** OR “Basutoland” OR Micronesia OR “Moldavian SSR” OR “Moldavian S.S.R” OR Moldova OR Mongolia OR “Ifni” OR Morocco OR Nicaragua OR Nigeria OR Pakistan OR "Papua New Guinea" OR Paraguay OR Philippines OR Samoa OR “Navigator Islands” OR Senegal OR "Sri Lanka" OR Sudan OR Swaziland OR "Syrian Arab Republic" OR Syria OR Thailand OR "Timor-Leste" OR "East Timor" OR Tonga OR Tunisia OR Turkmenistan OR Tuvalu OR Ukraine OR Uzbekistan OR Vanuatu OR Vietnam OR "West Bank and Gaza" OR Yemen OR **“Zambia” OR** Afghanistan OR Bangladesh OR Benin OR "Burkina Faso" OR Burundi OR Cambodia OR "Central African Republic" OR Chad OR Comoros OR "Democratic Republic of the Congo" OR "Congo" OR Eritrea OR Ethiopia OR Gambia OR Ghana OR Guinea OR "Guinea-Bissau" OR Haiti OR Kenya OR "Kyrgyz Republic" OR "Kyrgyzstan" OR "Lao PDR" OR "Laos" OR Liberia OR Madagascar OR Malawi OR Mali OR Mauritania OR Myanmar OR Nepal OR Niger OR Rwanda OR "Sierra Leone" OR "Solomon Islands" OR Somalia OR Tajikistan OR Tanzania OR Togo OR Uganda OR Zambia OR Zimbabwe OR Melanesia OR “Zaire" OR “Burma" OR “Upper Volta" OR ”Yugoslavia"):ab,ti

**Global Health Syntax**

**Additional ORs added on 1/30/14 are marked in **red**. British-spelling versions of existing search terms are marked in **blue.** Moved iodine terms, now in correct concept 4, marked in **green****

**ALL RESULTS SHOULD BE LIMITED TO THE PAST 30 YEARS**

Filters activated: Publication date from 1982/01/01 to 2012/07/31, English, human-only

**SETS**

Set 1: Screen and Treat

Antenatal terms (Concept 1)

AND

Conditions (Blood Pressure et. al) (Concept 2)

AND

Screen Terminology (Concept 3)

RESULTS= 1327

Set 2: Universal Dosing

Antenatal terms (Concept 1)

AND

Universal Dosing (Calcium et. al) (Concept 4)

AND

LMIC List (Concept 6)

RESULTS =1044

Set 3: Behavioral/Counseling

Antenatal terms (Concept 1)

AND

Behavioral (Antenatal family planning et. al) (Concept 5)

AND

LMIC List (Concept 6)

**Concept 1: antenatal**

Exp neonates/ OR exp infants/ OR exp **low birth weight infants/ OR exp fetal death/ OR** exp **premature infants/ OR** (Antenatal OR prenatal OR perinatal OR neonate OR neonates OR neonatal OR newborn OR newborns OR Infant OR infants OR baby OR babies OR Premature Birth OR Premature Births OR Preterm Births OR Preterm Birth OR Premature Infants OR Premature Infant OR Prematurity OR Premature Labor OR Preterm Labor OR Premature Obstetric Labor OR Stillbirth OR Stillbirths OR Low Birth Weights OR Low Birth Weight OR postnatal OR post partum OR postpartum OR fetal death OR Gestation OR Low Birth Weights OR Low Birth Weight OR Omphalitis OR **Premature Labour OR Preterm Labour OR Premature Obstetric Labour OR foetal death**).af.

**Concept 2: Interventions and Diseases**

Exp Blood pressure/ OR exp Proteinuria/ OR exp Urinary Tract Infections/ OR exp Bacteriuria/ OR exp Bacterial Vaginitis/ OR exp Syphilis/ OR exp transplacental transmission/ OR exp Maternal-Fetal Exchange/ OR exp iron deficiency anaemia/ OR (Blood Pressure OR proteinuria OR GU infection OR GenitoUrinary tract infection OR GenitoUrinary infection OR GenitoUrinary infections OR Urinary Tract Infections OR urinary tract infection OR urinary infection OR urinary infections OR Bacteriuria OR Pyuria OR Pyurias OR bacterial vaginosis OR Bacterial Vaginoses OR Bacterial Vaginitis OR genital tract infection OR genital tract infections OR reproductive tract infections OR Reproductive Tract Infection OR Genital Tract Infections OR Genital Tract Infection OR Syphilis OR anemia OR Anemias OR Transplacental Exposure OR **transplacental transmission** OR Maternal-Fetal Exchange OR Mother to child transmission OR parent-to-child transmission OR PPTCT OR HIV infected pregnant women OR maternal transmission OR transplacental transmission **OR Maternal-Foetal Exchange OR anaemia OR anaemias**).af.

**Concept 3: screening**

exp **screening/ OR** exp **prenatal diagnosis/** OR (screen OR **screening** OR Neonatal Screening OR Mass Screening OR Prenatal Diagnosis OR **antenatal diagnosis**).af.

**Concept 4: Universal Dosing**

Exp Calcium/ OR exp Nutritional Support/OR exp therapeutic diets/ OR exp maternal nutrition/ OR exp Tetanus Toxoid/ OR exp Chlorhexidine/ OR exp Misoprostol/ OR exp Retinol/ OR exp anthelmintics/ OR exp Albendazole/ OR exp Praziquantel/ OR (beta Carotene OR Calcium supplementation OR Deworming OR IPTp OR intermittent preventive treatment OR Dietary iron OR iron supplements OR iron supplementation OR iron supplement OR iron folate supplements OR iron folate supplement OR Iron folate supplementation OR iron acid supplement OR iron acid supplements OR iron acid supplementation OR Diet Therapy OR therapeutic diets OR Nutritional Support OR Maternal Nutritional Physiological Phenomena OR Maternal diet or maternal nutritional supplementation OR maternal nutrition OR Micronutrient supplementation OR Tetanus toxoid OR Chlorhexidine OR misoprostol OR Retinol OR anthelmintic OR Antihelmintics OR Vermifuges OR Albendazole OR mebendazole OR Praziquantel OR beta carotene OR Betacarotene OR vitamin A OR Tetanus Vaccine OR TT vaccine OR TT immunization OR Diphtheria-Tetanus-acellular Pertussis Vaccines OR Diphtheria-Tetanus-Pertussis Vaccine OR Diphtheria-Tetanus Vaccines OR Diphtheria Tetanus Vaccine OR DPT OR TDAP OR Iodine OR Iodides OR iodized oils OR iodides).af

**Concept 5: Behavioral/Counseling**

Exp health education/ OR exp Patient Education/ OR exp health promotion/ OR exp hygiene/ OR exp hypothermia/ OR exp Cigarettes/ OR exp Smoking/ OR exp Tobacco/ OR exp Nicotine/ OR exp Substance Abuse/ OR exp Drug abuse/ OR exp Drinking/ OR exp heroin/ OR exp Cocaine/ OR exp Opium/ OR exp Barbiturates/ OR (Antenatal family planning OR voluntary counseling and testing OR VCTC OR integrated counseling and testing OR ICTC OR clean delivery OR delivery kits OR Patient Education OR health education OR Delivery incentives OR Antenatal incentives OR Incentive reimbursements OR incentive reimbursement OR conditional cash transfer OR conditional cash transfers OR CCT OR health promotion OR vouchers OR voucher OR fee waiver OR fee waivers OR fee incentive OR Newborn resuscitation OR Thermal care OR Kangaroo mother care OR skin-to-skin OR breastfeeding OR umbilical cord care OR hygiene OR Handwashing OR Hand Sanitization OR Hand Disinfection OR birth preparedness OR complication readiness OR post partum family planning OR Hypothermia OR Hypothermias OR Cigarette OR Cigarettes OR Smoking OR Nicotine OR Substance-Related Disorders OR Drug Use OR Drug Usage OR Substance-Related Disorders **OR** Substance Abuses OR Substance Abuse OR Drug Dependence OR Street Drugs OR Tobacco OR alcohol OR methamphetamine OR heroin OR cocaine OR Opium OR barbiturate OR Barbiturates OR amphetamines **OR Antenatal family planning counselling OR voluntary counselling and testing OR integrated counselling and testing OR birth preparedness counselling OR complication readiness counselling OR post partum family planning counselling OR prenatal family planning**).af.

**Concept 6: LMIC** (originally-used list)

(low-resource settings OR low-resource economies OR Lower-middle-income economies OR low income economies OR Developing countries OR developing countries OR developing country OR under-developed countries OR under-developed country OR third-world countries OR third-world country OR developing nations OR developing nation OR under-developed nations OR third-world nations OR third-world nation OR less-developed countries OR less-developed country OR less-developed nations OR **Least Developed Countries** OR low and middle income countries OR lmic OR low income country OR low income countries OR lower income countries OR middle income country OR middle income countries OR lower middle income country OR lower middle income countries OR Afghanistan).ti,ab.

(Albania OR Algeria OR American Samoa OR Antigua and Barbuda OR Argentina OR Azerbaijan OR Belarus OR Bosnia and Herzegovina OR Bosnia-Herzegovina OR Botswana OR Brazil OR Bulgaria OR Chile OR Colombia OR Costa Rica OR Cuba OR Dominica OR Dominican Republic OR Fiji OR Gabon OR Grenada OR Iran OR Jamaica OR Kazakhstan OR Lebanon OR Libya OR Lithuania OR Macedonia OR Malaysia OR Mauritius OR Mayotte OR Mexico OR Montenegro OR Namibia OR Palau OR Panama OR Peru OR Romania OR Russia OR Russian Federation OR Serbia OR Seychelles OR South Africa OR Saint Kitts and Nevis OR St Kitts and Nevis OR Saint Vincent and the Grenadines OR St Vincent and the Grenadines OR Suriname OR Turkey OR Uruguay OR Venezuela).ti,ab.

(Angola OR Armeni OR Belize OR Bhutan OR Bolivia OR Cameroon OR Cape Verde OR China OR Congo OR Cote d'Ivoire OR Ivory Coast OR Djibouti OR Ecuador OR Egypt OR El Salvador OR Georgia OR Guyana OR Guiana OR Guatemala OR Honduras OR Indonesia OR India OR Iraq OR Jordan OR Kiribati OR Kosovo OR Lesotho OR Maldives OR Johnston Island OR Marshall Islands OR Gilbert Islands OR Mariana Islands OR Nauru Marshall Islands OR Pacific Islands OR Tuvalu OR Ellice Islands OR Caroline Islands OR **Lao PDR OR** Marshall Islands OR **Mauritania** OR Basutoland OR Micronesia OR Moldavian SSR OR Moldavian SSR OR Moldova OR Mongolia OR Ifni OR Morocco OR Nicaragua OR Nigeria OR Pakistan OR Papua New Guinea OR Paraguay OR Philippines OR Samoa OR Sao Tome and Principe OR Navigator Islands OR Senegal OR Sri Lanka OR Sudan OR Swaziland OR Syrian Arab Republic OR Syria OR Thailand OR Timor-Leste OR East Timor OR Tonga OR Tunisia OR Turkmenistan OR Tuvalu OR Ukraine OR Uzbekistan OR Vanuatu OR Vietnam OR West Bank and Gaza OR Yemen OR **Zambia OR** Afghanistan OR Bangladesh OR Benin OR Burkina Faso OR Burundi OR Cambodia OR Central African Republic OR Chad OR Comoros OR Democratic Republic of the Congo OR Congo OR Eritrea OR Ethiopia OR Gambia OR Ghana OR Guinea OR Guinea-Bissau OR Haiti OR Kenya OR Korea OR Kyrgyz Republic OR Kyrgyzstan OR Laos OR Liberia OR Madagascar OR Malawi OR Mali OR Mauritania OR Myanmar OR Nepal OR Niger OR Rwanda OR Sierra Leone OR Solomon Islands OR Somalia OR Tajikistan OR Tanzania OR Togo OR Uganda OR Zambia OR Zimbabwe OR Melanesia OR **Zaire**  **OR Burma**  **OR Upper Volta**  **OR Yugoslavia).ti,ab.**

**Popline**

From Emails 3.18

**Concept 1**

Antenatal  OR prenatal OR neonate  OR neonates  OR neonatal  OR newborn  OR newborns  OR Infant  OR infants   OR baby  OR babies  OR "Premature Birth"  OR "Premature Births"   OR "Preterm Births" OR "Preterm Birth"   OR Prematurity   OR "Premature Labor"   OR "Preterm Labor"   OR "Premature Obstetric Labor"   OR Stillbirth  OR  Stillbirths OR "fetal death" OR "Low Birth Weights"  OR "Low Birth Weight"  OR postnatal OR "post partum"   OR postpartum OR “perinatal” OR “Gestation” OR “Omphalitis” OR “postnatal  OR “post partum” OR postpartum **OR  "Premature Labour" OR "Preterm Labour" OR "Premature Obstetric Labour" OR “foetal death”**

**Updated to:**

(Antenatal  OR prenatal OR neonate  OR neonates  OR neonatal  OR newborn  OR newborns  OR Infant  OR infants   OR baby  OR babies  OR "Preterm Births" OR "Preterm Birth"   OR Prematurity   OR "Premature"   OR "Preterm Labor"   OR Stillbirth  OR  Stillbirths OR "fetal death" OR "Low Birth Weights"  OR "Low Birth Weight" OR "post partum"   OR postpartum OR perinatal OR Gestation OR Omphalitis OR postnatal  OR "post partum" OR  "Preterm Labour" OR "foetal death")

**Concept 2**

"Blood pressure" OR proteinuria   OR "GU infection"  OR "GenitoUrinary infection"  OR "GenitoUrinary infection" OR “GenitoUrinary tract infection” OR “GenitoUrinary tract infections”  OR "Urinary Tract Infections" OR "urinary tract infection" OR "urinary tract infections" OR "urinary infection"  OR "urinary infections"   OR Bacteriuria  OR Pyuria  OR Pyurias   OR "Bacterial Vaginosis" OR "Bacterial Vaginoses"  OR "Bacterial Vaginitis"  OR "genital tract infection"  OR "genital tract infections"   OR "reproductive tract infection" OR "reproductive tract infections"  OR Syphilis   OR anemia  OR Anemias OR **"**Transplacental Exposure"  OR "transplacental transmission" OR "maternal transmission" OR "Maternal-Fetal Exchange"  OR "Mother to child transmission"  OR MTCT  OR PMTCT  OR "prevention of parent-to-child transmission"  OR PPTCT  OR "HIV infected pregnant women" OR “maternal transmission” OR “transplacental transmission” **OR "Maternal-Foetal Exchange" OR “anaemia”  OR “anaemias”**

**Concept 3**

Screen OR  Screening OR "Prenatal Diagnosis" **OR “antenatal diagnosis”**

**Concept 4**

**Concept 5**

**("Antenatal family planning"  OR  "voluntary counseling and testing"  OR  VCTC  OR  "integrated counseling and testing" OR  ICTC  OR "clean delivery"  OR   "delivery kits"  OR  "birth preparedness"  OR  "Patient Education"  OR  "health education"   OR  "Delivery incentives" OR  "Antenatal incentives"  OR  "Incentive reimbursements"  OR  "incentive reimbursement"  OR  "conditional cash transfer"  OR  "conditional cash transfers"  OR  CCT  OR  "health promotion" OR  vouchers  OR  voucher  OR  "fee waiver"  OR  "fee waivers"  OR  "fee incentive"  OR  "Newborn resuscitation"  OR  "Thermal care"  OR  kangaroo  OR  breastfeeding   OR  hygiene  OR  Handwashing  OR  "Hand Disinfection"  OR  "post partum family" OR  "newborn care"   OR  Hypothermia   OR  Smoking OR Tobacco OR Drugs)**

**Concept 6**

("low-resource settings" OR  "low-resource economies" OR  "Lower-middle-income economies"  OR  "low income economies" OR  "Developing countries"  OR  "developing countries"  OR  "developing country"  OR  "under-developed countries" OR "under-developed country" OR "developing nations" OR  "developing nation" OR "under-developed nations" OR "less-developed countries" OR "Least Developed Countries"  OR  lmic  OR  "low income country" OR "low income countries"  OR  "lower income countries"  OR  "middle income country" OR  "middle income countries")
