# Supplementary material for: A New Look at Care in Pregnancy: Simple, Effective Interventions for Neglected Populations
Source: PLoS One. 2016 Aug 18;11(8):e0160562. doi: 10.1371/journal.pone.0160562 (PMC4990268; doi:10.1371/journal.pone.0160562)

**S1: Additional Methodologic Notes**

For the initial literature search we identified studies by querying the following electronic databases: PubMed, Embase, Scopus, Global Health (Ovid), LILACS, and Popline. The search was limited to articles published since 1982 in the English language. A systematic literature search was applied to PubMed’s Medline from 1946 to the present. The search was adapted for Embase (1974-present), Scopus (1966-present), Global Health (1910-present), LILACS (1966-present), and Popline (1970-present). To identify publications related to antenatal interventions and screening in low and middle income countries, we used a combination of controlled vocabulary and text words including combinations of: “antenatal,” “prenatal,” "neonatal,” screen*, “neonatal screening," "mass screening," "prenatal diagnosis" (see annex for complete search).

A comprehensive set of search terms for all interventions was created in collaboration with an informationist at the Welch Medical Library at Johns Hopkins University (see annex) based on inclusion/exclusion criteria as described above. We sought to capture experimental and observational studies that connected a chosen intervention to the mortality and intermediate endpoints of interest which were published between 1982 and October 2014. Some more recent publications were also included if they addressed a particularly data-sparse question. Systematic reviews related to interventions of interest and published in 2000 or later were also included. Search results were checked to determine the inclusion of a known set of included citations, and the results were reviewed by the research team and the strategy developed iteratively. We excluded cost effectiveness studies, recommendation papers and studies focused on acceptability, use or knowledge.

Results were collected in Endnote and narrowed within Excel by graduate students between late 2012 and mid-2013. Syntax errors, which had altered the scope and quantity of articles, were discovered in the search terminology. An updated set of search terms was created, correcting the syntax errors, and was run through the same databases in early 2014. The results were aggregated in Endnote and crossed with the 2012/2013 results to remove duplicates. The remaining results were filtered by additional keywords in Endnote and Excel by a graduate student.

Titles and abstracts of remaining articles were read in Excel; full texts of finalists were read and final lists for each intervention, including articles captured in the original search, were created. Final decisions on the inclusion of articles were made by the principal investigators and based on additional inclusion/exclusion criteria specific to each intervention.

**Search Inclusion and Exclusion Criteria**

| **Inclusion** | **Exclusion** |
| --- | --- |
| • Includes a form of intervention, unless otherwise noted  • Outcomes of interest: maternal or neonatal mortality, preterm birth, low birth weight, length of gestation and any  other directly-connected secondary outcome (depending on  issue)  • Interventions would be feasible in a developing country context  • Studies focused on sensitivity and specificity of screening tools currently recommended, so long as they meet other  inclusion criteria  • Studies looking at treatment without screening so long as treatment is currently recommended  • Systematic reviews conducted during or after 2000  • Low & middle income countries only  • Date range of publication: 01/01/1982 to 10/31/2014  • English; Human-only | • Studies without interventions (i.e. epidemiologic studies), unless otherwise noted  • Studies without interventions (i.e. epidemiologic studies), unless otherwise noted  • Cost-effectiveness studies and those focused on acceptability, use and knowledge (and not links to  mortality)  • Papers with only recommendations  • Studies focused on outdated screening or treatment protocols that are no longer recommended  • Systematic reviews published before 2000  • Studies in high-income countries  • Studies out of chosen date range |


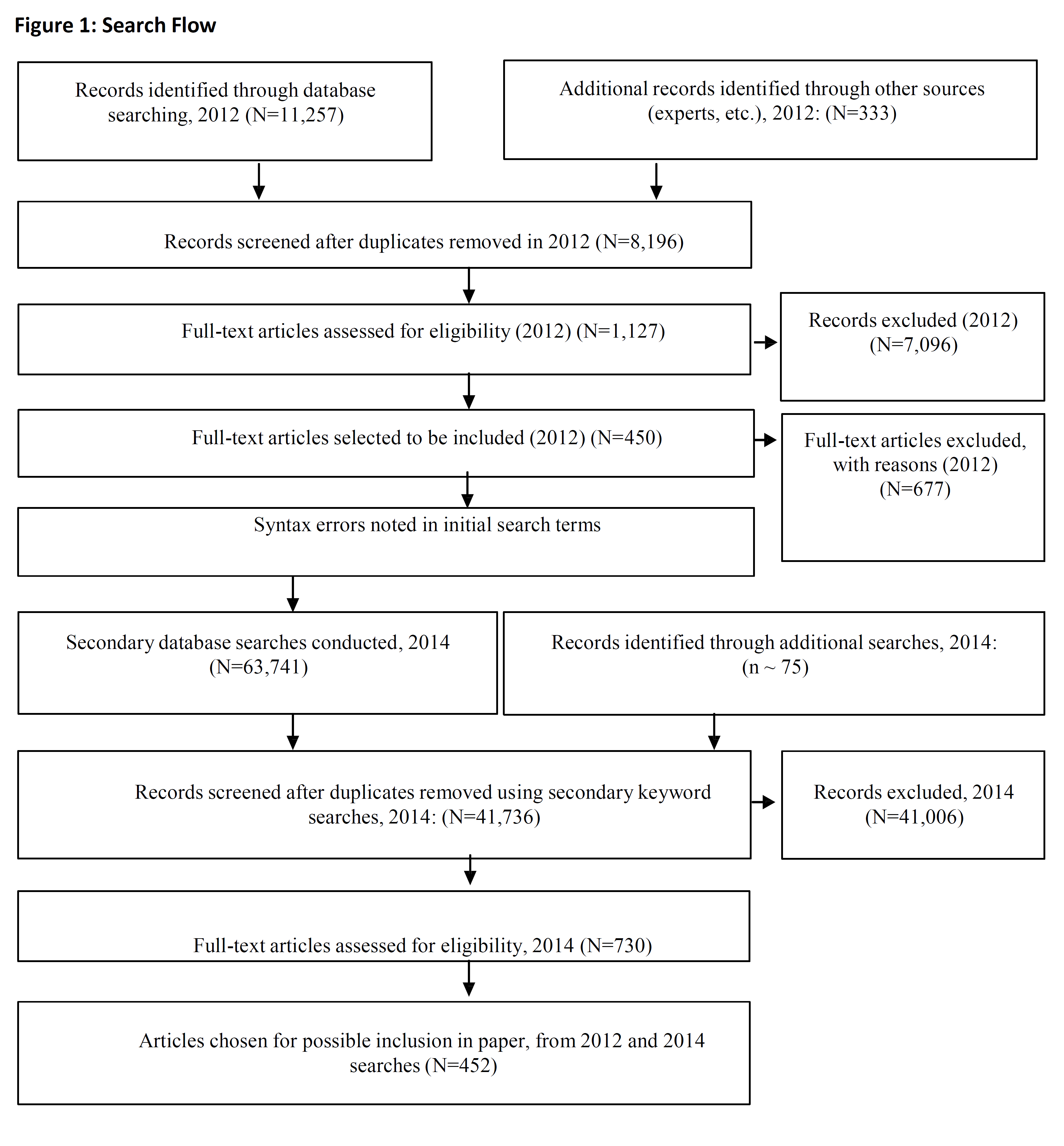

Supplement: S1 Note — (DOCX) [file pone.0160562.s002.docx]
